# SIRT6 Protects Smooth Muscle Cells From Senescence and Reduces Atherosclerosis

**DOI:** 10.1161/CIRCRESAHA.120.318353

**Published:** 2020-12-23

**Authors:** Mandy O.J. Grootaert, Alison Finigan, Nichola L. Figg, Anna K. Uryga, Martin R. Bennett

**Affiliations:** Division of Cardiovascular Medicine, University of Cambridge, United Kingdom.

**Keywords:** atherosclerosis, inflammation, metabolism, muscle cells, telomere

## Abstract

Vascular smooth muscle cell (VSMC) senescence promotes atherosclerosis and features of plaque instability, in part, through lipid-mediated oxidative DNA damage and telomere dysfunction. SIRT6 (Sirtuin 6) is a nuclear deacetylase involved in DNA damage response signaling, inflammation, and metabolism; however, its role in regulating VSMC senescence and atherosclerosis is unclear.

**Objective:**

We examined SIRT6 expression in human VSMCs, the role, regulation, and downstream pathways activated by SIRT6, and how VSMC SIRT6 regulates atherogenesis.

**Methods and Results:**

SIRT6 protein, but not mRNA, expression was markedly reduced in VSMCs in human and mouse atherosclerotic plaques, and in human VSMCs derived from plaques or undergoing replicative or palmitate-induced senescence versus healthy aortic VSMCs. The ubiquitin ligase CHIP (C terminus of HSC70-interacting protein) promoted SIRT6 stability, but CHIP expression was reduced in human and mouse plaque VSMCs and by palmitate in a p38- and c-Jun N-terminal kinase-dependent manner. SIRT6 bound to telomeres, while SIRT6 inhibition using shRNA or a deacetylase-inactive mutant (SIRT6^H133Y^) shortened human VSMC lifespan and induced senescence, associated with telomeric H3K9 (histone H3 lysine 9) hyperacetylation and 53BP1 (p53 binding protein 1) binding, indicative of telomere damage. In contrast, SIRT6 overexpression preserved telomere integrity, delayed cellular senescence, and reduced inflammatory cytokine expression and changes in VSMC metabolism associated with senescence. SIRT6, but not SIRT6^H133Y^, promoted proliferation and lifespan of mouse VSMCs, and prevented senescence-associated metabolic changes. ApoE^−/−^ (apolipoprotein E) mice were generated that overexpress SIRT6 or SIRT6^H133Y^ in VSMCs only. SM22α-hSIRT6/ApoE^−/−^ mice had reduced atherosclerosis, markers of senescence and inflammation compared with littermate controls, while plaques of SM22α-hSIRT6^H133Y^/ApoE^−/−^ mice showed increased features of plaque instability.

**Conclusions:**

SIRT6 protein expression is reduced in human and mouse plaque VSMCs and is positively regulated by CHIP. SIRT6 regulates telomere maintenance and VSMC lifespan and inhibits atherogenesis, all dependent on its deacetylase activity. Our data show that endogenous SIRT6 deacetylase is an important and unrecognized inhibitor of VSMC senescence and atherosclerosis.

## Abstract 2

Supplemental Digital Content is available in the text.

**In This Issue, see p 451**

**Meet the First Author, see p 452**

Vascular smooth muscle cells (VSMCs) comprise a major cellular component of the atherosclerotic plaque. VSMCs contribute to the lesion, but fibrous cap VSMCs are considered beneficial for plaque stability by reducing rupture. VSMCs accumulate in the intima through a phenotypic switch from a contractile to a synthetic phenotype, followed by migration and proliferation from the media (reviewed in Basatemur et al^1^). However, recent lineage-tracing studies demonstrate that only a small proportion of medial VSMCs proliferate extensively in atherosclerosis, and fibrous cap VSMCs show the highest numbers of proliferations (reviewed in Basatemur et al^1^). Recurrent rounds of cell division lead to telomere shortening and replicative senescence, while exposure to oxidative stress and accumulation of DNA damage also trigger VSMC senescence.^2^ Indeed, VSMCs in advanced human atherosclerotic lesions are characterized by DNA damage, senescence, and increased apoptosis (reviewed in Grootaert et al^3^).

SIRT6 (Sirtuin 6) is a member of the Sirtuin family of class III histone deacetylase enzymes and one of the few sirtuins located mainly in the nucleus. Sirtuins have chromatin remodeling activity that results in global transcriptional changes but can also directly modify nonhistone substrates.^4^ SIRT6 exerts its effects mostly by deacetylating histone 3 at the promoter region of its target genes, including those regulating DNA damage,^5,6^ telomere maintenance,^7^ apoptosis,^8^ inflammation,^9^ senescence,^10^ and glucose^11^ and lipid metabolism,^12,13^ all key pathways that are (mis)regulated during atherogenesis. SIRT6 polymorphisms have also been associated with increased atherosclerosis,^14^ but the mechanisms underlying any protective effect of SIRT6 against atherosclerosis, and whether VSMC SIRT6 expression or function are disrupted in human atherosclerosis are unclear.

We examined SIRT6 expression, role, regulation, and downstream consequences in human VSMCs. We also studied the effects of VSMC-specific overexpression of SIRT6 on atherosclerosis in mice and their dependence on its deacetylase activity. We identify a novel and unrecognized role for SIRT6 in protecting VSMCs from senescence through prevention of telomere damage that requires its deacetylase function, highlighting the therapeutical potential of SIRT6 in atherosclerosis.

## Methods

Please see the Major Resources Table in the Data Supplement. The data that support the findings of this study are available from the corresponding author upon reasonable request.

### Human Atherosclerotic Plaques and Aorta

Human tissues were obtained under informed consent using protocols approved by the University of Cambridge. Atherosclerotic plaques and normal aortas were obtained from patients undergoing carotid endarterectomy or aortic valve replacement, respectively.

### Cell Culture

Isolation and culture of human and mouse VSMCs are described in the Data Supplement.

### Cloning, Transfections, and Lentiviral Infections

All cloning strategies, transfections, and lentivirus infections of human VSMCs are described in the Data Supplement.

### Quantitative Real-Time Polymerase Chain Reaction

Quantitative polymerase chain reaction was performed as described and primer sequences were listed in Table I in the Data Supplement, both in the Data Supplement.

### Western Blotting and Immunoprecipitation

Western blotting and immunoprecipitation were performed as described using antibodies listed in Table II in the Data Supplement, both in the Data Supplement.

### Telomere Chromatin Immunoprecipitation

Telomere chromatin immunoprecipitation was performed using sonication to prepare sheared chromatin as described in the Data Supplement.

### Seahorse

Fatty acid oxidation and glycolysis were analyzed using a Seahorse XF96e flux analyzer (Agilent). Reagent details and calculation methods are described in the Data Supplement.

### Atherosclerosis Studies

All in vivo experiments were approved by the local animal Ethical Committee following UK Home Office licensing. Transgenic mice generation, genotyping protocols, power calculation, and animal randomization procedures are described in the Data Supplement. For atherosclerosis studies, 8-weeks-old male and female littermate control ApoE (apolipoprotein E)^−/−^, SM22α-hSIRT6/ApoE^−/−^, and SM22α-hSIRT6^H133Y^/ApoE^−/−^ mice were fed high-fat diet (HFD, 829-100, Special Diet Services, United Kingdom, 21% total fat, 0.2% cholesterol, and 0% sodium cholate) for 16 weeks. To assess SIRT6 and CHIP expression in mouse plaques, ApoE^−/−^ mice were fed chow or HFD for 16 weeks.

### Histology

Atherosclerotic plaque burden and composition were analyzed as described in the Data Supplement.

### Statistical Analysis

Data are shown as mean±SEM. All data represent independent data points and not technical replicates. Normality of distribution was determined using D’Agostino-Pearson normality tests or Shapiro-Wilk test if group size <8. Statistical significance was determined by 1-way ANOVA followed by a Dunnett post hoc test with multiple testing correction when 3 groups were compared, or a 2-way ANOVA for 2 independent variables. A 2-tailed unpaired Student *t* test was used to compare 2 groups of data. Where data were not normally distributed, a nonparametric test (Mann-Whitney *U* or Kruskal-Wallis test) was performed. Prism 9.0 (GraphPad Software, CA) was used and differences considered statistically significant when nominal *P*<0.05 or adjusted *P*<0.05 in case of multiple testing.

## Results

### SIRT6 Protein Expression Is Reduced in VSMCs in Human and Mouse Atherosclerosis and Upon Replicative and Palmitate-Induced Senescence

**Figure 1.**
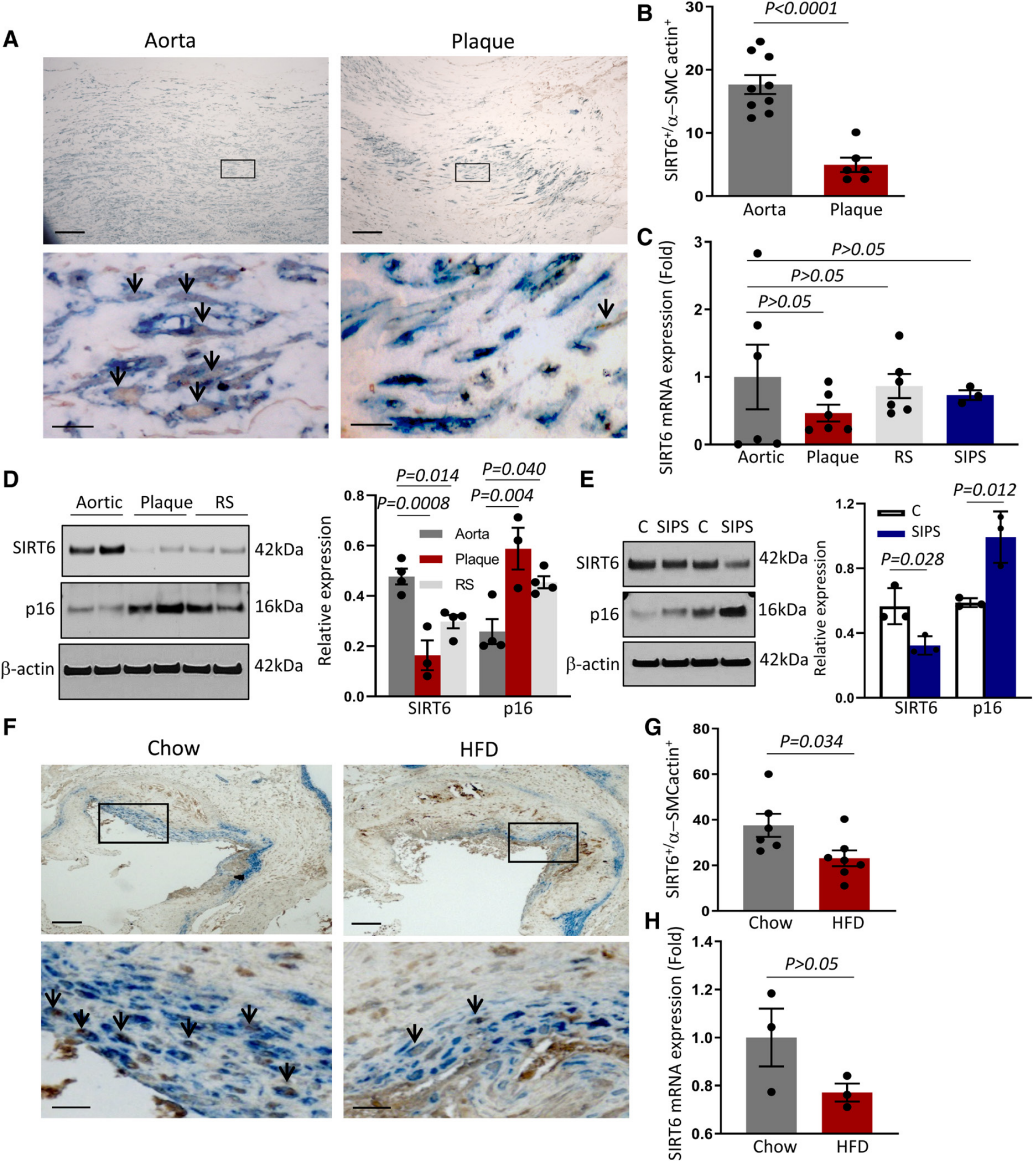
**SIRT6 (Sirtuin 6) protein expression is reduced in vascular smooth muscle cells (VSMCs) in human atherosclerosis and undergoing replicative and palmitate-induced senescence.** **A**, Immunohistochemistry and (**B**) quantification for α-smooth muscle cell (SMC)-actin (blue) and SIRT6 (brown) of VSMCs from human aorta (n=9) and carotid artery plaques (n=6; unpaired *t* test). High-power images of outlined areas indicate SIRT6^+^/α-SMC-actin^+^ cells (arrows); scale bar=250 µm (high-power views) and 25 µm (low-power views). **C**, Quantitative polymerase chain reaction (QPCR) for SIRT6 mRNA expression of human plaque VSMCs (n=6), or aortic VSMCs, either actively dividing (n=6), undergoing replicative senescence (RS; n=6) or palmitate-induced stress-induced premature senescence (SIPS; n=3; 1-way ANOVA, Dunnett post hoc). **D**, Western blot for SIRT6 and p16^ink4a^ of human plaque VSMCs (middle; n=3) and aortic VSMCs, either actively dividing (**left**) or undergoing RS (**right**; n=4) with quantification (1-way ANOVA, Dunnett post hoc). β-actin was used as loading control. **E**, Western blotting for SIRT6 and p16^ink4a^ and quantification of human VSMCs (hVSMCs) undergoing SIPS (unpaired *t* test, n=3). **F**, Immunohistochemistry and (**G**) quantification for α-SMC-actin (blue) and SIRT6 (brown) of VSMCs in plaques of chow or high-fat diet (HFD)-fed ApoE^−/−^ (apolipoprotein E) mice (unpaired *t* test, n=6–7). High-power images of outlined areas indicate SIRT6^+^/α-SMC-actin^+^ cells (arrows); scale bar=150 μm (high-power views) and 10 μm (low-power views). **H**, QPCR for SIRT6 mRNA of mouse aortas of chow or HFD-fed ApoE^−/−^ mice (unpaired *t* test, n=3). Data are shown as mean±SEM with nominal *P*<0.05 or multiplicity adjusted *P*<0.05.

We first examined SIRT6 expression in histologically normal human aortas and atherosclerotic plaques. Patients were between 60 and 75 years old and 63% were male, with no statistically significant differences between demographics. SIRT6 was detected in 18% nuclei of α-SMC-actin-positive cells in normal aortas, but only in 5% of plaque VSMCs (Figure 1A and 1B). Plaques are heterogeneous structures, and VSMC markers are lost in diseased vessels. We, therefore, cultured VSMCs from plaques and normal aortas and examined SIRT6 mRNA and protein expression in actively dividing aortic VSMCs, in aortic VSMCs undergoing replicative senescence (defined as cells undergoing >12 cumulative population doublings and completed >95% of their culture lifespan), or undergoing stress-induced premature senescence (SIPS) induced by transient exposure to the free fatty acid palmitate. Human VSMCs (hVSMCs) developed SIPS after 3 weeks following 24-hour palmitate treatment, characterized by reduced proliferation and increased expression of SaβG (Senescence-associated β-galactosidase) activity (Figure I in the Data Supplement). SIRT6 mRNA expression was similar in plaque and normal aortic VSMCs, including those undergoing replicative senescence and SIPS (Figure 1C). However, both plaque VSMCs and senescent aortic VSMCs showed markedly reduced SIRT6 protein expression versus healthy aortic VSMCs (Figure 1D and 1E), associated with upregulation of the senescence marker p16^ink4a^. Similarly, SIRT6 was expressed in 23% nuclei of α-SMC-actin^+^ cells in aortic root plaques of HFD-fed ApoE^−/−^ mice versus 37% of chow-fed mice (Figure 1F and 1G). However, SIRT6 mRNA expression was not reduced in aortas of HFD versus chow-fed ApoE^−/−^ mice (Figure 1H).

**Figure 2.**
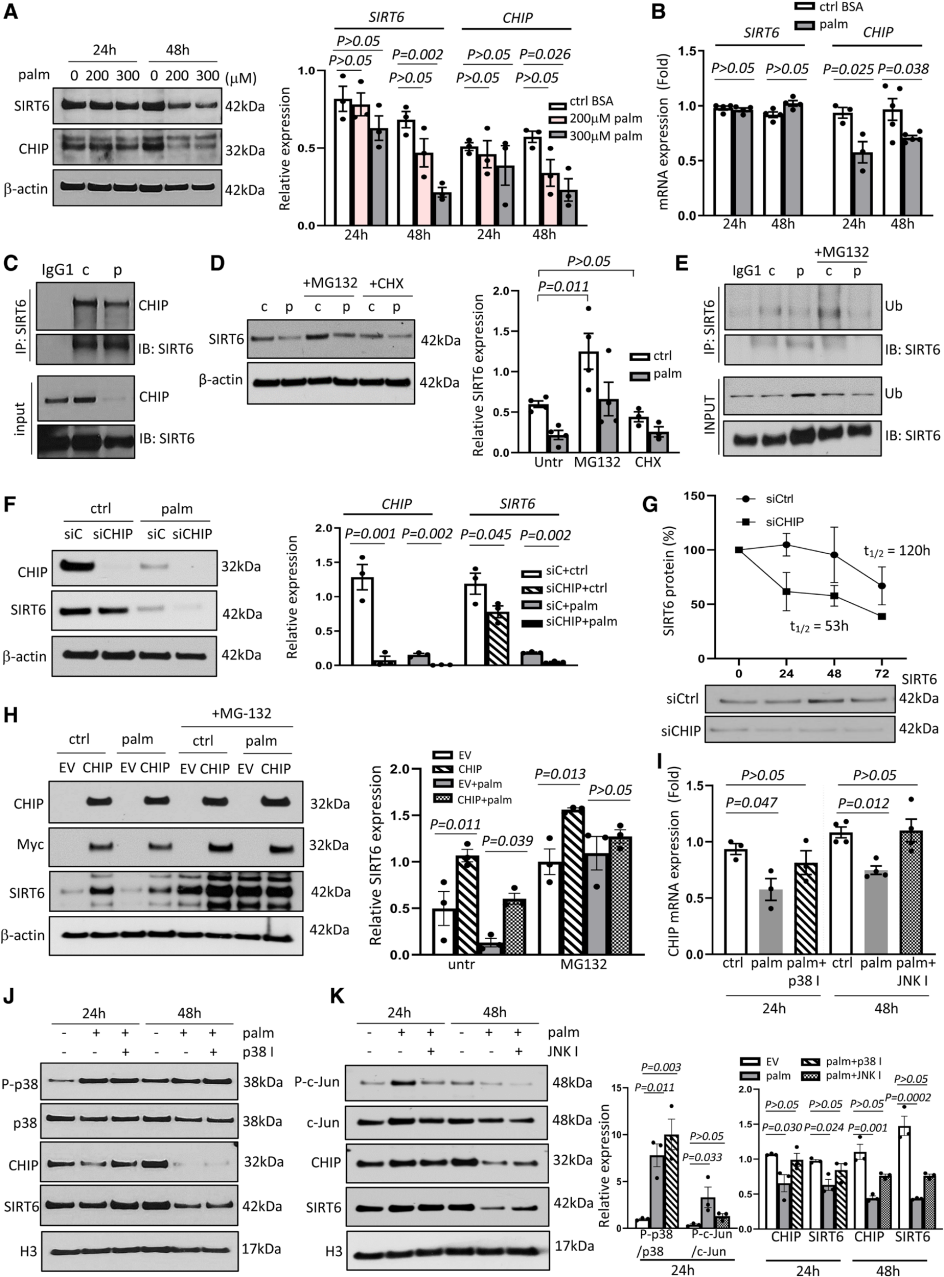
**SIRT6 (Sirtuin 6) protein stability is positively regulated by ubiquitin ligase CHIP (C terminus of HSC70-interacting protein).** **A**, Western blot for SIRT6 and CHIP in human vascular smooth muscle cells (hVSMCs) treated with 0, 200, or 300 µmol/L palmitate (palm) for 24 or 48 h, and quantification (2-way ANOVA, Tukey post hoc, n=3). β-actin was used as loading control. **B**, Quantitative polymerase chain reaction (QPCR) for SIRT6 and CHIP mRNA expression of hVSMCs treated with 300 µmol/L palmitate or control BSA for 24 or 48 h (2-way ANOVA, Bonferroni post hoc, n=3–5). **C**, Immunoprecipitation for SIRT6 with hVSMCs treated with palmitate (p) or BSA (c) for 48 h, followed by Western blotting for CHIP and SIRT6. IgG1κ used as isotype control. **D**, Western blot for SIRT6 in hVSMCs treated with palmitate or BSA for 48 h, with/without proteasome inhibitor MG132 (1 µmol/L) or cycloheximide (CHX, 1 mg/mL), and quantification (2-way ANOVA, Tukey post hoc, n=3). **E**, Immunoprecipitation for SIRT6 with hVSMCs treated with palmitate (p) or BSA (c) for 48 h, with/without MG132 (1 µmol/L), followed by Western blotting for ubiquitin and SIRT6. **F**, Western blot with quantification for CHIP and SIRT6 expression in hVSMCs after transient silencing of CHIP (siCHIP) vs control siRNA (siCtrl) followed by 48 h palmitate or BSA treatment (1-way ANOVA, Tukey post hoc, n=3). **G**, SIRT6 half-life determined by Western blotting after siCHIP vs siCtrl at 0, 24, 48, and 72 h (n=3). **H**, Western blot for CHIP, Myc-tag, and SIRT6 in hVSMCs infected with a lentivirus expressing Myc-tagged CHIP vs empty vector (EV), treated with palmitate (or BSA) for 48 h, and quantification (2-way ANOVA, Tukey post hoc, n=3). **I**, QPCR for CHIP mRNA expression in hVSMCs treated with palmitate (or BSA) for 24 h with/without p38-inhibitor (p38 I, 1 µmol/L) or for 48 h with/without JNK (c-Jun N-terminal kinase)-inhibitor (40 µmol/L; 1-way ANOVA, Dunnett post hoc, n=3–4). **J** and **K**, Western blot analysis for phospho-p38, p38 (**J**) phospho-c-Jun, total c-Jun (**K**), CHIP and SIRT6 expression in hVSMCs treated with palmitate (or BSA) for 24 and 48 h, with/without (**J**) p38 I or (**K**) JNK I, and quantification (1-way ANOVA, Dunnett post hoc, n=3). Histone 3 was used as loading control. Data are shown as mean±SEM with multiplicity adjusted *P*<0.05.

This data suggests that SIRT6 expression in VSMCs is negatively regulated by HFD and the free fatty acid palmitate. We, therefore, examined SIRT6 expression after acute exposure to palmitate, and thus before the onset of palmitate-induced senescence. Palmitate led to a dose- and time-dependent reduction in SIRT6 protein but not SIRT6 mRNA expression (Figure 2A and 2B), suggesting post-transcriptional regulation. Palmitate caused accumulation of the DNA damage marker gH2AX (gamma H2A.X variant histone) at 48 hours, but this did not precede downregulation of SIRT6 (Figure I in the Data Supplement). In addition, DNA damage induced either by the topoisomerase inhibitor doxorubicin or expression of a dysfunctional TRF2 (telomeric repeat-binding factor 2) protein (TRF2^T188A^ which induces persistent telomere damage^15^) did not reduce SIRT6 (Figure I in the Data Supplement), suggesting that SIRT6 downregulation occurs before DNA damage and cell senescence. Indeed, TRF2^T188A^ increased SIRT6 expression, supporting SIRT6’s role as a DNA damage sensor.^5^ Palmitate did not reduce SIRT1, which also protects against DNA damage and is downregulated upon fat feeding,^16^ and treatment of hVSMCs with other lipid-species including native or oxidized LDL (low-density lipoprotein) did not affect SIRT6 protein expression (Figure II in the Data Supplement). Palmitate uptake may occur through both passive and fatty acid transporter-mediated transport.^17^ However, silencing of the fatty acid transporter CD36 did not affect SIRT6 expression in the presence of palmitate (Figure II in the Data Supplement).

### The Ubiquitin Ligase CHIP Positively Regulates SIRT6 Stability in Human VSMCs, but Its Expression Is Reduced in Plaque VSMCs

The ubiquitin ligase CHIP (C terminus of HSC70-interacting protein) can bind to SIRT6 and positively regulate SIRT6 protein stability in human embryonal kidney cells.^18^ Interestingly, CHIP protein expression was reduced upon palmitate treatment in parallel with SIRT6, but CHIP mRNA expression was also decreased (Figure 2A and 2B). Immunoprecipitation showed that CHIP directly interacts with SIRT6 in hVSMCs, and this interaction was reduced upon palmitate treatment (Figure 2C). SIRT6 expression increased after treatment with the proteasome inhibitor MG132 but not with the protein synthesis inhibitor cycloheximide (Figure 2D), suggesting that SIRT6 expression is mainly determined by protein stability and proteasomal degradation. Palmitate reduced SIRT6 protein expression (Figure 2D) and SIRT6 ubiquitination (Figure 2E) even in the presence of MG132, suggesting that palmitate regulates SIRT6 expression upstream of the proteasome, possibly through reducing CHIP. We, therefore, silenced or stably overexpressed CHIP in hVSMCs and evaluated SIRT6 stability in the presence and absence of palmitate. Transient silencing of CHIP moderately reduced SIRT6 protein expression (Figure 2F). Palmitate markedly reduced both CHIP and SIRT6 protein expression in control siRNA-treated cells, and further decreased SIRT6 in CHIP-depleted cells compared with controls (Figure 2F). SIRT6 protein expression was stable for up to 48 hours in basal conditions, but silencing of CHIP accelerated SIRT6 degradation at 24 hours and reduced SIRT6 half-life from 120h to 53 hours (Figure 2G), confirming that CHIP regulates SIRT6 protein stability. Stable overexpression of Myc-tagged CHIP using a lentivirus vector increased SIRT6 protein expression compared with an empty vector in untreated cells and prevented palmitate-induced SIRT6 reduction (Figure 2H). MG132 increased SIRT6 protein levels further in cells overexpressing CHIP indicating that CHIP reduces SIRT6 proteasomal degradation. Of note, silencing or overexpression of CHIP did not affect SIRT6 mRNA expression with or without palmitate treatment (Figure III in the Data Supplement).

To understand how palmitate regulates SIRT6 through CHIP, we investigated how CHIP itself is regulated. CHIP protein expression was similar in control cells with or without MG132 treatment, suggesting that CHIP is not degraded via the proteasome (Figure III in the Data Supplement). Palmitate can activate the stress-activated protein kinases p38, JNK (c-Jun N-terminal kinase), and Erk1/2 (extracellular signal-regulated kinases) in cardiomyocytes,^19^ which could mediate downregulation of CHIP. Indeed, inhibition of p38 or JNK blocked palmitate-induced downregulation of CHIP mRNA (Figure 2I). Palmitate induced phosphorylation of p38 and c-Jun (Figure 2J and 2K) but completely inhibited Erk1/2 phosphorylation (Figure III in the Data Supplement). p38 inhibition almost fully recovered CHIP and SIRT6 protein in parallel at 24 hours (Figure 2J), but JNK inhibition only partially recovered CHIP and SIRT6 protein after 48 hours (Figure 2K). Overall, our data show that both p38 and JNK pathways mediate palmitate-induced reduction of CHIP and SIRT6 in hVSMCs.

Finally, we investigated the expression of CHIP in human and mouse atherosclerosis (Figure IV in the Data Supplement). CHIP expression was detected in 33% of α-SMC-actin^+^ cells in human plaques versus 59% in normal aorta, and cultured plaque hVSMCs showed reduced CHIP mRNA expression versus normal aortic hVSMCs. In mouse atherosclerosis, 43% of α-SMC-actin^+^ cells expressed CHIP in plaques of HFD-fed ApoE^−/−^ mice versus 66% in chow-fed mice, and HFD-fed ApoE^−/−^ mice showed reduced CHIP mRNA expression in their aortas versus chow-fed mice. CHIP expression in α-SMC-actin^+^ cells in mouse plaques correlated significantly with SIRT6 expression, segregating the HFD from the control group.

Overall, our data indicate that the CVD-risk associated fatty acid palmitate reduces CHIP mRNA and protein through p38 and JNK activation, with subsequent downregulation of SIRT6 through reduced CHIP-mediated stability of SIRT6 protein. This mechanism may explain the downregulation of CHIP and SIRT6 seen in VSMCs in human and mouse atherosclerosis. The downregulation of SIRT6 upon palmitate is persistent and occurs before palmitate-induced DNA damage and senescence, suggesting that SIRT6 may be a major regulator of DNA damage and senescence in VSMCs.

### SIRT6 Delays VSMC Senescence

**Figure 3.**
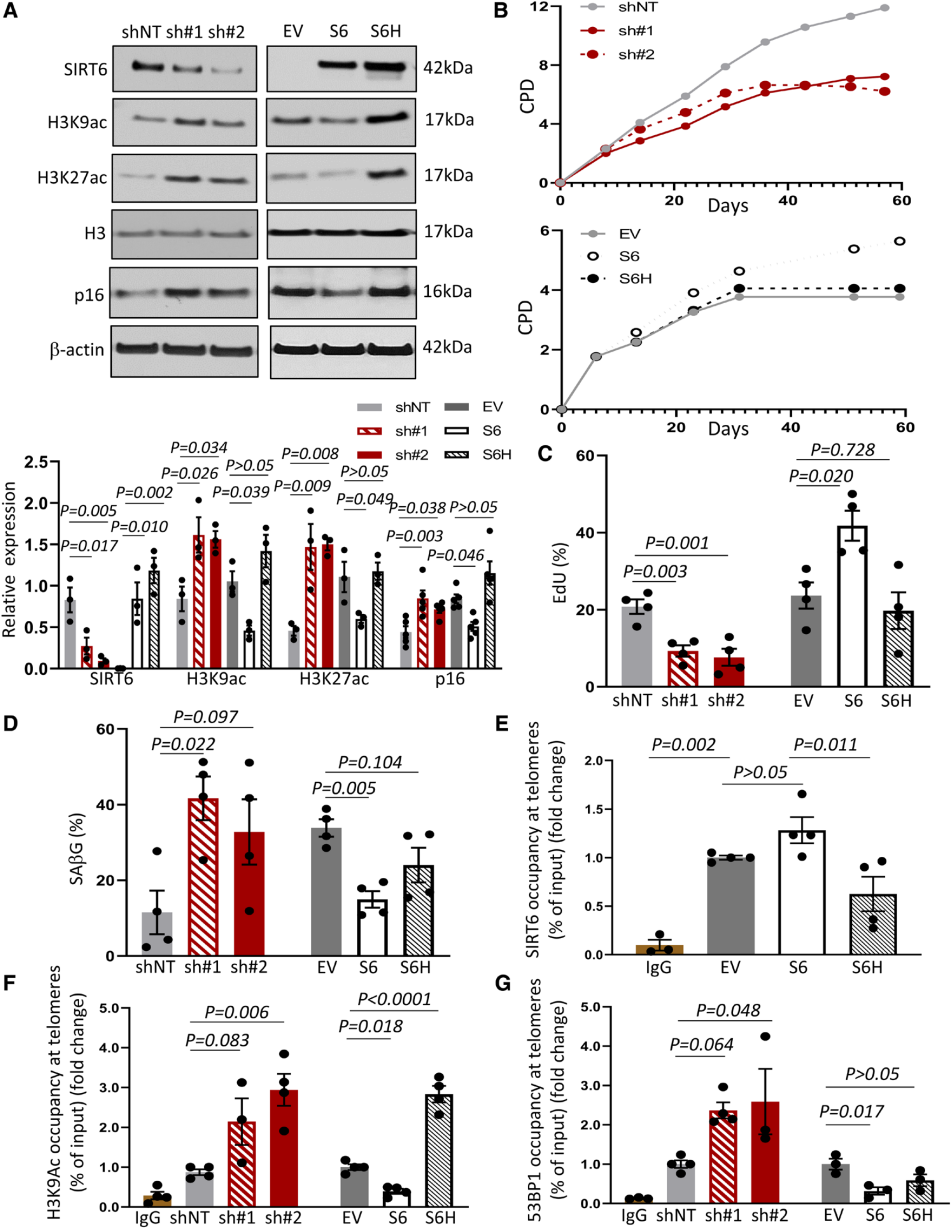
**SIRT6 (Sirtuin 6) delays senescence, binds to telomeres and regulates telomeric H3K9 (histone H3 lysine 9) deacetylation and DNA damage repair (DDR) signaling of human vascular smooth muscle cells (hVSMCs).** **A**, Western blot for SIRT6, H3K9ac (acetylated H3K9), H3K27ac (acetylated H3K27), and p16 in hVSMCs expressing shRNA against SIRT6 (sh#1 and sh#2) or overexpressing SIRT6 (S6) or its catalytic inactive mutant (SIRT6^H133Y^, S6H) with quantification (1-way ANOVA, Dunnett post hoc, n=3–5). Total histone 3 and β-actin were used as loading control. **B**, Representative cumulative population doublings (CPD) of SIRT6-depleted (**top**) or SIRT6/SIRT6^H133Y^ (**bottom**) hVSMCs. **C**, % 5-ethynyl-2’-deoxyuridine (EdU) incorporation and (**D**) % SAβG (senescence-associated beta galactosidase)-positive hVSMCs in late-passage experimental cell lines (p10-12; 1-way ANOVA, Dunnett post hoc, n=4). **E**, Telomere chromatin immunoprecipitation (Telo-ChIP) analysis for SIRT6 in hVSMCs overexpressing SIRT6 or expressing SIRT6^H133Y^ (1-way ANOVA, Tukey post hoc, n=4) at early passage (p4-6). **F** and **G**, Telo-ChIP analysis for H3K9ac (**F**), and 53BP1 (p53 binding protein 1; **G**) in SIRT6-depleted or SIRT6/SIRT6^H133Y^ hVSMCs at early passage (p4-6; 1-way ANOVA, Dunnett post hoc, n=3–4). Data are shown as mean±SEM with multiplicity adjusted *P*<0.05. SAβG indicates senescence-associated β galactosidase.

To investigate the role of SIRT6 in hVSMC senescence, we generated human VSMCs with stable knockdown of SIRT6 using 2 different species of shRNA (sh#1 or sh#2), or stable overexpression of either wild-type (WT) SIRT6 or expression of a catalytic inactive deacetylase-incompetent mutant (SIRT6^H133Y^), using lentivirus-based gene transfer (Figure 3A). SIRT6 silencing induced hyperacetylation of H3K9 and H3K27 (histone 3 at K9 and K27), the 2 main histone 3 targets of SIRT6. SIRT6 overexpression reduced H3K9 and H3K27 acetylation, while similar to knockdown, SIRT6^H133Y^ expression increased H3K9 and H3K27 acetylation (Figure 3A). Silencing or overexpression of either SIRT6 form did not affect mRNA expression of other sirtuins or CHIP expression, suggesting that there is no positive feedback mechanism between SIRT6 and CHIP (Figure V in the Data Supplement).

We next examined cumulative population doublings, cell proliferation, and senescence markers in hVSMCs with stable knockdown or overexpression of SIRT6, or expression of SIRT6^H133Y^. SIRT6 silencing upregulated p16^ink4a^, shortened culture lifespan, reduced cell proliferation, and increased SAβG (senescence-associated beta galactosidase) activity (Figure 3A through 3D and Figure VI in the Data Supplement). In contrast, SIRT6 overexpression reduced p16^ink4a^, prolonged culture lifespan, stimulated VSMC proliferation, and inhibited SAβG activity versus empty vector control cells; the antisenescence and lifespan-extending effects of SIRT6 were lost in hVSMCs expressing SIRT6^H133Y^ (Figure 3A through 3D and Figure VI in the Data Supplement).

### SIRT6 Preserves Telomere Integrity by Regulating Telomere Histone Deacetylation and Preventing Telomere DNA Damage

SIRT6 exerts multiple cell type-specific effects, including on cell death,^8^ DNA damage,^5,6^ telomere maintenance,^7^ inflammation,^9^ and cell metabolism,^11–13^ all of which could affect VSMC senescence. However, knockdown or overexpression of SIRT6 or expression of SIRT6^H133Y^ had no effect on apoptosis in early passage hVSMCs, either basally or after H_2_O_2_ treatment, or on the expression of the global DNA damage marker gH2AX (Figure VI in the Data Supplement).

Replicative senescence of hVSMCs in culture is associated with loss or damage to telomeres, and interventions that promote or inhibit telomere damage shorten or lengthen VSMC culture lifespan, respectively.^15,20^ We, therefore, explored the potential role of SIRT6 in VSMC telomere maintenance using telomere chromatin immunoprecipitation on early passage cells. Endogenous SIRT6 could bind telomeres in hVSMCs, as shown by increased binding of antibodies to SIRT6 versus control IgG. SIRT6 telomere binding was similar in SIRT6-overexpressing cells but was significantly reduced in SIRT6^H133Y^ versus SIRT6 hVSMCs (Figure 3E), despite SIRT6^H133Y^ VSMCs showing marginally higher expression than SIRT6 VSMCs (Figure 3A). Next, we assessed whether loss of SIRT6 telomere binding affects acetylation of telomeric histones. SIRT6-depleted hVSMCs had increased telomere H3K9 acetylation versus shRNA controls; in contrast, SIRT6 overexpression reduced H3K9 acetylation at telomeric DNA while SIRT6^H133Y^-expressing cells showed massive telomeric H3K9 hyperacetylation (Figure 3F). To investigate the downstream effects on telomere integrity, we performed telomere chromatin immunoprecipitation for the DNA damage response protein 53BP1 (p53 binding protein 1), which facilitates nonhomologous end joining of damaged chromatin ends, thereby promoting telomere dysfunction.^21^ SIRT6-depleted hVSMCs showed increased telomere binding of 53BP1 versus shRNA controls, indicating telomere damage. In contrast, SIRT6-overexpressing hVSMCs exhibited decreased 53BP1 telomeric binding compared with empty vector cells, which was not evident in SIRT6^H133Y^ VSMCs (Figure 3G). Of note, expression of the shelterin complex proteins TRF1 and TRF2 that protect telomere ends was not changed with SIRT6 knockdown or in SIRT6 or SIRT6^H133Y^ VSMCs (Figure VI in the Data Supplement). These data suggest that SIRT6, including its catalytic activity, is required for maintaining telomere integrity, while loss of SIRT6 or its deacetylation activity leads to telomere damage.

### SIRT6 Partially Reverses the Metabolic Phenotype of Senescent hVSMCs

**Figure 4.**
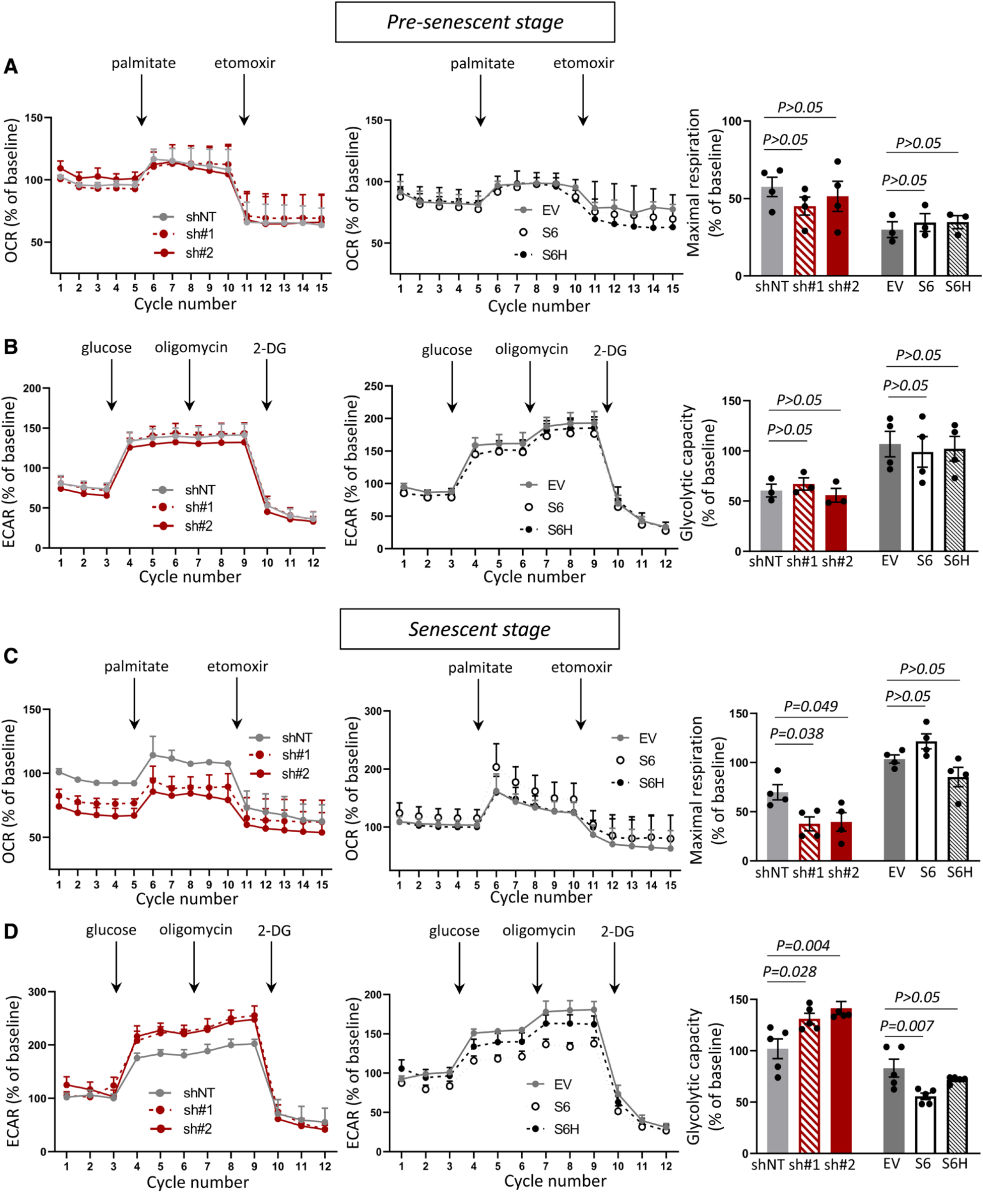
**SIRT6 (Sirtuin 6) prevents senescence-associated changes in cell metabolism.** **A–D**, Seahorse extracellular flux profiles of human vascular smooth muscle cells (hVSMCs) expressing shRNA against SIRT6 (sh#1 and sh#2) or overexpressing SIRT6 (S6) or SIRT6^H133Y^ (S6H) in the presenescent (p4-6; **A** and **B**) and senescent (p10-12) stage (**C** and **D**). Seahorse tracings are presented as % of baseline, after normalization to protein (**left**). **A** and **C**, Fatty acid oxidation was measured by oxygen consumption rate (OCR) upon addition of palmitate (2 mmol/L) and etomoxir (240 µmol/L). **B** and **D**, Glycolysis was measured by extracellular acidification rate (ECAR) upon glucose (10 mmol/L), oligomycin (2 µg/mL), and 2-deoxyGlucose (2DG, 50 mmol/L). Maximal respiration and glycolytic capacity are shown in right panels (1-way ANOVA, Dunnett post hoc, n=3–5). Data are shown as mean±SEM with multiplicity adjusted *P*<0.05.

SIRT6 transcriptionally regulates fatty-acid^12,13^ and glucose metabolism^11^ by H3K9 deacetylation of a range of fatty acid b-oxidation (FAO) and glycolysis genes. Similar to human atherosclerotic plaque VSMCs,^22,23^ senescent cells show a marked shift in substrate utilization for energy, including a switch from respiration to glycolysis.^24,25^ However, it is unclear whether metabolic effects of SIRT6 regulate VSMC senescence directly or are evident only when the metabolic phenotype of senescence is established. Early passage presenescent (actively proliferating) VSMCs expressing SIRT6, SIRT6^H133Y^, or after SIRT6 shRNA knockdown showed no significant differences in FAO or glycolysis (Figure 4A and 4B), either at baseline or during maximal respiration or glycolysis. In addition, quantitative polymerase chain reaction revealed no consistent changes in transcripts of a range of FAO and glycolysis genes in actively proliferating hVSMCs (Figure VII in the Data Supplement). In contrast, SIRT6-depleted hVSMCs showed marked changes in their metabolic profile at senescence, including decreased FAO and increased glycolysis (Figure 4C and 4D). hVSMCs overexpressing SIRT6, but not SIRT6^H133Y^, showed reduced glycolysis. These data suggest that metabolic effects of SIRT6 do not directly regulate senescence via SIRT6-dependent transcriptional regulation of FAO and glycolysis genes; rather the metabolic changes observed at senescence of SIRT6-depleted hVSMCs are a consequence of the premature senescence of these cells.

### SIRT6 Partially Suppresses Inflammation in Senescent hVSMCs

**Figure 5.**
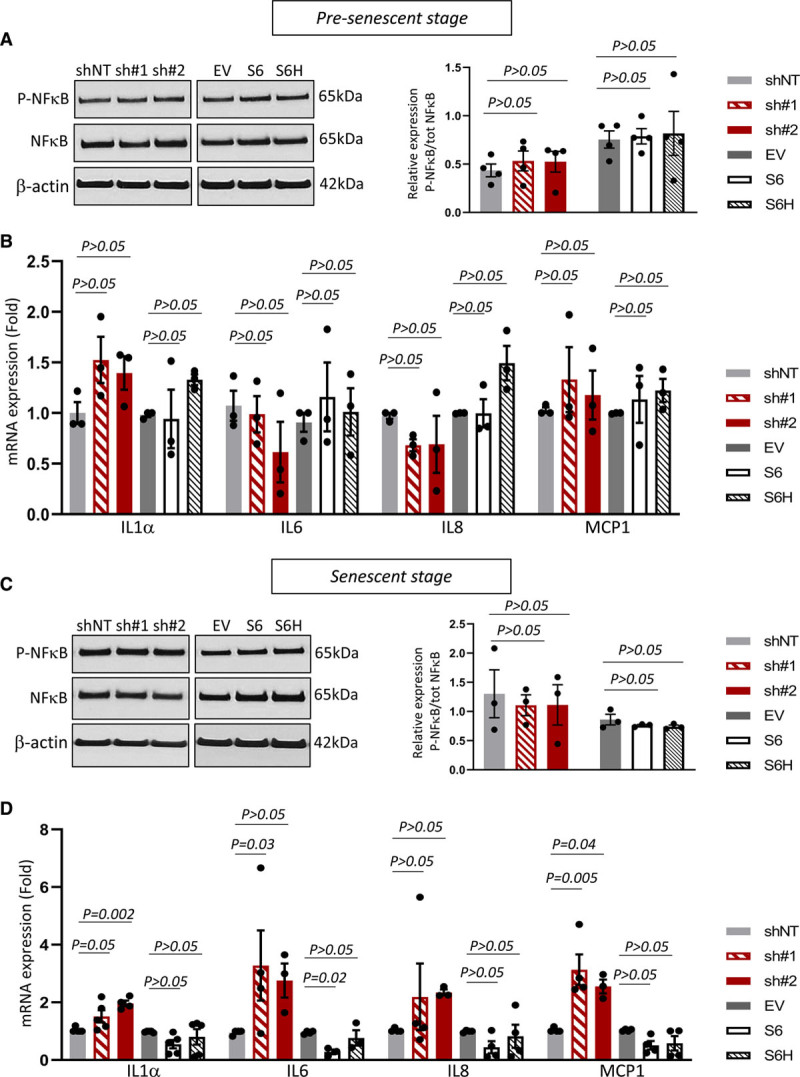
**SIRT6 (Sirtuin 6) partially suppresses inflammation in senescent human vascular smooth muscle cells (hVSMCs).** **A** and **C**, Western blot for phospho-NF-κB (nuclear factor-κB) p65 and total NF-κB p65 of hVSMCs expressing shRNA against SIRT6 (sh#1 and sh#2), overexpressing SIRT6 (S6) or expressing SIRT6^H133Y^ (S6H) at presenescent (p4-6; **A**) or senescent (p10-12; **C**) stage (1-way ANOVA, Dunnett post hoc, n=3–4). **B** and **D**, Quantitative polymerase chain reaction (QPCR) for expression of proinflammatory cytokines of experimental cell lines at presenescent (n=3; **B**) or senescent stage (n=4; **D**; 1-way ANOVA, Dunnett post hoc). Data are shown as mean±SEM with multiplicity adjusted *P*<0.05. IL indicates interleukin; and MCP-1, monocyte chemoattractant protein 1.

Senescent VSMCs may promote atherosclerosis at least, in part, via upregulation of inflammatory cytokines, as part of the senescence-associated secretory phenotype.^26^ SIRT6 exerts anti-inflammatory effects by repressing NF-κB (nuclear factor-κB)-dependent inflammatory gene expression through H3K9 deacetylation at chromatin,^9^ and loss of SIRT6 triggers NF-κB-dependent senescence in HeLa cells; we, therefore, investigated whether SIRT6 regulates inflammation in hVSMCs. Contrary to other studies,^9^ presenescent VSMCs with SIRT6 knockdown, overexpression of SIRT6 or expression of SIRT6^H133Y^ did not show NF-κB activation (Figure 5A), or changes in expression of IL (interleukin)-1α, IL-6, IL-8, and MCP-1 (monocyte chemoattractant protein 1) mRNA (Figure 5B). Despite no changes in NF-κB phosphorylation (Figure 5C), SIRT6-depleted senescent VSMCs showed a significant upregulation of IL-1α, IL-6, and MCP-1 mRNA expression (Figure 5D). In contrast, late-stage SIRT6 hVSMCs showed reduced IL-6 mRNA expression compared with empty vector cells that had reached senescence, which was not observed in SIRT6^H133Y^ hVSMCs. These data show that the anti-inflammatory effect of SIRT6 does not regulate senescence directly but that SIRT6 partially suppresses inflammation by delaying VSMC senescence.

### Characterization of VSMC-Specific SIRT6 or SIRT6^H133Y^ Transgenic Mice

**Figure 6.**
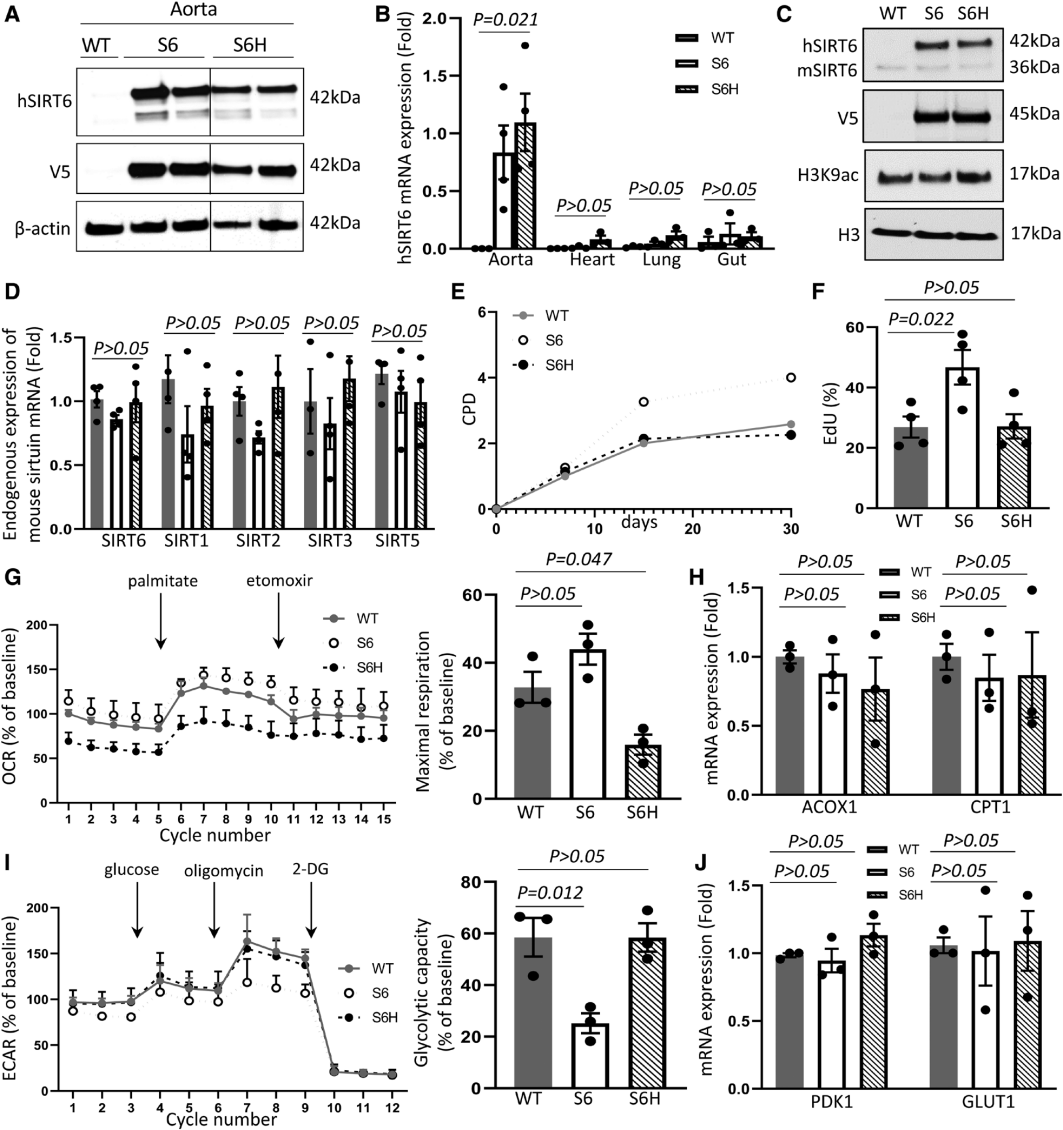
**Characterization of mice and mouse vascular smooth muscle cells (VSMCs) expressing human SIRT6 (Sirtuin 6) or SIRT6^H133Y^.** **A**, Western blot for hIRT6 and V5 in aortas of wild-type (WT) mice or mice expressing hSIRT6 (S6) or SIRT6^H133Y^ (S6H). **B**, Quantitative polymerase chain reaction (QPCR) analysis of SMC-rich tissues (aorta, heart, lung, and gut) for hSIRT6 in experimental mice (1-way ANOVA, nominal *P*<0.05 shown, n=4). **C**, Western blotting for human and mouse SIRT6, V5 and H3K9ac (acetylated histone H3 lysine 9) in aortic VSMCs isolated from experimental mice. **D**, QPCR for mouse endogenous SIRT6, SIRT1, SIRT2, SIRT3, and SIRT5 (1-way ANOVA, nominal *P*<0.05 shown, n=4). mVSMC culture lifespan assessed by cumulative population doublings (CPD; **E**) and cell proliferation by 5-ethynyl-2’-deoxyuridine (EdU) incorporation (**F**; 1-way ANOVA, Dunnett post hoc, n=4). **G** and **I**, Seahorse profiles for fatty acid oxidation (FAO; **G**) and glycolysis (**I**) and maximal respiration and glycolytic capacity (1-way ANOVA, Dunnett post hoc, n=3). **H** and **J**, QPCR for selected FAO (**H**) and glycolysis (**J**) genes. Data are shown as mean±SEM with multiplicity adjusted *P*<0.05. ACOX1 indicates acyl-CoA-oxidase-1; CPT1, carnitine-palmitoyltransferase-1; GLUT1, glucose transporter 1; and PDK1, pyruvate dehydrogenase kinase 1.

To investigate the role of SIRT6 in VSMCs in vivo, we generated C57/BL6 mice that overexpress V5-tagged human SIRT6 or SIRT6^H133Y^ from the CArG-box deleted minimal SM22α promoter (SM22α-hSIRT6 or SM22α-hSIRT6^133Y^ mice) that is only expressed in arterial vascular VSMCs^27^ and is not downregulated upon VSMC phenotypic switching.^28^ Two founders of each line were studied, which each behaved similarly. Transgenic mice showed expression of either human SIRT6 or SIRT6^H133Y^ (Figure 6A) that was restricted to the aorta (Figure 6B). Aortic VSMCs derived from these mice showed similar levels of expression of human SIRT6 and SIRT6^H133Y^ without affecting mouse endogenous SIRT6 expression (Figure 6C). mVSMCs expressing hSIRT6^H133Y^ showed H3K9 hyperacetylation while hSIRT6-expressing mVSMCs showed a moderate reduction in H3K9 acetylation compared with WT cells, confirming activity of hSIRT6 and hSIRT6^H133Y^ in mouse VSMCs, and that hSIRT6^H133Y^ is a suitable control for expression of hSIRT6. Expression of hSIRT6 or hSIRT6^H133Y^ in mouse VSMCs also did not affect endogenous levels of other sirtuins (Figure 6D). Mouse hSIRT6 VSMCs showed prolonged culture lifespan and increased proliferation (Figure 6E and 6F), no change in FAO (Figure 6G) or the FAO genes acyl-CoA oxidase-1 and carnitine-palmitoyltransferase 1 (Figure 6H), decreased glycolysis (Figure 6I), but no change in glycolysis genes PDK1 (pyruvate dehydrogenase kinase 1) and GLUT1 (glucose transporter 1) when compared with WT mVSMCs (Figure 6J). In contrast, hSIRT6^H133Y^ mVSMCs behaved similar to WT mVSMCs for culture lifespan (Figure 6E), proliferation (Figure 6F), and glycolysis (Figures 6I and 6J). Only FAO was significantly decreased in hSIRT6^H133Y^ mVSMCs compared with WT mVSMCs, but without significant changes in expression of selected FAO-related genes (Figures 6G and 6H).

### SIRT6 Reduces Atherosclerotic Plaque Burden, Inflammation and Senescence, and Preserves Markers of Plaque Stability

**Figure 7.**
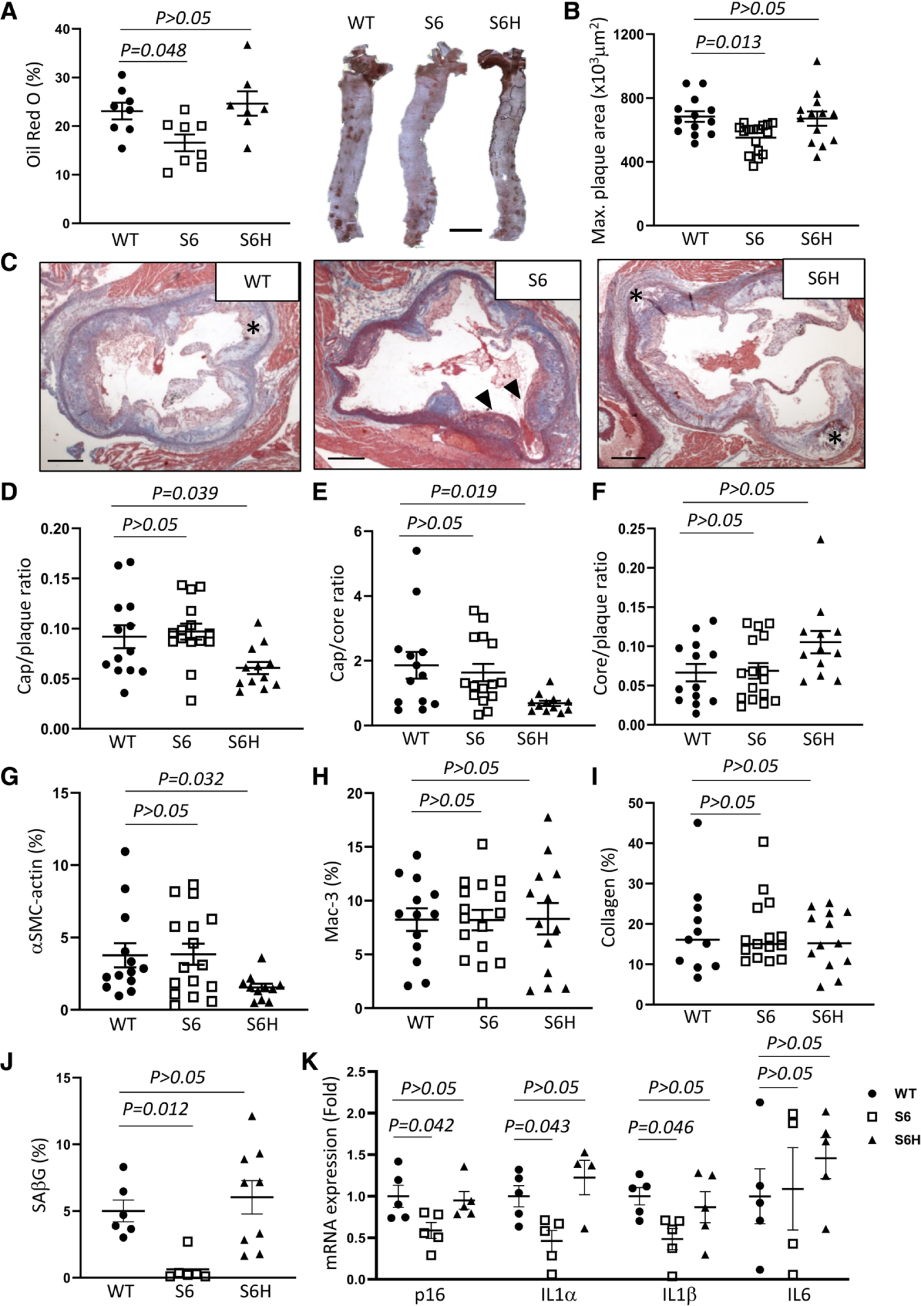
**Vascular smooth muscle cell (VSMC) SIRT6 (Sirtuin 6) protects against development of atherosclerosis.** **A**, Oil Red O (ORO) staining of descending aorta and quantification of ORO-positive area in littermate ApoE^−/−^ (apolipoprotein E) (wild type [WT]), SM22α-SIRT6/ApoE^−/−^ (S6), and SM22α-hSIRT6^H133Y^/ApoE^−/−^ (S6H) mice fed a high-fat diet (HFD) for 16 weeks (1-way ANOVA, Dunnett post hoc, n=7–8). Scale bar=3 mm. **B**, Plaque area at the maximum area of aortic root plaques of experimental mice (1-way ANOVA, Dunnett post hoc, n=13–16). **C**, Representative images of aortic root plaques stained with Masson trichrome. Asterisks and black arrowheads indicate necrotic cores and fibrous caps, respectively. Scale bar=150 µm. Analysis of cap/plaque (**D**), cap/core (**E**), and core/plaque (**F**) ratios (1-way ANOVA, Dunnett post hoc [**D**] and Kruskal-Wallis, Dunn post hoc [**E–F**], n=13–16). Analysis of % α-SMC-actin (**G**), % Mac-3 (**H**), and % collagen (**I**)–positive areas in aortic plaques (Kruskal-Wallis, Dunn post hoc [**G** and **I**] and 1-way ANOVA, Dunnett post hoc [**H**], n=13–16). **J**, Quantification of SAβG (senescence-associated β-galactosidase)-positive area in plaques of brachiocephalic artery (Kruskal-Wallis, Dunn post hoc, n=6–9). **K**, mRNA expression of p16 and senescence-associated secretory phenotype cytokines in aortas of experimental mice (1-way ANOVA, Dunnett post hoc, n=5). Data are shown as mean±SEM with multiplicity adjusted *P*<0.05. IL indicates interleukin; and SAβG, senescence-associated β galactosidase.

To study the effects of SIRT6 on atherogenesis, SIRT6 and SIRT6^H133Y^ transgenic mice were crossed onto the ApoE^−/−^ background. Littermate control ApoE^−/−^, SM22α-SIRT6/ApoE^−/−^ and SM22α-hSIRT6^H133Y^/ApoE^−/−^ mice were fat fed from 8 to 24 weeks of age. There were no differences in heart/body weight, spleen weight, liver weight, total cholesterol, HDLs (high-density lipoproteins), LDL, triglycerides, blood cell counts, and blood pressure after 16 weeks of HFD (Table III in the Data Supplement). SM22α-hSIRT6/ApoE^−/−^, but not SM22α-hSIRT6^H133Y^/ApoE^−/−^ mice, showed reduced plaque area in the descending aorta (Figure 7A) and aortic root (Figure 7B and 7C) compared with control mice, with similar effects in both males and females. Compared with control mice, plaques of SM22α-hSIRT6^H133Y^/ApoE^−/−^ mice showed decreased cap/plaque and cap/core ratios (Figure 7D and 7E), features of plaque instability most affected by VSMCs, but not core/plaque ratio (Figure 7F). These features of plaque stability were similar in plaques of SM22α-hSIRT6/ApoE^−/−^ mice versus control mice. There was a significant decrease in % α-SMC-actin-positive area in plaques of SM22α-hSIRT6^H133Y^/ApoE^−/−^ mice versus controls (Figure 7G), but no differences in % Mac-3 positive (Figure 7H) or % collagen areas (Figure 7I).

To assess the effect of SIRT6 on plaque cell senescence, SAβG staining was performed on brachiocephalic artery plaques. Plaques of SM22α-hSIRT6/ApoE^−/−^ mice showed reduced % SAβG positive area compared with control mice, which was not observed in SM22α-hSIRT6^H133Y^/ApoE^−/−^ plaques (Figure 7J and Figure VIII in the Data Supplement). As SAβG is not specific for senescence in atherosclerosis in vivo,^29^ we also analyzed aortas for expression of p16 and many senescence-associated secretory phenotype markers. SM22α-hSIRT6/ApoE^−/−^ mice exhibited decreased aortic p16 mRNA expression compared with control mice, which was not seen in SM22α-hSIRT6^H133Y^/ApoE^−/−^ mice (Figure 7K). SM22α-hSIRT6/ApoE^−/−^, but not SM22α-hSIRT6^H133Y^/ApoE^−/−^ mice, also showed reduced mRNA expression of IL-1α and IL-1β, but not IL-6 (Figure 7K). The anti-inflammatory effects of SIRT6 were local to the vessel wall as serum levels of a range of proinflammatory cytokines were similar in all groups (Table III in the Data Supplement).

## Discussion

SIRT6 is an epigenetic regulator of genes linked to aging, inflammation and metabolism. SIRT6 also plays a major role in DNA damage repair signaling,^5^ and its ability to repair double-strand DNA breaks has been directly linked to longevity.^6^ VSMCs in human atherosclerotic plaques are characterized by apoptosis, DNA damage, cell senescence, inflammation, and an altered energy metabolism (reviewed in Grootaert et al^3^). Given its distinct role in each of these processes, SIRT6 is an important target to study in VSMCs and in atherosclerosis.

We report several novel and important findings regarding the role of SIRT6 in VSMCs in atherosclerosis, namely: (1) SIRT6 protein (but not mRNA) expression is reduced in VSMCs in human and mouse atherosclerotic plaques; (2) the ubiquitin ligase CHIP is a crucial regulator of SIRT6 stability by binding to SIRT6 and preventing SIRT6 proteasomal degradation; (3) CHIP expression is reduced in VSMCs in human and mouse atherosclerosis and correlates with SIRT6 expression; (4) both CHIP and SIRT6 are reduced in human VSMCs by the free fatty acid palmitate, and in mouse aorta after high-fat feeding; (5) p38 and JNK inhibition prevent palmitate-induced loss of CHIP and SIRT6 protein; (6) loss of endogenous SIRT6 or loss of SIRT6 deacetylase activity induces VSMC senescence; (7) SIRT6 binds to telomeres in VSMCs, regulates telomeric H3K9 deacetylation and protects against telomere damage, activities that depend upon its deacetylation activity; (8) SIRT6 partially reverses the senescence-associated inflammatory and metabolic phenotype of VSMCs, but not in early passage cells; (9) VSMC-specific overexpression of SIRT6 inhibits atherogenesis and reduces tissue markers of cell senescence and inflammation in vivo, dependent upon its deacetylase activity; and (10) expression of SIRT6 lacking deacetylase activity in VSMCs promotes VSMC features associated with plaque instability.

We demonstrate that SIRT6 protein, but not mRNA, expression is reduced in VSMCs in mouse and human atherosclerotic plaques, in VSMCs cultured from human plaques versus VSMCs from normal vessels, and in hVSMCs undergoing replicative and palmitate-induced senescence. Palmitate is an important pathological mediator of vascular disease by inducing DNA damage. However, DNA damage induced by palmitate does not occur before SIRT6 downregulation, suggesting other regulatory mechanisms are involved. We found that SIRT6 protein stability in hVSMCs is positively regulated by the ubiquitin ligase CHIP, but CHIP expression is reduced in VSMCs in human atherosclerotic plaques, in VSMCs cultured from human plaques, and in mouse plaques and aortas after high-fat feeding. CHIP interacts with endogenous SIRT6 and prevents SIRT6 proteasomal degradation, while loss of CHIP accelerates SIRT6 degradation and reduces SIRT6 half-life by 56%. Both CHIP and SIRT6 expression are reduced by palmitate in a CD36-independent manner, and CHIP-SIRT6 interaction is reduced by palmitate, but SIRT6 protein stability is recovered when CHIP is overexpressed. Palmitate activates p38 and JNK signaling, and p38 inhibition can fully recover CHIP and SIRT6 protein expression in the presence of palmitate. Loss of SIRT6 expression is not observed upon exposure to other lipid-species suggesting that the downregulation of SIRT6 seen in VSMCs in atherosclerosis may be a more specific result of fatty acids rather than a nonspecific effect of exposure to lipids. To our knowledge, this is the first study of CHIP in atherosclerosis and highlights the importance of pathways that regulate stability of epigenetic controllers of aging like SIRT6. It is also possible that SIRT6 stability is regulated by other post-translation mechanisms, such as phosphorylation, although SIRT6 phosphorylation by either palmitate^30^ or JNK^31^ promotes its stability rather than its degradation. Our findings suggest a specific mechanism for SIRT6 downregulation in atherosclerosis, through fatty acid-induced reduction in CHIP and increased SIRT6 degradation.

VSMCs from human atherosclerotic plaques undergo senescence and VSMC senescence promotes atherosclerosis and plaque instability,^15^ while removal of senescent cells can reduce atherosclerosis.^32^ However, while plaque VSMCs, human VSMCs undergoing replicative senescence or palmitate-induced senescence all showed reduced SIRT6 protein expression, whether downregulation of SIRT6 is a cause or merely a consequence of VSMC senescence was unclear. We show that downregulation of SIRT6 upon palmitate is persistent and occurs before the onset of palmitate-induced DNA damage and senescence, and DNA damage induced by doxorubicin or telomere disruption does not reduce SIRT6; thus DNA damage is a consequence not the cause of reduced SIRT6 in VSMCs. Similarly, overexpression of SIRT6 delays replicative senescence in human and mouse VSMC cultures, while a catalytically inactive SIRT6^H133Y^ and shRNA-mediated knockdown of SIRT6 as loss-of-function approaches induce VSMC senescence. Our study indicates that endogenous levels of SIRT6 are a critical regulator of VSMC senescence which depend upon its catalytic activity, and suggest that loss of SIRT6 activity is an important factor in VSMC senescence in atherosclerosis.

SIRT6 exerts multiple cytoprotective effects via the regulation of cell death,^8^ DNA damage,^5^ cell metabolism,^11,12^ and inflammation^9^ that could account for its ability to prevent cell senescence. In human VSMCs, loss of SIRT6 protein or its activity had no effect on apoptosis or basal global DNA damage. Instead, we show that SIRT6 plays a crucial role in maintaining telomere integrity and preventing telomere damage. Furthermore, SIRT6 is upregulated in hVSMCs exhibiting persistent telomere damage due to a defect in the TRF2 protein, likely acting as a DNA damage sensor in an attempt to limit telomere damage. In basal conditions, VSMC SIRT6 is able to bind to telomeres and to deacetylate telomeric H3K9, while loss of SIRT6 leads to H3K9 hyperacetylation of telomeres. SIRT6-mediated histone deacetylation is required for the stable association of telomere-processing factors to prevent replication-associated telomere dysfunction and senescence.^7^ We show that loss of SIRT6 enzymatic activity (through SIRT6^H133Y^) impedes its telomere binding, resulting in hyperacetylation of telomeric DNA. Moreover, SIRT6 depletion or inactivity led to telomere damage as evidenced by increased telomere enrichment of 53BP1, known to promote nonhomologous end joining of dysfunctional telomeres,^21^ but without changes in expression of shelterin proteins TRF1 and TRF2. Overall, our data imply that SIRT6’s role in telomere maintenance is essential for regulating VSMC lifespan, and failure to preserve telomere integrity by SIRT6 regulation of telomere histone deacetylation triggers telomere damage and senescence. The absence of SIRT6 as observed in plaque VSMCs will further compromise the cell’s ability to sense and limit telomere damage, creating a vicious circle.

Similar to plaque VSMCs,^22,23^ senescent cells acquire a glycolytic^24,25^ and highly inflammatory state and may promote atherosclerosis via their senescence-associated secretory phenotype.^26^ In some cell types, SIRT6 can epigenetically control glucose^11^ and fatty acid^12,13^ metabolism, and NF-κB-dependent inflammation.^9^ In contrast, we find that SIRT6 does not transcriptionally regulate glycolysis, FAO and proinflammatory cytokine expression in proliferating hVSMCs, and NF-κB is not activated in senescent SIRT6-depleted hVSMCs. However, VSMCs lacking SIRT6 acquired a proinflammatory and glycolytic phenotype at senescence, suggesting that SIRT6-regulated changes in metabolism and inflammation do not directly promote senescence but maintained SIRT6 expression can delay the appearance of characteristics of the senescent phenotype. Thus, by inhibiting VSMC senescence, SIRT6 could also prevent the deleterious inflammatory and metabolic phenotype of senescence that could further promote atherosclerosis.

To investigate the effect of SIRT6 in VSMCs in vivo, we generated 2 new transgenic mouse models that overexpress VSMC-specific hSIRT6 or hSIRT6^H133Y^. VSMC SIRT6 reduces atherosclerosis and markers of plaque cell senescence and preserves features of plaque stability in ApoE^−/−^ mice, which is lost when VSMCs express SIRT6^H133Y^. Moreover, VSMC SIRT6 mice showed reduced aortic expression of p16 and senescence-associated cytokines compared with controls, without changes in serum cytokines, suggesting that a reduced local senescence-associated secretory phenotype might reduce plaque burden in these mice. In addition to VSMC senescence, other pathways regulated by SIRT6 could also be involved in regulating atherogenesis, including Nrf2 (nuclear factor erythroid 2–related factor 2)-mediated antioxidant pathways,^33^ PCSk9 (proprotein convertase subtilisin/kexin type 9)-dependent lipid metabolism,^34^ reversed cholesterol transport,^35^ and vascular nitric oxide bioavailability.^8^ However, we observed no difference in blood pressure, serum lipids, and Mac-3 expressing plaque cells between experimental mouse groups, most likely related to SIRT6 expression only in arterial vascular VSMCs. Our data are consistent with previous observations showing increased atherosclerosis in ApoE^−/−^ mice with whole-body knockdown of SIRT6^36,37^ but emphasize the atheroprotective and antisenescence effect of VSMC SIRT6 specifically, and the importance of its deacetylase function.

Our study has some limitations. First, expression of the transgene (SIRT6 or SIRT6^H133Y^) under the minimal SM22α promoter could potentially affect arterial VSMC development; however, there were no gross or histological abnormalities seen in arteries before high-fat feeding and expression of SIRT6^H133Y^ controls for possible off-target effects. Second, VSMC promoters (such as SM22α) can also be expressed by myeloid cells that differentiate and accumulate in plaques.^38^ However, we have previously shown that the minimal SM22α promoter is not expressed in bone marrow, peripheral blood, or spleen.^39^ Third, SIRT6 expression in VSMCs in human plaques might be different from normal aortas due to risk factors, such as diabetes or hypertension; however, cultured VSMCs showed markedly reduced SIRT6 even when isolated from the patient. Finally, we only studied SIRT6’s deacetylase function. SIRT6 also has a long-chain deacylase activity (reviewed in Chang et al^40^), such that its nonhistone targets may have distinct roles in atherosclerosis.

**Figure 8.**
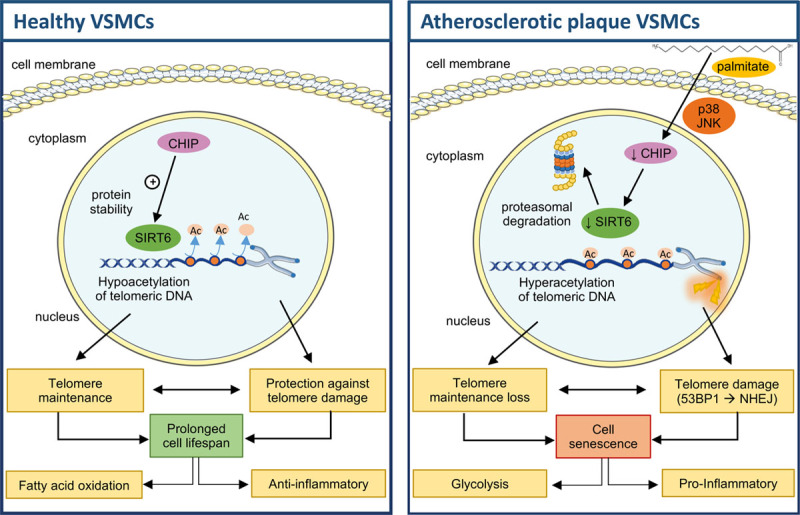
**Schematic overview of SIRT6 (Sirtuin 6) regulation in vascular smooth muscle cells (VSMCs) in atherosclerosis.** In normal healthy aortic VSMCs, the ubiquitin ligase CHIP (C terminus of HSC70-interacting protein) binds to SIRT6 and promotes its stability. SIRT6 deacetylates histone 3 at telomeric DNA, preserves telomere integrity and protects against telomere damage, prolonging VSMC lifespan. In atherosclerosis, CHIP expression is reduced upon exposure to free fatty acids such as palmitate in a p38 and JNK (c-Jun N-terminal kinase)-dependent manner. As a result, SIRT6 is no longer protected from proteasomal degradation. Loss of SIRT6 binding (or its activity) leads to hyperacetylation of telomere histones and 53BP1 (p53 binding protein 1; NHEJ)-mediated telomere damage, which impairs telomere integrity and promotes senescence. Senescent VSMCs are characterized by a metabolic shift from fatty acid oxidation to glycolysis and increased expression of proinflammatory markers. JNK indicates c-Jun N-terminal kinase; and NHEJ, nonhomologous end joining.

Our data support the following model for how VSMC SIRT6 regulates atherosclerosis (Figure 8). Plasma free fatty acids such as palmitate accumulate in the arterial wall. Palmitate uptake by VSMCs activates p38 and JNK signaling and reduces expression of the ubiquitin ligase CHIP, hence, SIRT6 is no longer protected by CHIP from proteasomal degradation. Loss of SIRT6 binding (or its activity) leads to hyperacetylation of telomere histones and telomere damage, resulting in loss of telomere integrity, triggering VSMC senescence. Senescent VSMCs acquire a glycolytic and proinflammatory state that can also promote atherosclerosis, and which can be prevented by restoring SIRT6 levels.

In summary, we describe a novel, important and unrecognized role for SIRT6 in VSMC senescence and atherosclerosis, and identify the ubiquitin ligase CHIP as a critical regulator of SIRT6 stability, whose expression is also reduced in VSMCs in atherosclerosis. SIRT6’s deacetylase activity is crucial for its antisenescence and telomere maintenance function. Restoring SIRT6 levels in VSMCs protects against atherosclerosis, plaque cell senescence, and inflammation and preserves features of plaque stability, revealing a therapeutic potential of SIRT6 in atherosclerosis.

## Sources of Funding

This work was funded by British Heart Foundation (BHF) grants RG/13/14/30314, RG/20/2/34763, PG/6/24/32090, PG/16/11/32021, PG/13/14/30314, and CH/2000003, the National Institute of Health Research (NIHR) Cambridge Biomedical Research Centre, NIHR Senior Investigator NF-SI-0616-10036, and the BHF Centre for Research Excellence RE/18/1/34212.

## Disclosures

None.

## Supplemental Materials

Expanded Materials & Methods

Online Figures I–VIII

Online Tables I–III

## Supplementary Material



## Novelty and Significance

### What Is Known?

- Senescent vascular smooth muscle cells (VSMCs) accumulate in human atherosclerotic plaques and promote atherosclerosis in preclinical models.
- VSMC senescence is triggered by oxidative stress, (telomere) DNA damage, and replicative exhaustion.
- SIRT6 (Sirtuin 6) is a deacetylase protein that has multiple effects on cell proliferation, death, inflammation, and metabolism, all of which are altered in cell senescence and atherosclerosis.
