# Supplementary figures and images for: SIRT6 Protects Smooth Muscle Cells From Senescence and Reduces Atherosclerosis
Source: Circ Res. 2020 Dec 23;128(4):474–91. doi: 10.1161/CIRCRESAHA.120.318353 (PMC7899748; doi:10.1161/CIRCRESAHA.120.318353)

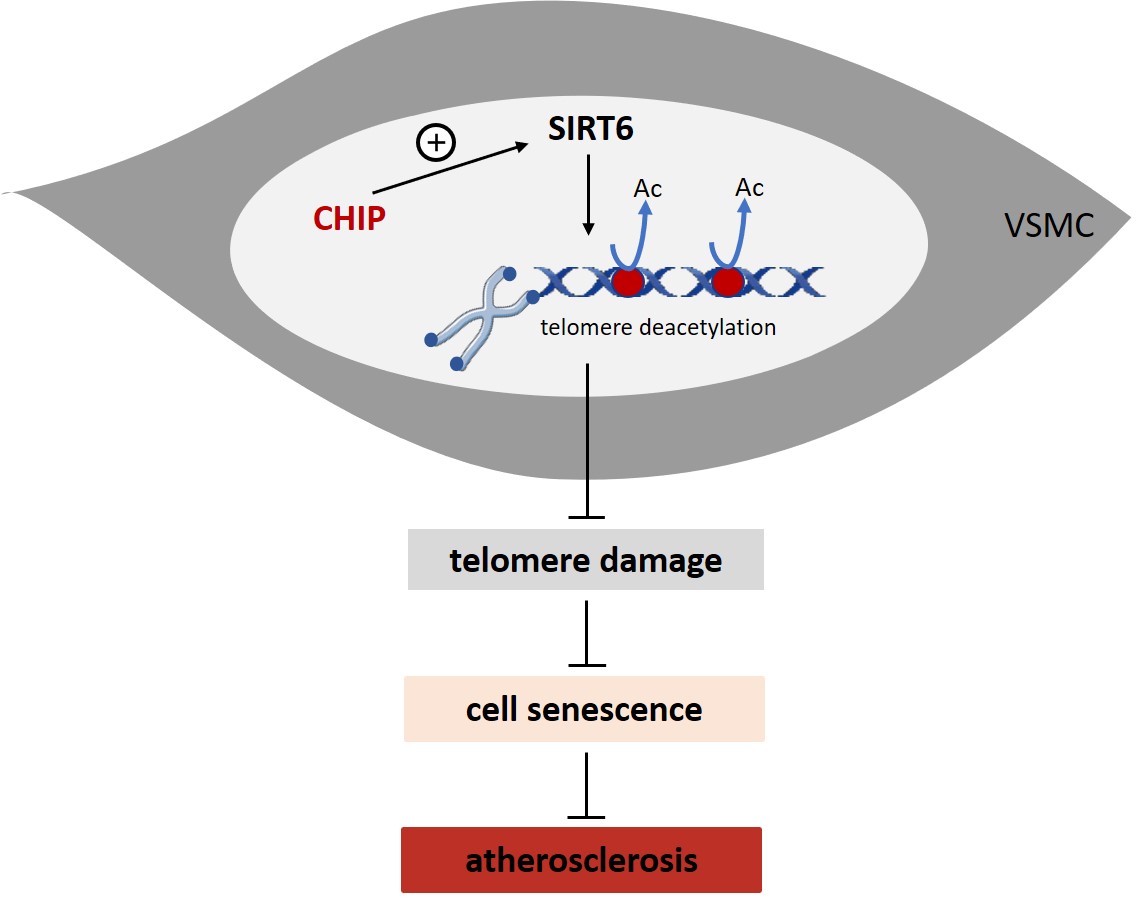

Supplement: Supplementary file 1 [file res-128-474-s001.jpg]
