# Supplementary material for: SIRT6 Protects Smooth Muscle Cells From Senescence and Reduces Atherosclerosis
Source: Circ Res. 2020 Dec 23;128(4):474–91. doi: 10.1161/CIRCRESAHA.120.318353 (PMC7899748; doi:10.1161/CIRCRESAHA.120.318353)

Full unedited gel for Fig. 1D

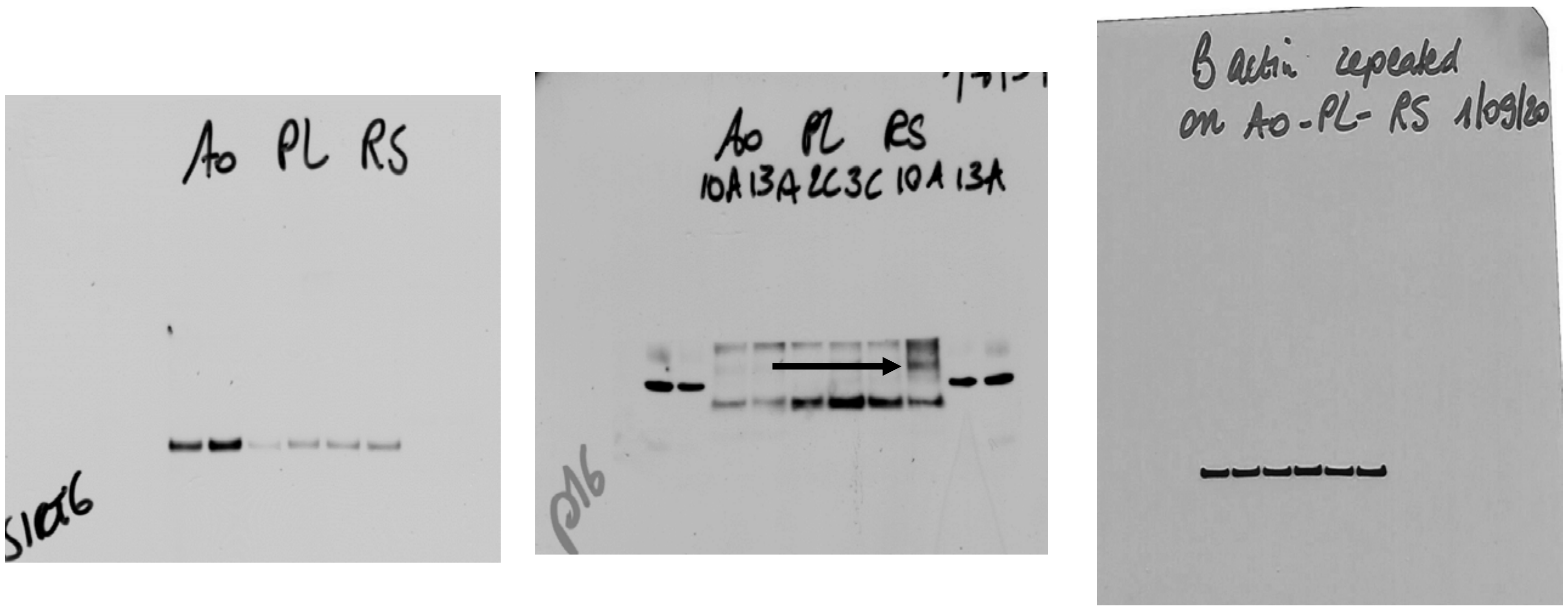

Full unedited gel for Fig. 1E

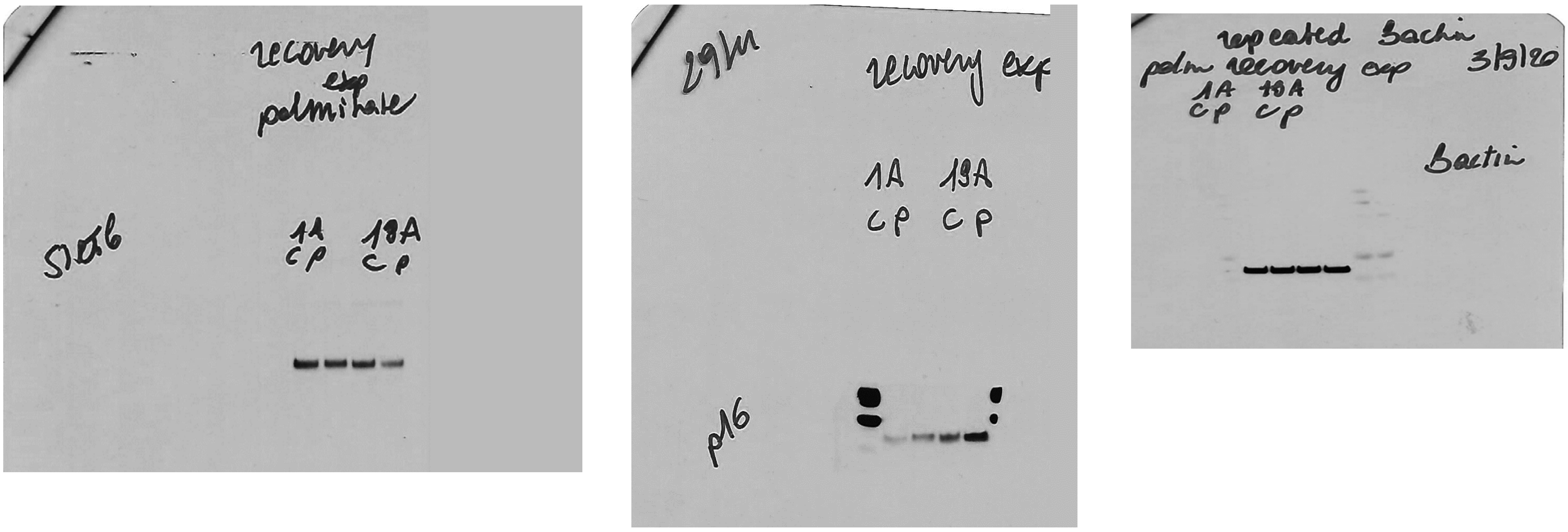

Full unedited gel for Fig. 2A

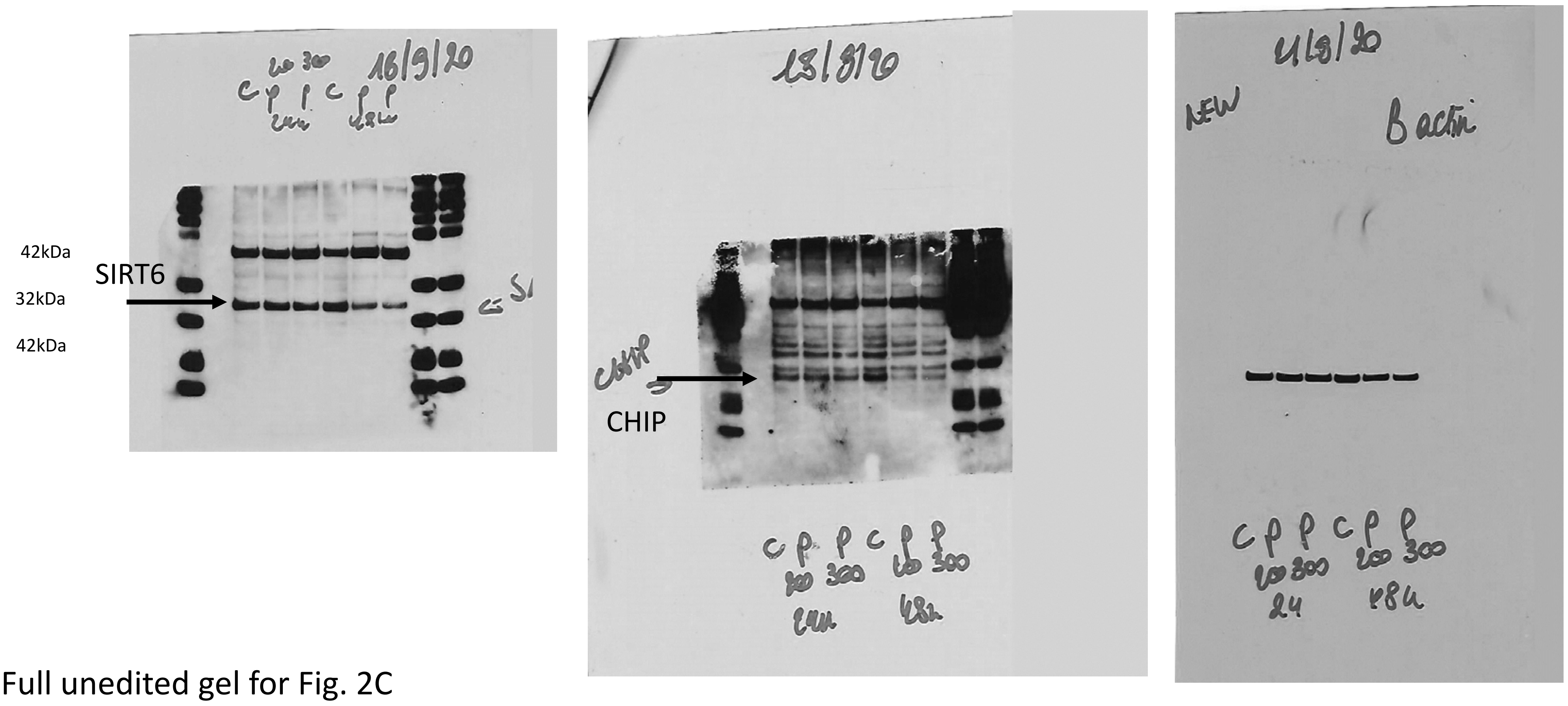

Full unedited gel for Fig. 2C

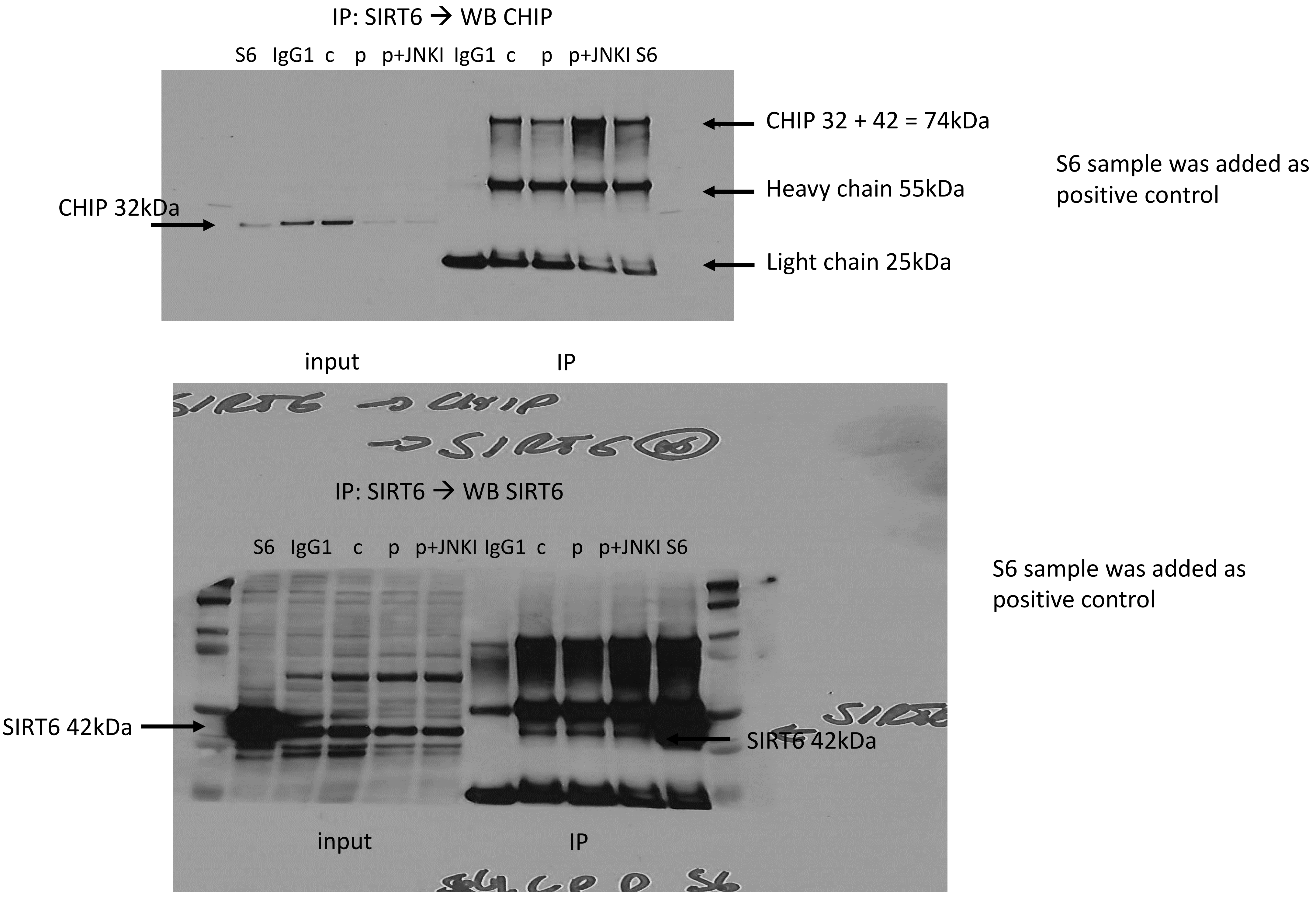

Full unedited gel for Fig. 2D

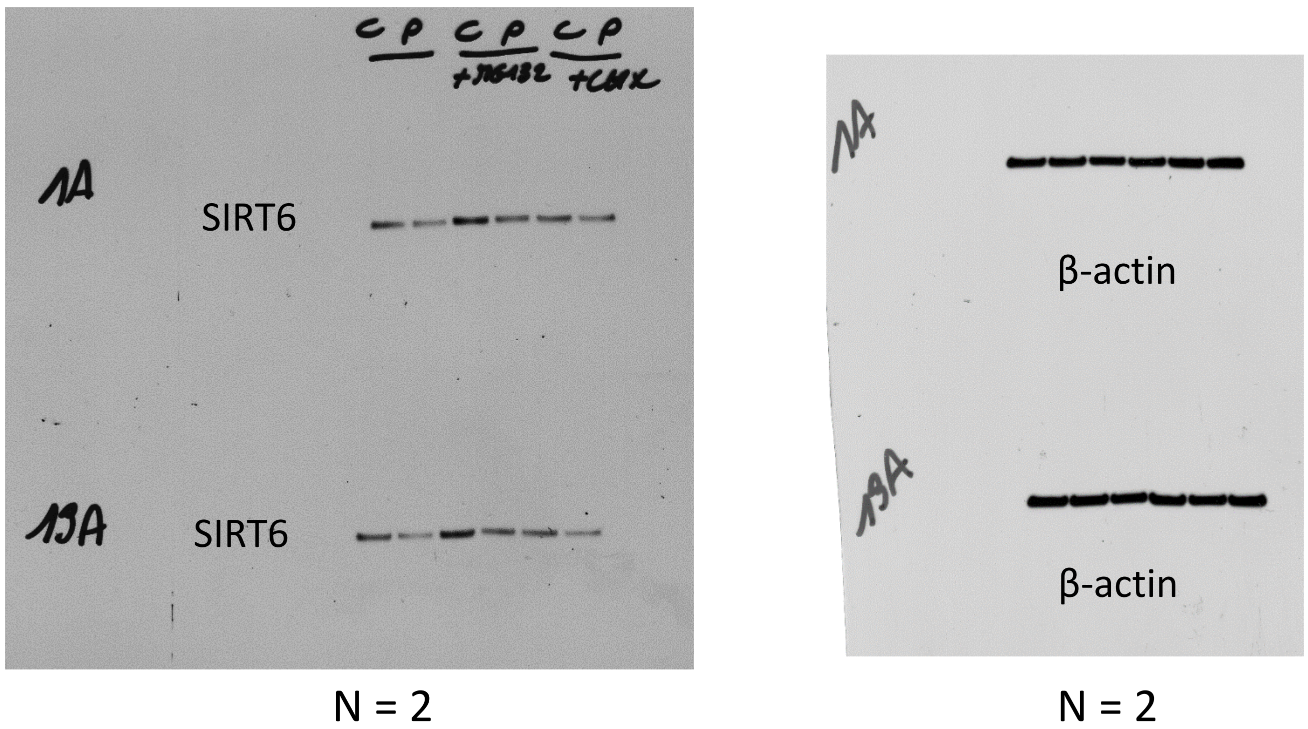

Full unedited gel for Fig. 2E

IP: SIRT6 → WB ubiquitin

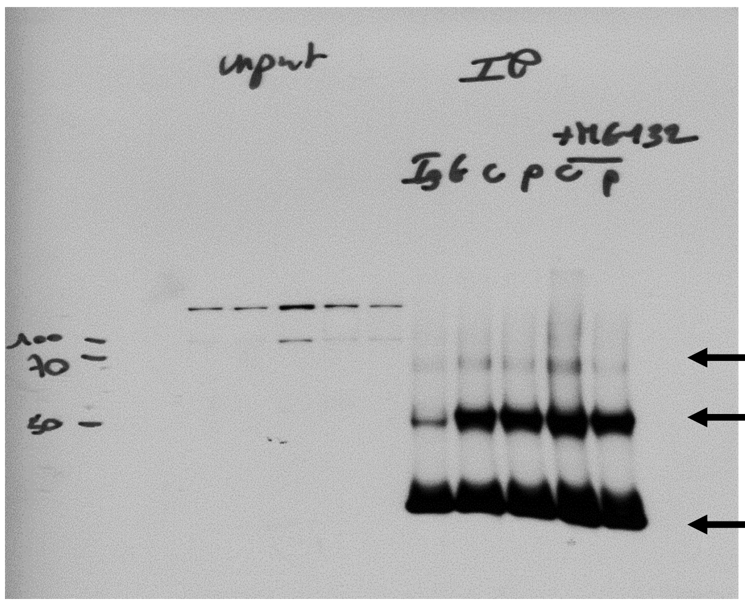

ubiquitin

Heavy chain 55 kDa

Light chain 25 kDa

input IP

IP: SIRT6 → WB SIRT6

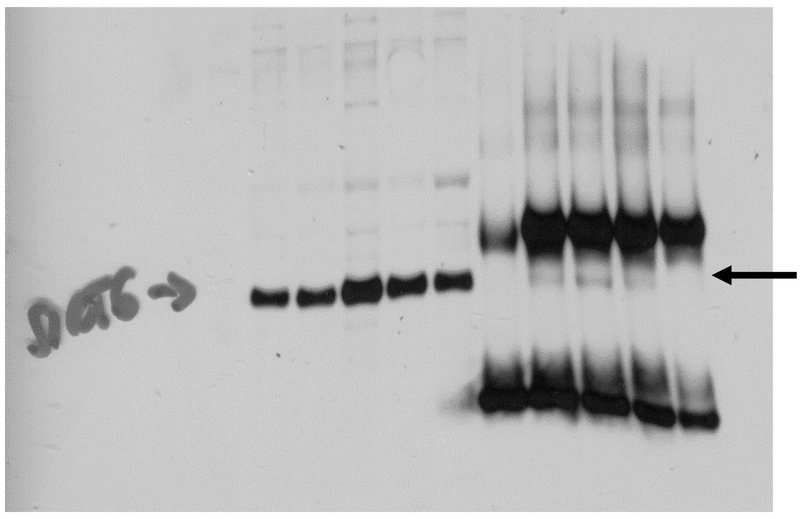

SIRT6

input IP

Full unedited gel for Fig. 2F

N=2 on one gel

CHIP 32kDa

SIRT6 42kDa

B-actin 42kDa

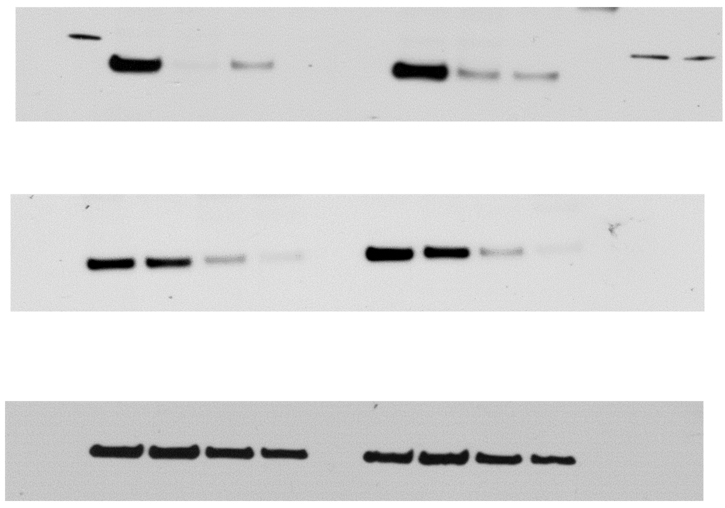

Full unedited gel for Fig. 2G

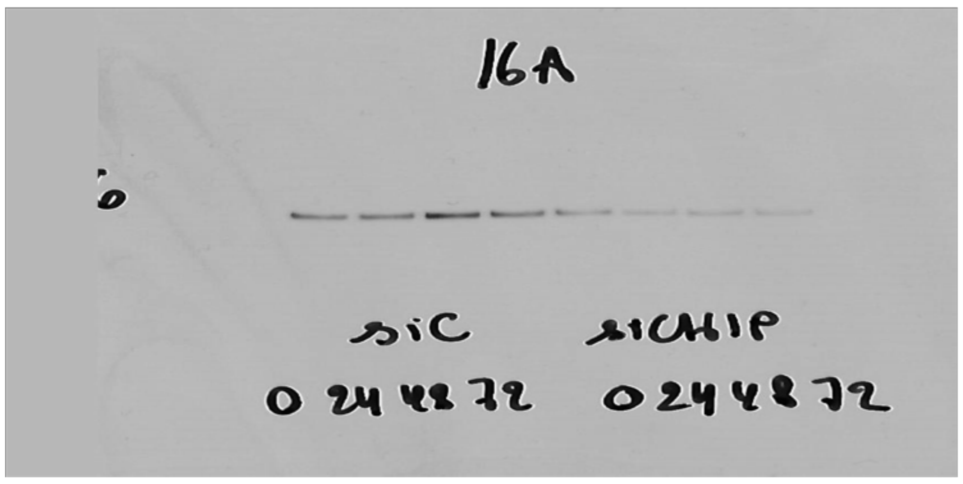

SIRT6 42kDa

Full unedited gel for Fig. 2H

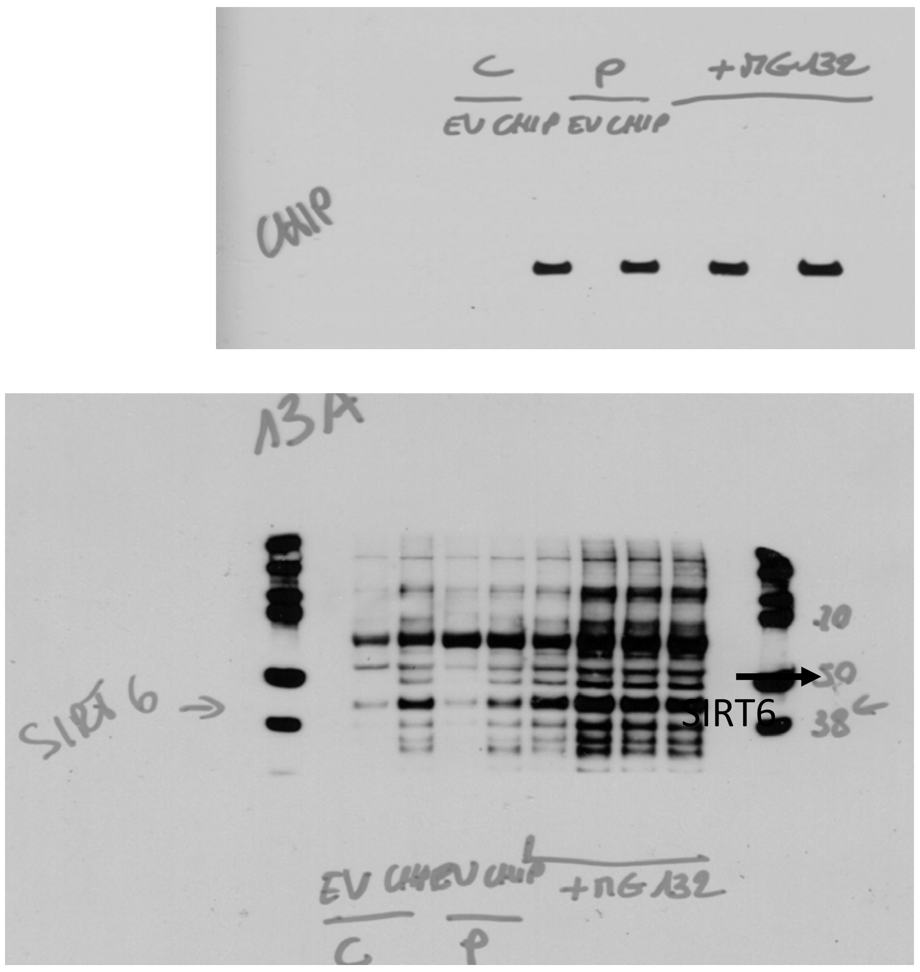

Myc

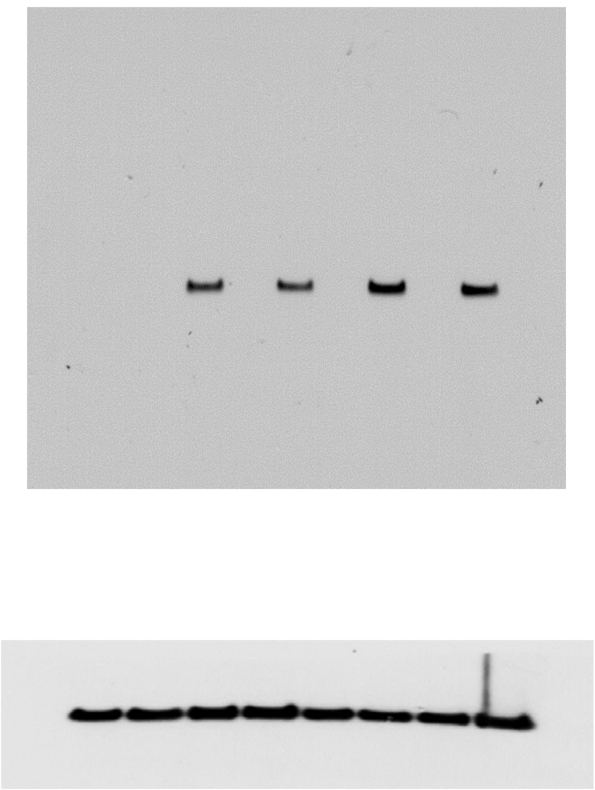

B-actin

Full unedited gel for Fig. 2J

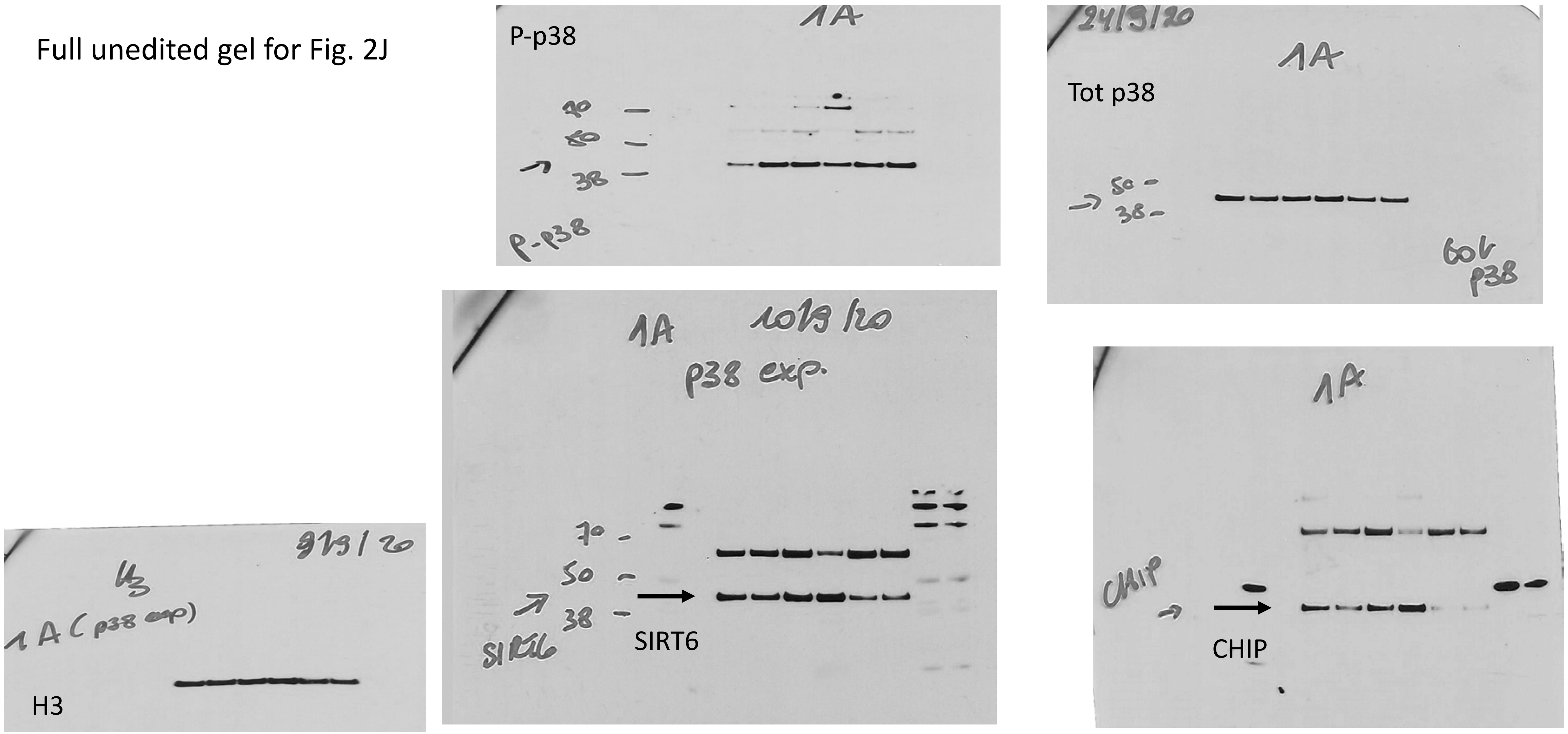

Full unedited gel for Fig. 2K

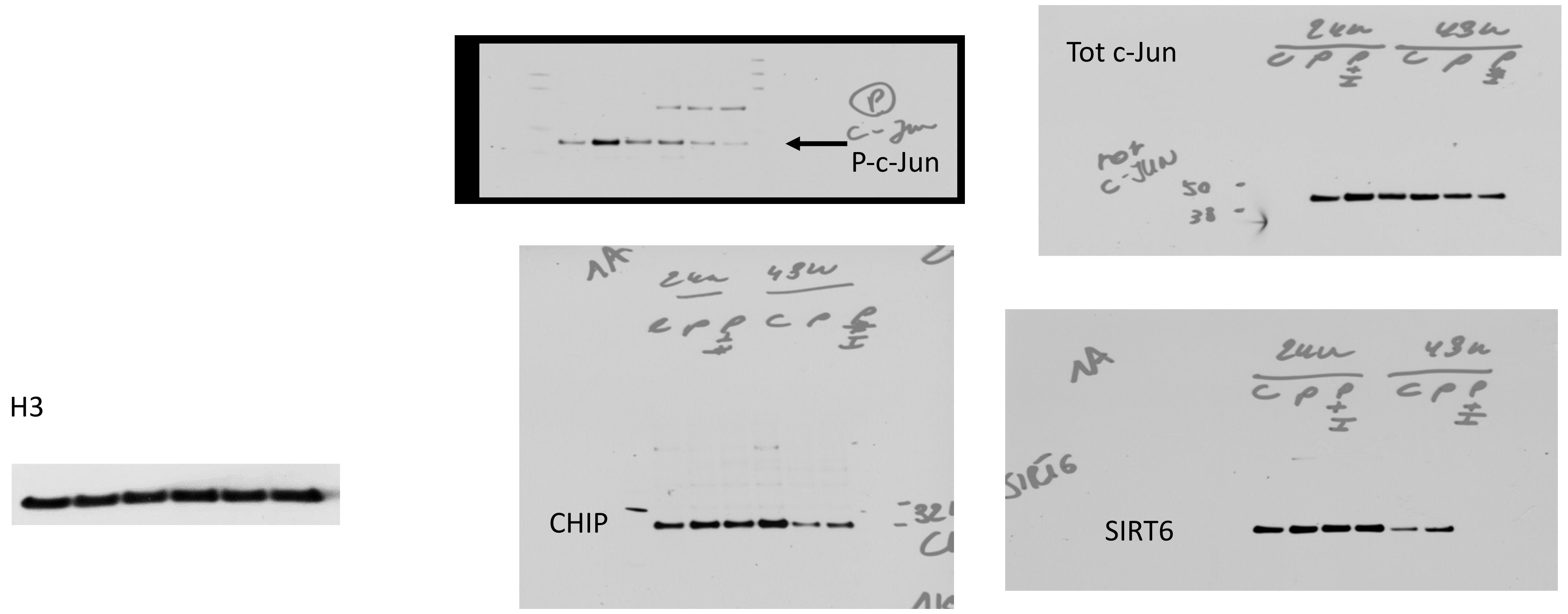

Full unedited gel for Fig. 3A

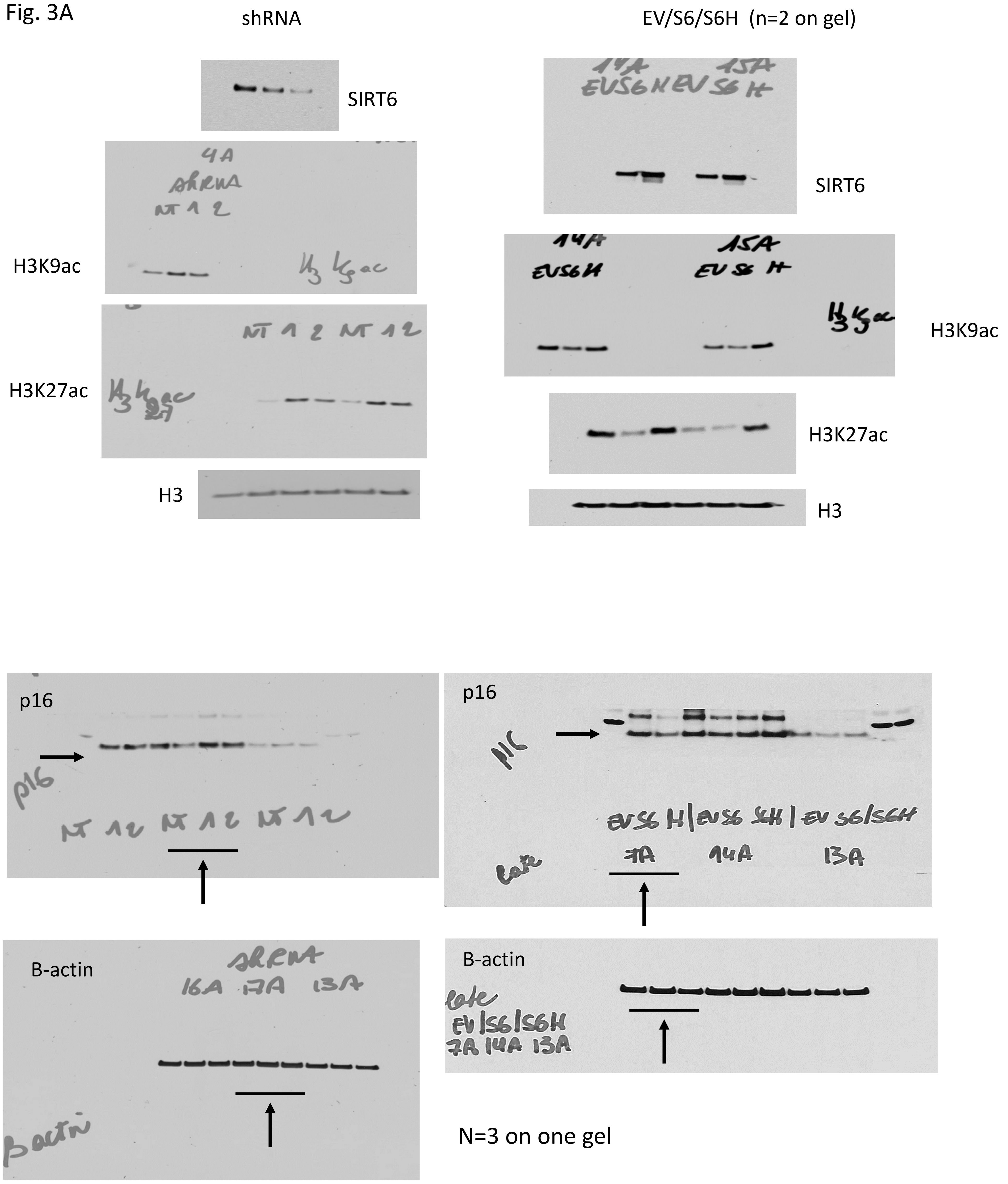

Full unedited gel for Fig. 5A

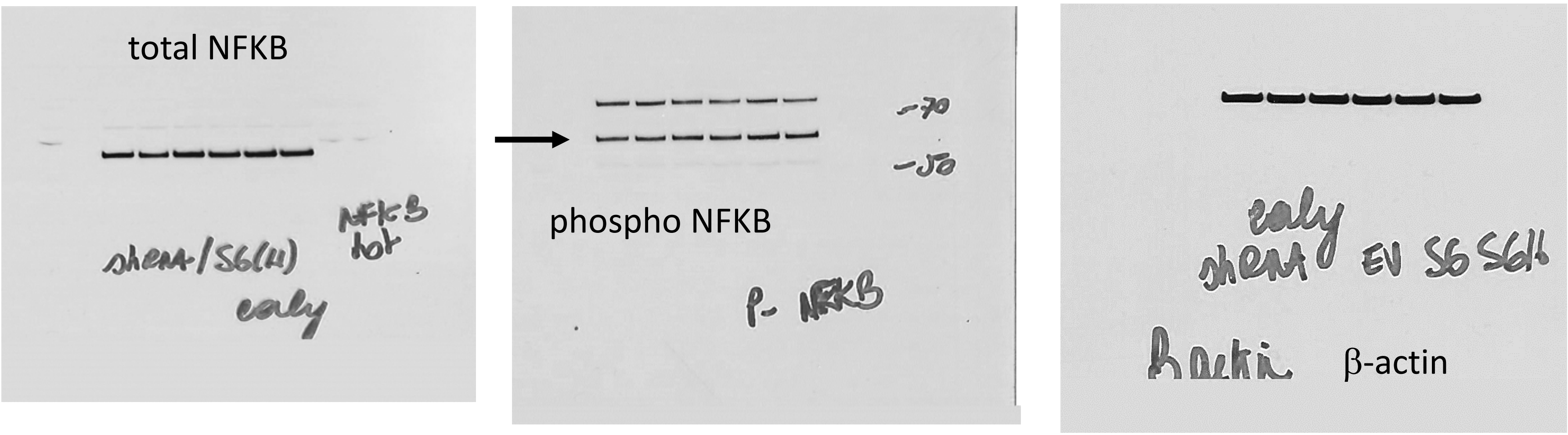

shNT/sh#1/sh#2/EV/S6/S6H

Full unedited gel for Fig. 5C

Total NFκB

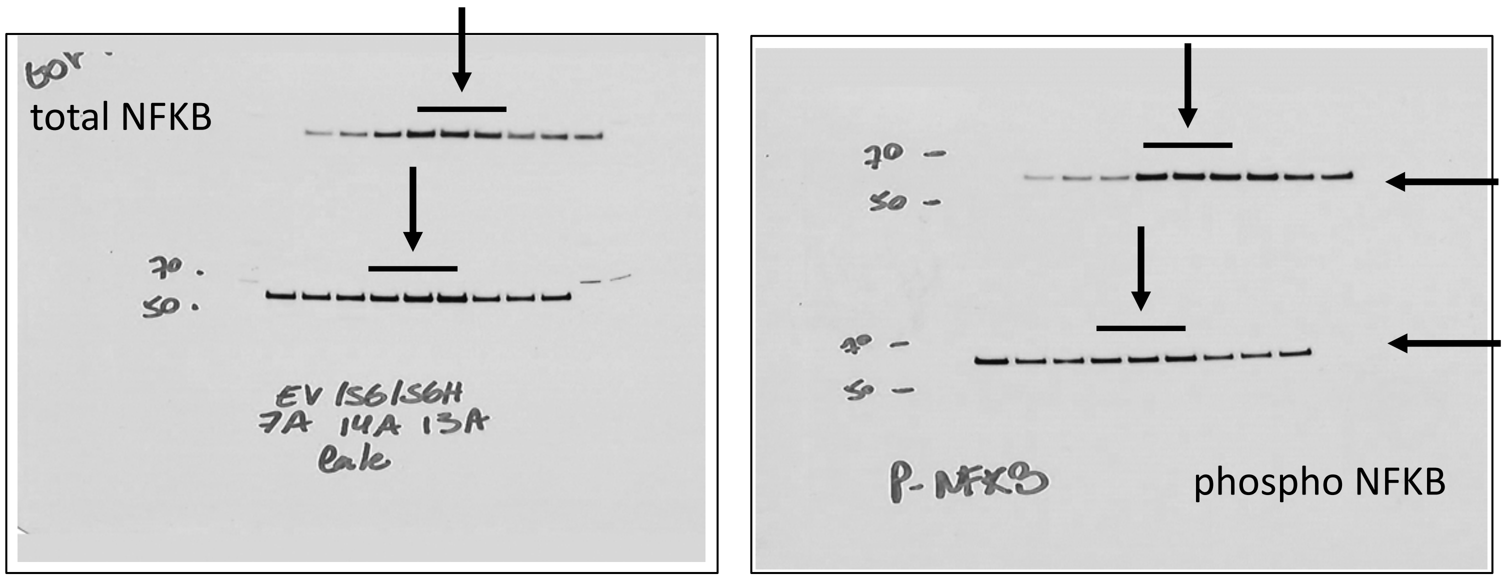

shRNA

EV/S6/S6H

N=3 on one gel

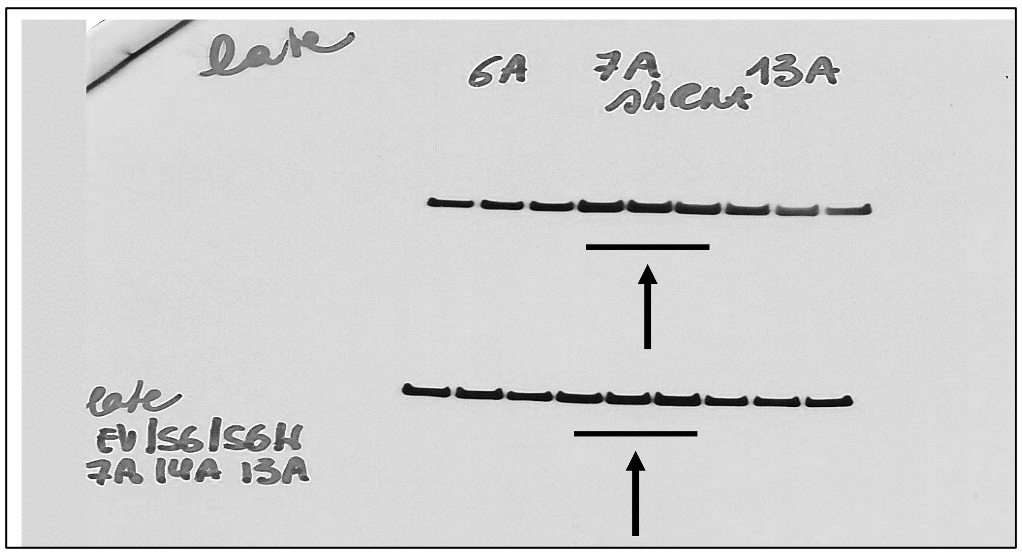

β-actin

Full unedited gel for Fig. 6A

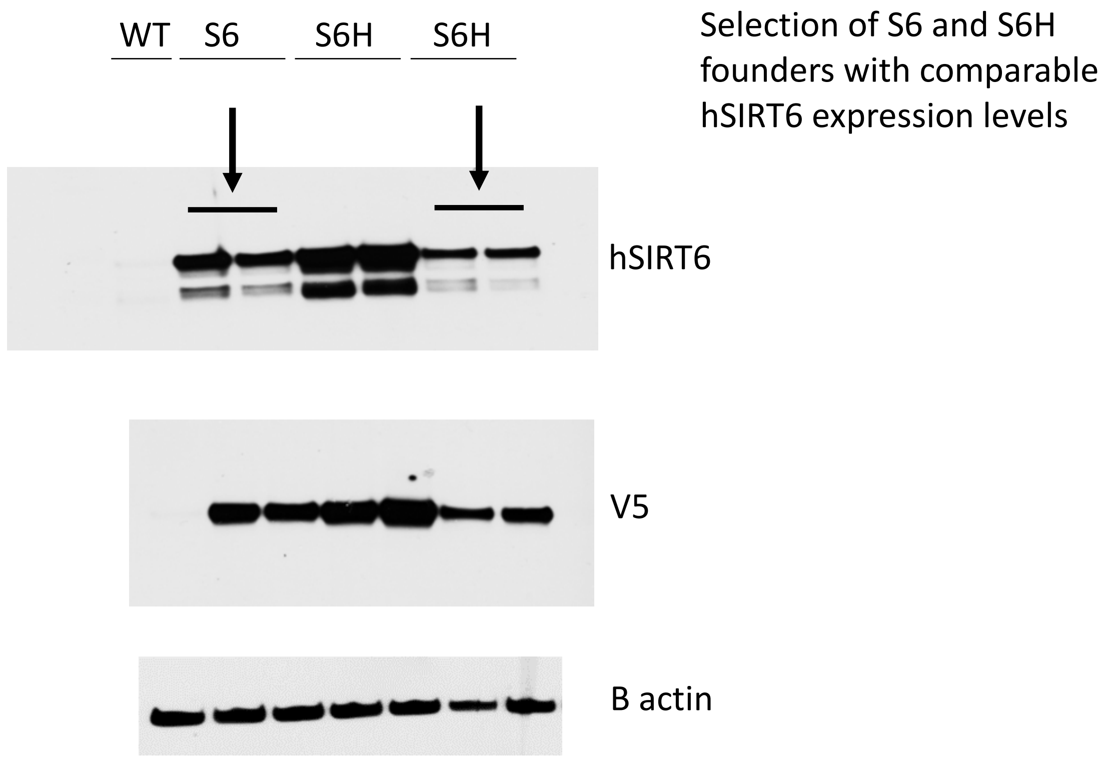

Full unedited gel for Fig. 6C

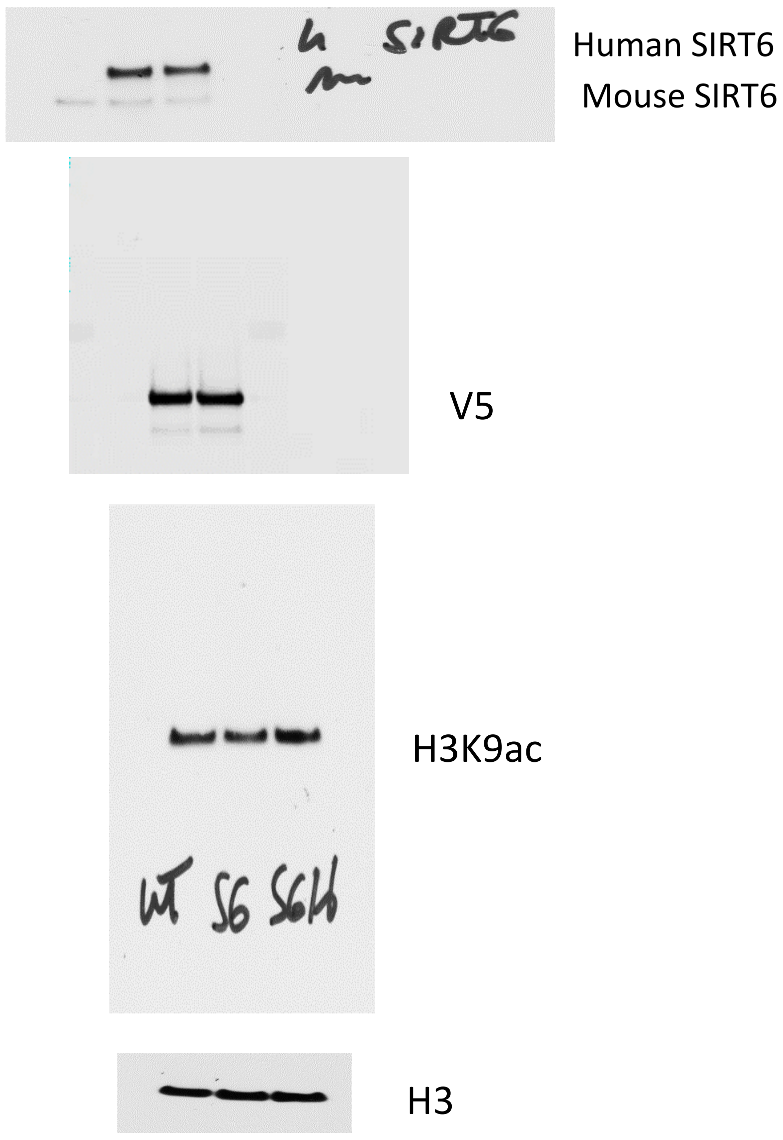

Full unedited gel for Suppl Fig. I C

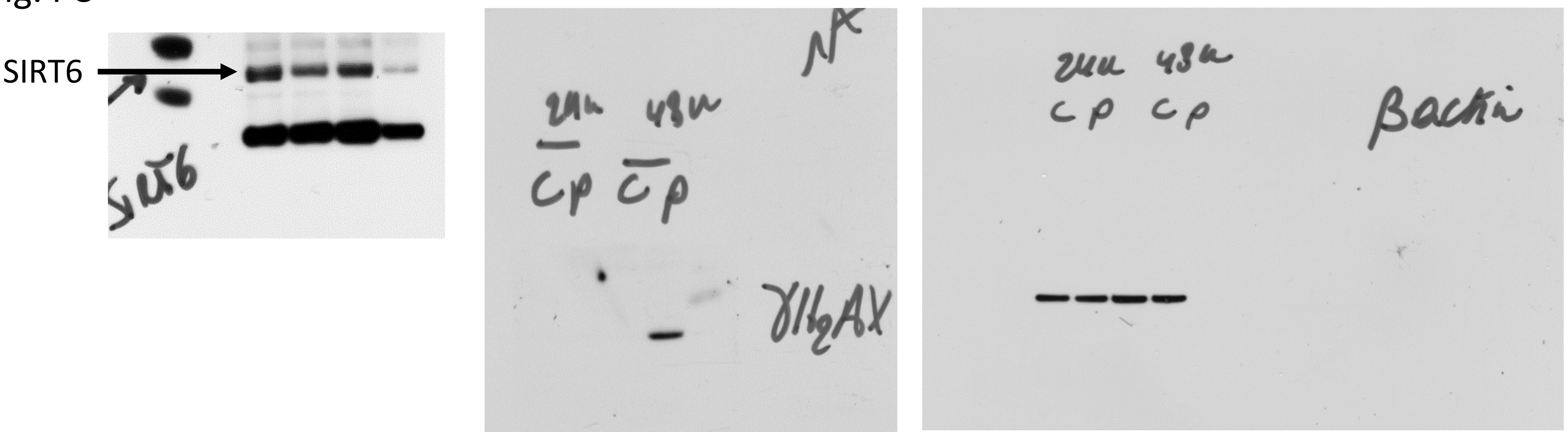

Full unedited gel for Suppl Fig. I D

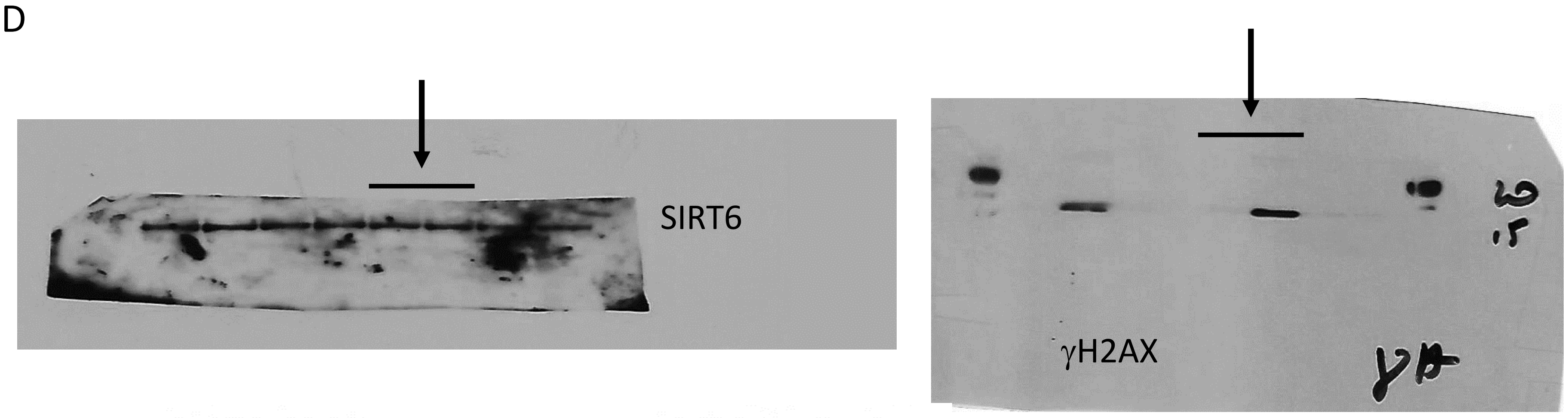

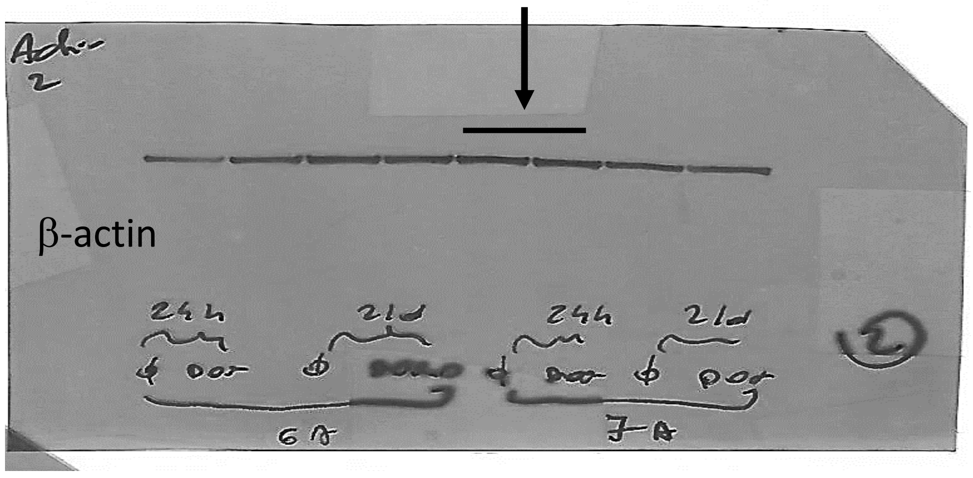

N=2 for DOXO 24h (used for paper)
N=2 for DOXO 21d
All on one gel

Full unedited gel for Suppl Fig. I E

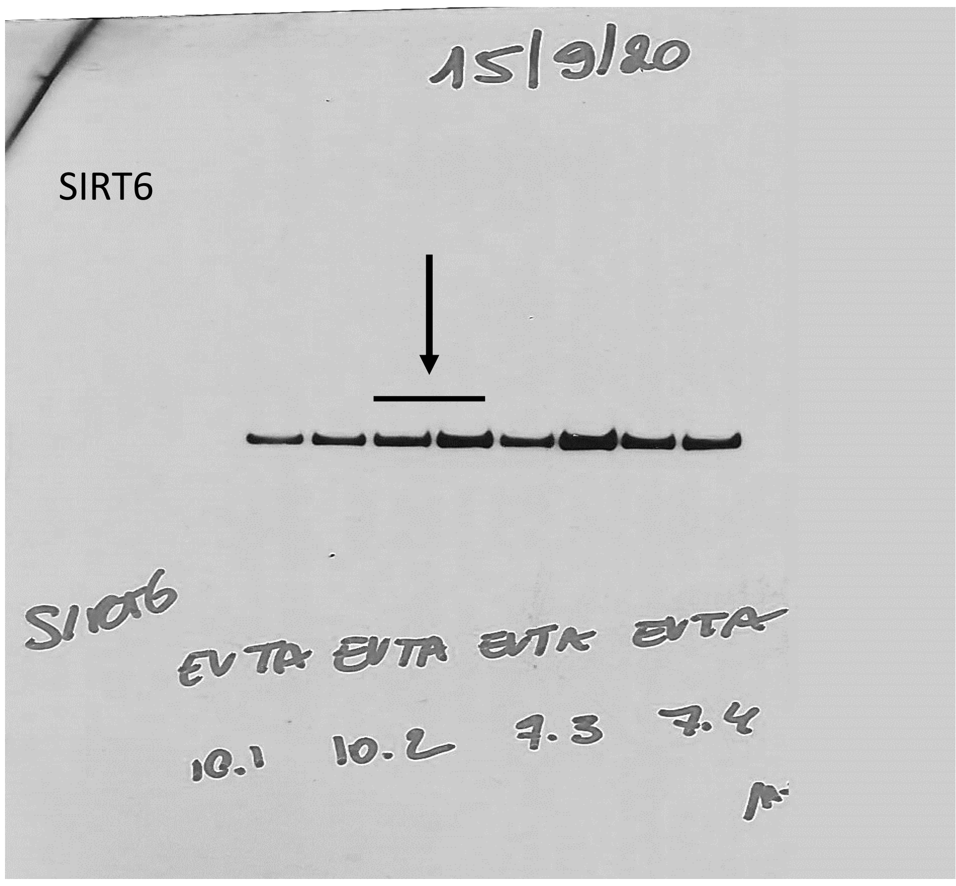

N=4 on one gel

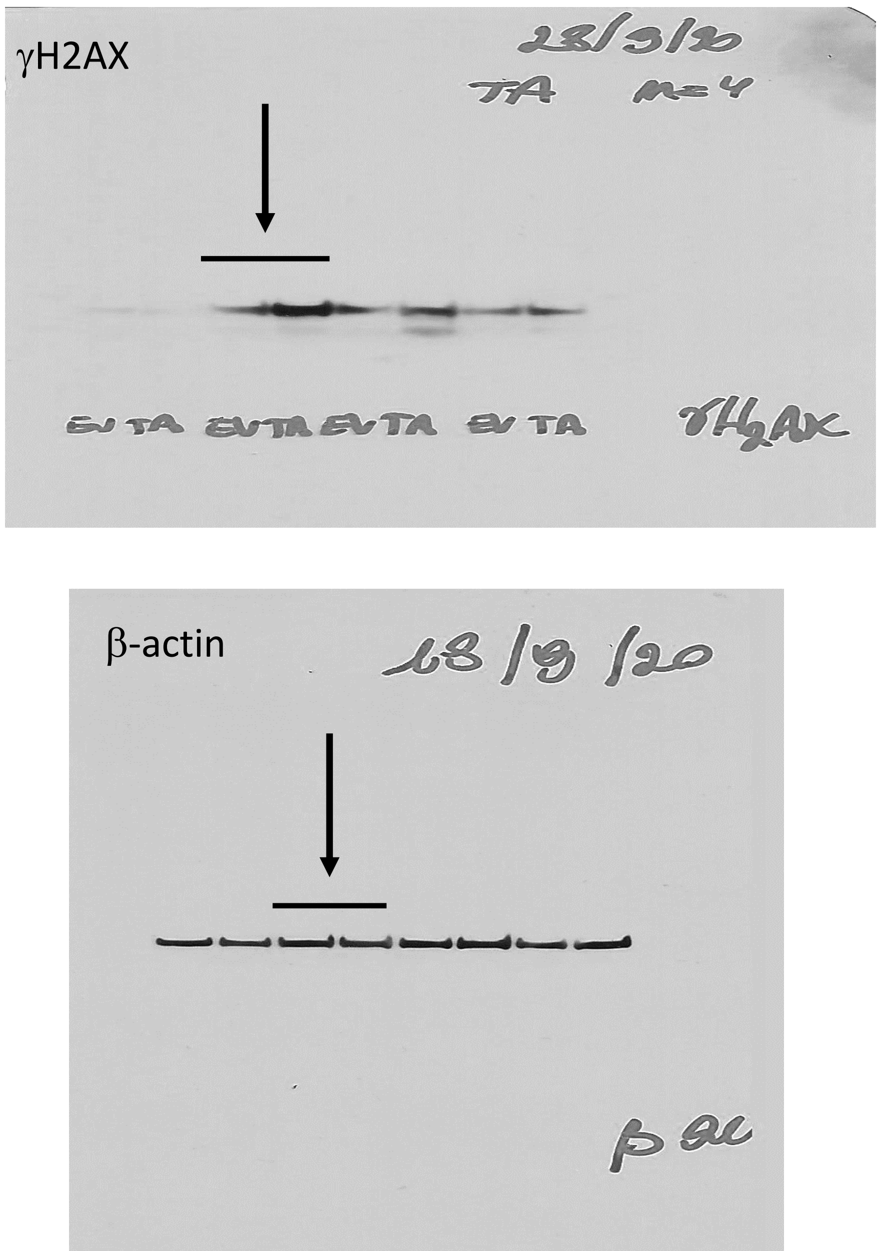

Full unedited gel for Suppl Fig. II A

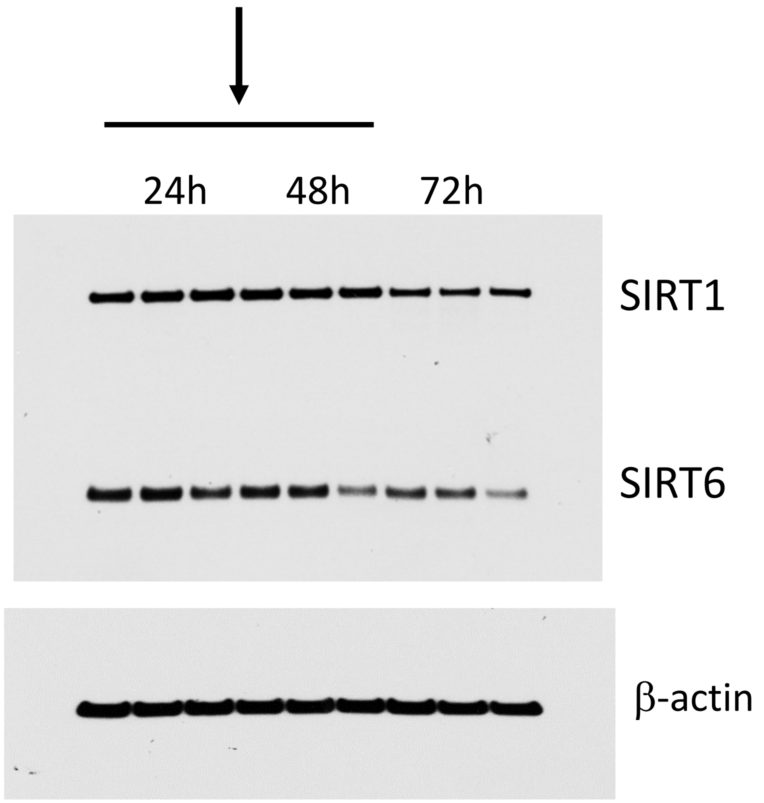

Full unedited gel for Suppl Fig. II B

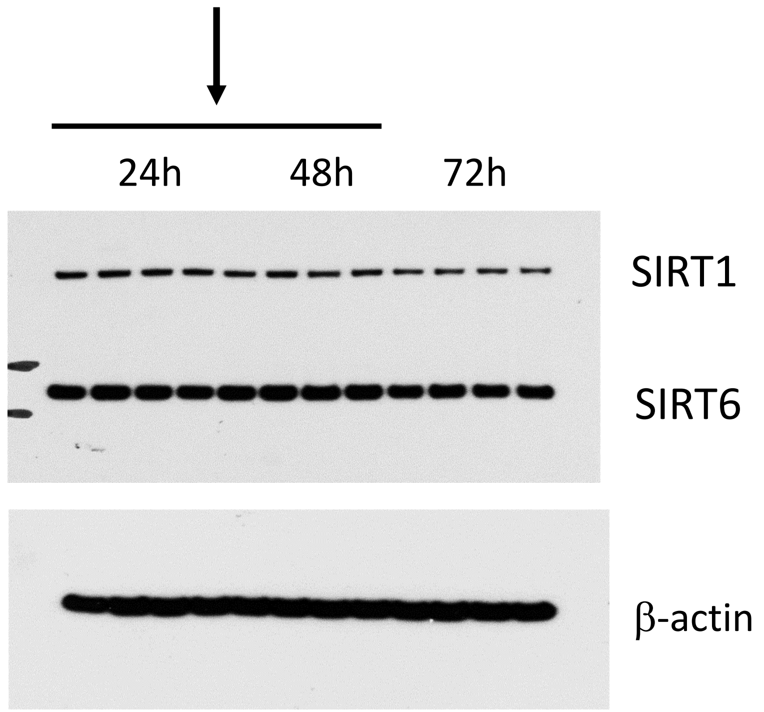

Full unedited gel for Suppl Fig. II C

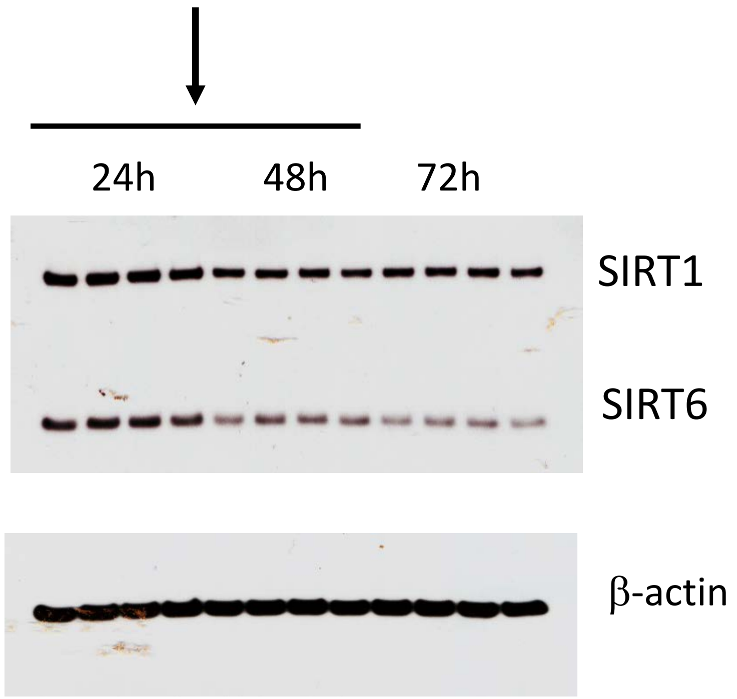

Full unedited gel for Suppl Fig. II E

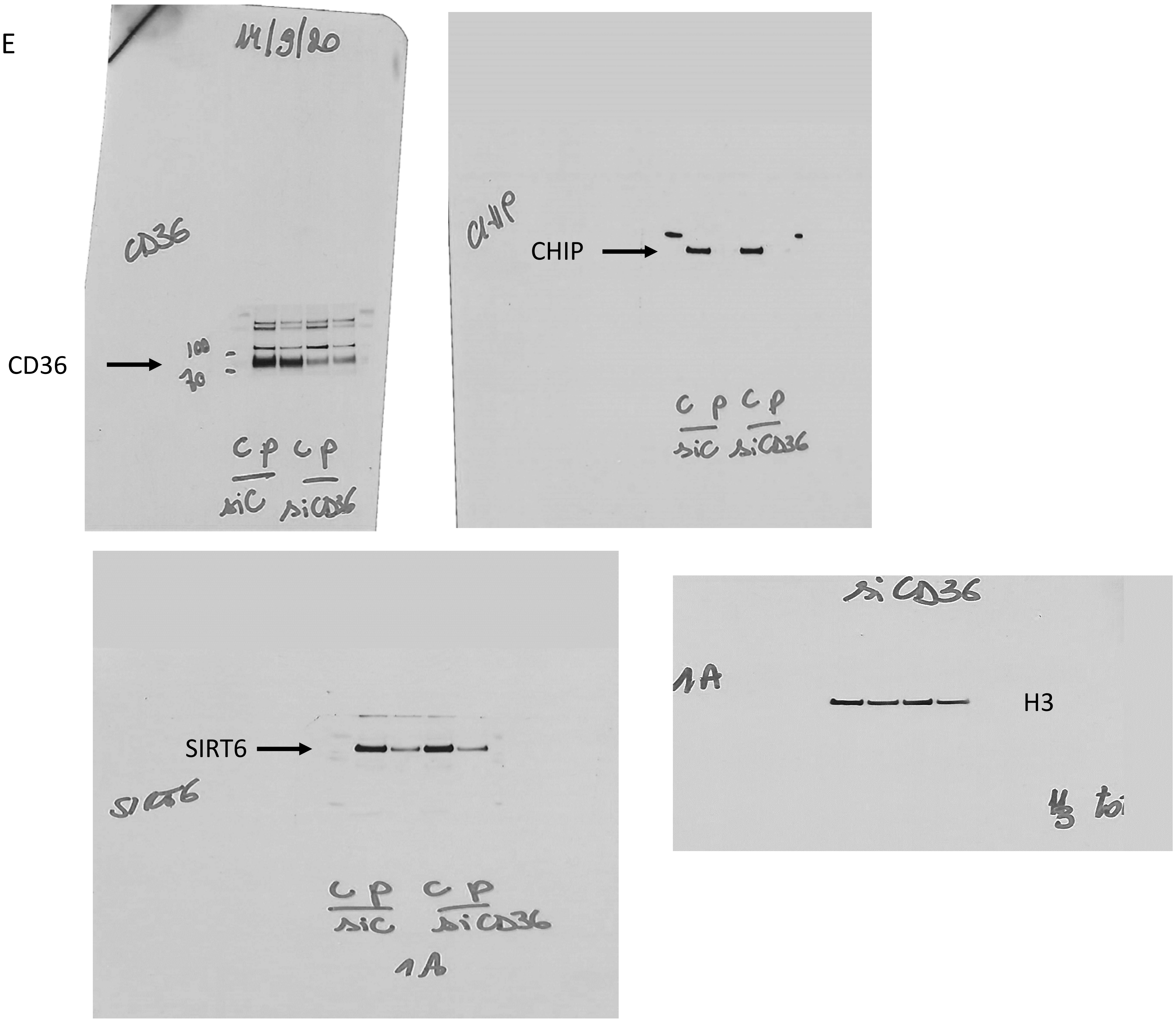

Full unedited gel for Suppl Fig. III B

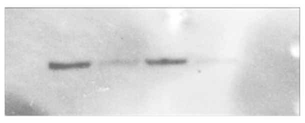

CHIP

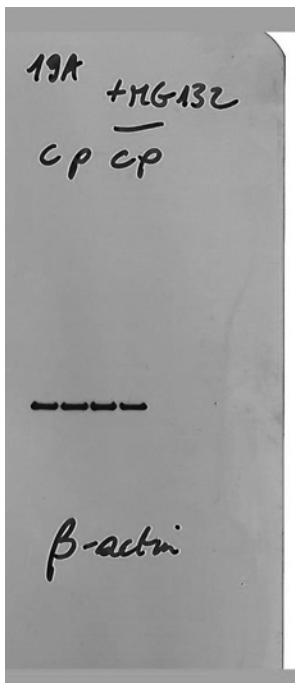

β-actin

Full unedited gel for Suppl Fig. III C

N=2 on one gel

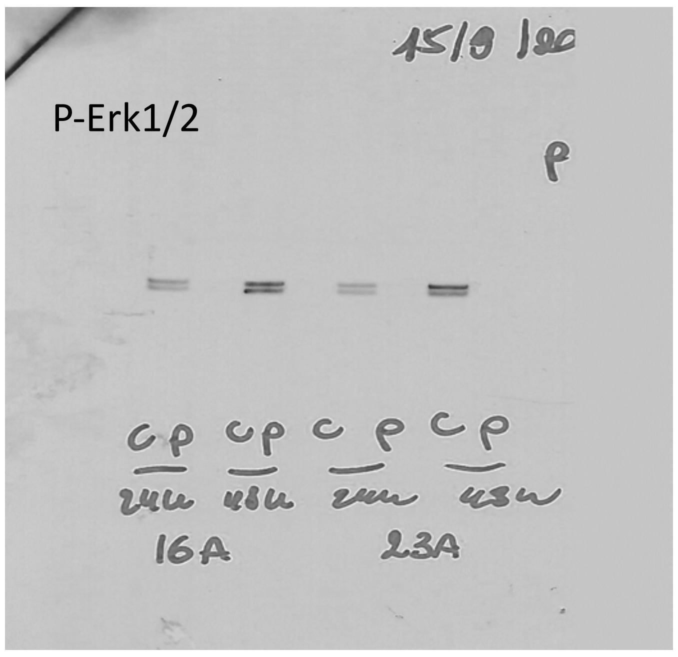

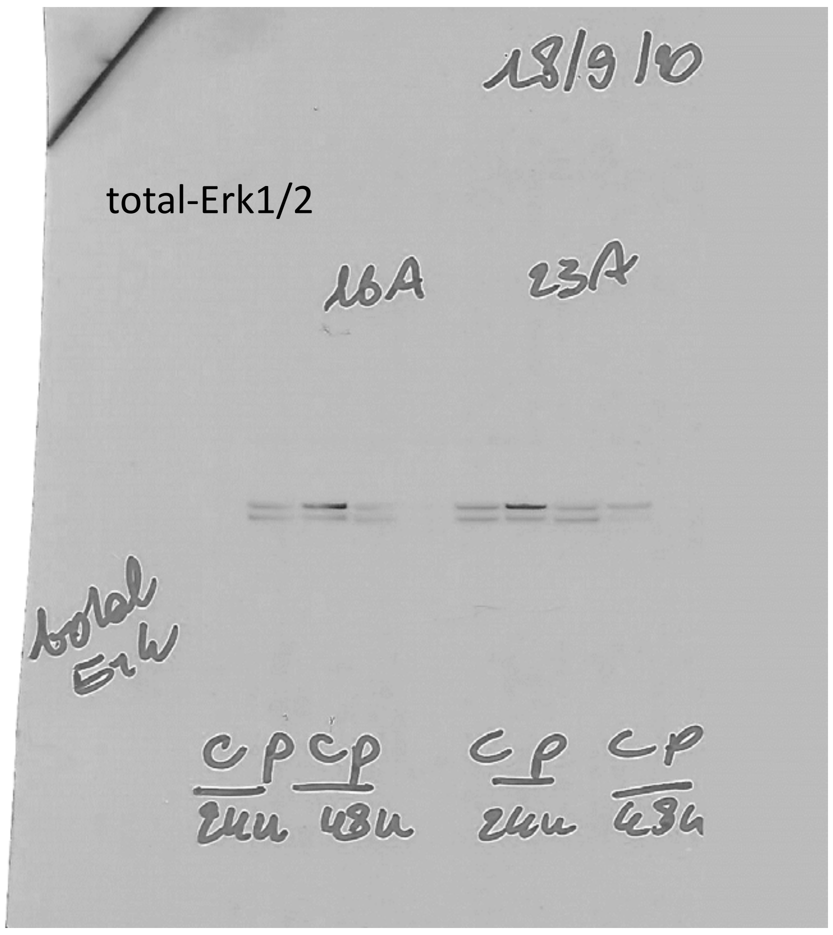

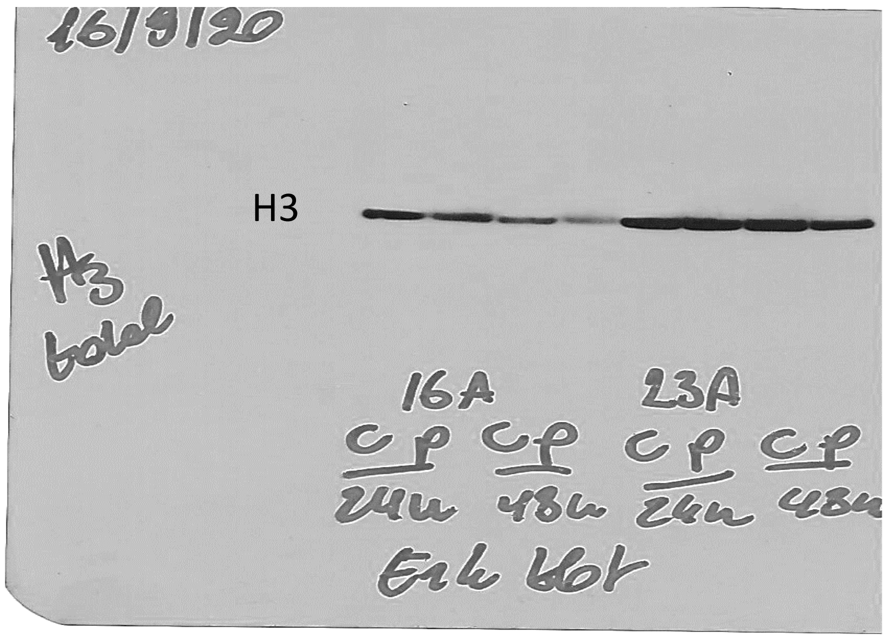

Full unedited gel for Suppl Fig. V B

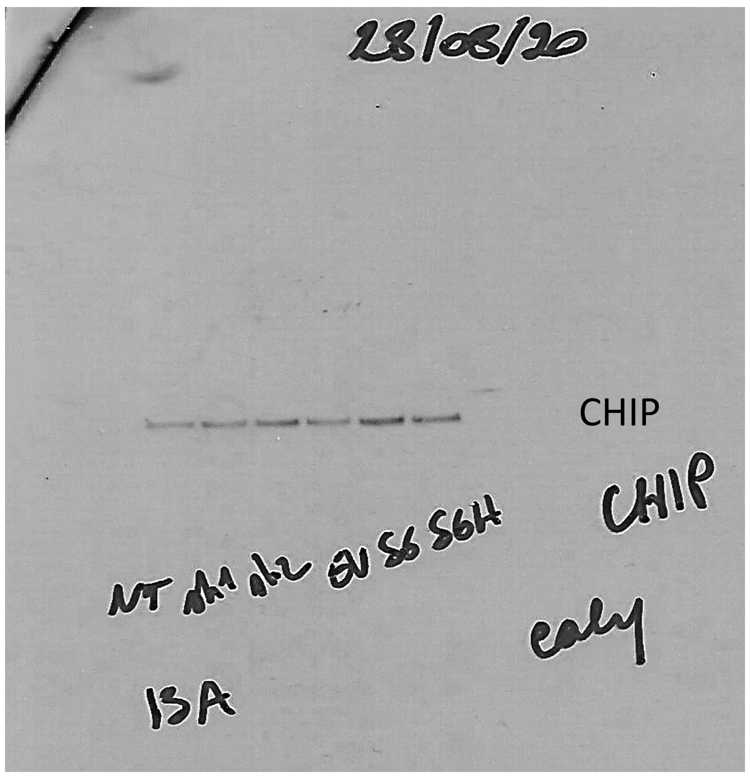

shNT/sh#1/sh#2/EV/S6/S6H

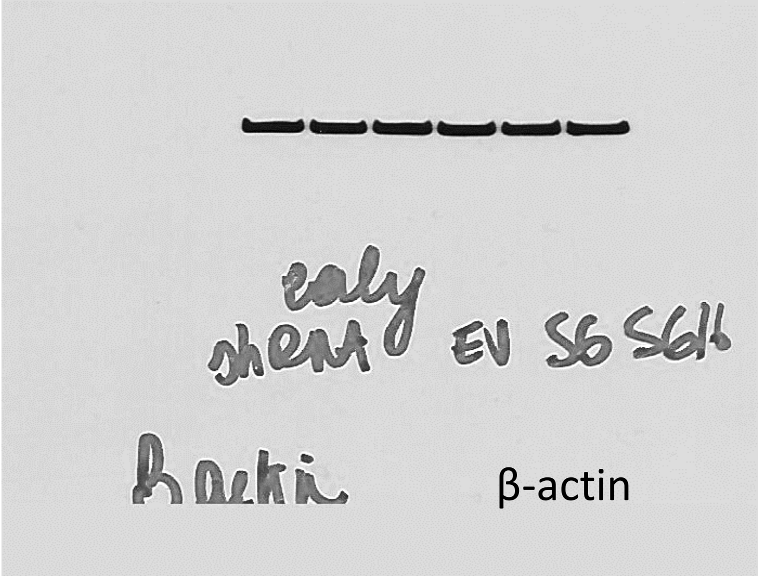

shNT/sh#1/sh#2/EV/S6/S6H

Full unedited gel for Suppl Fig. VI C

2x early | late

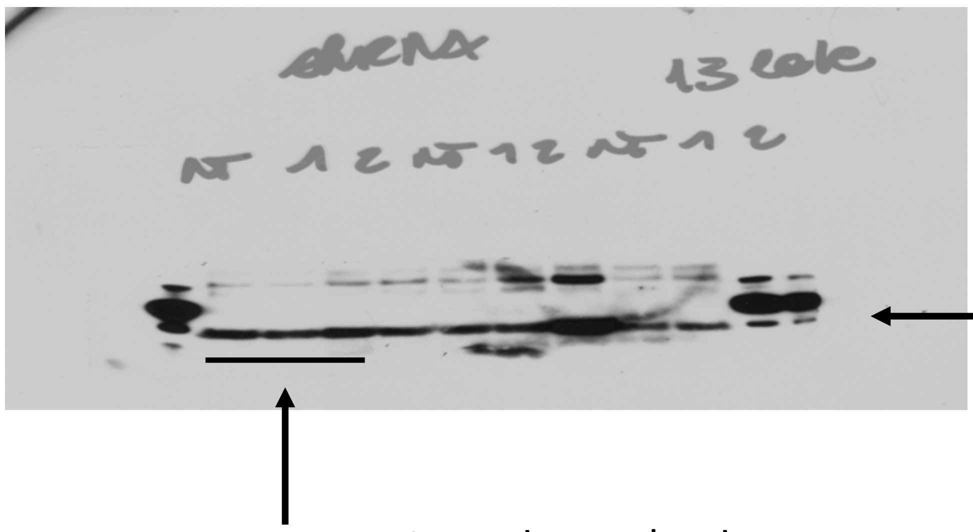

γH2AX

shRNA

N=2 for 'early' and N=1 for 'late' all on one gel

2x early | late

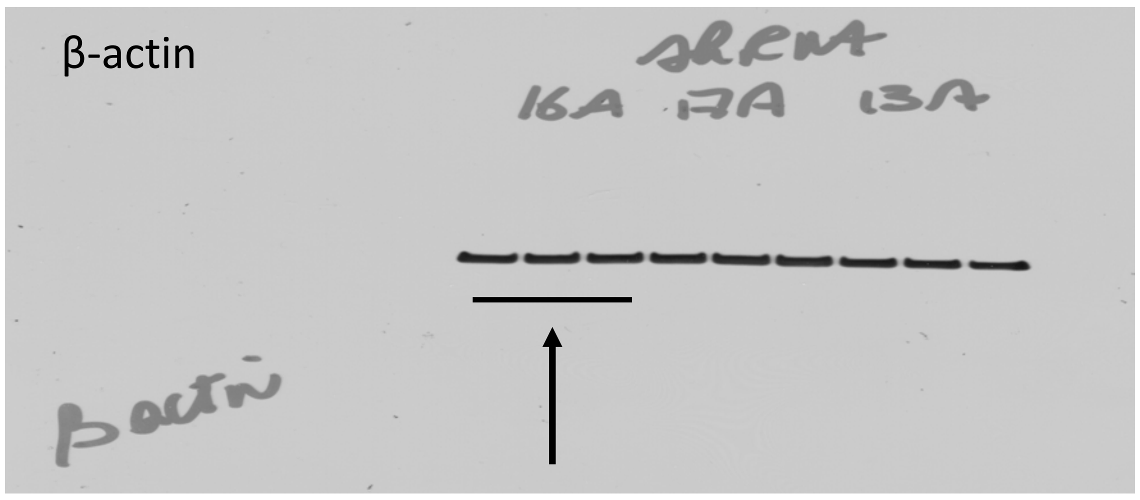

2x early | late

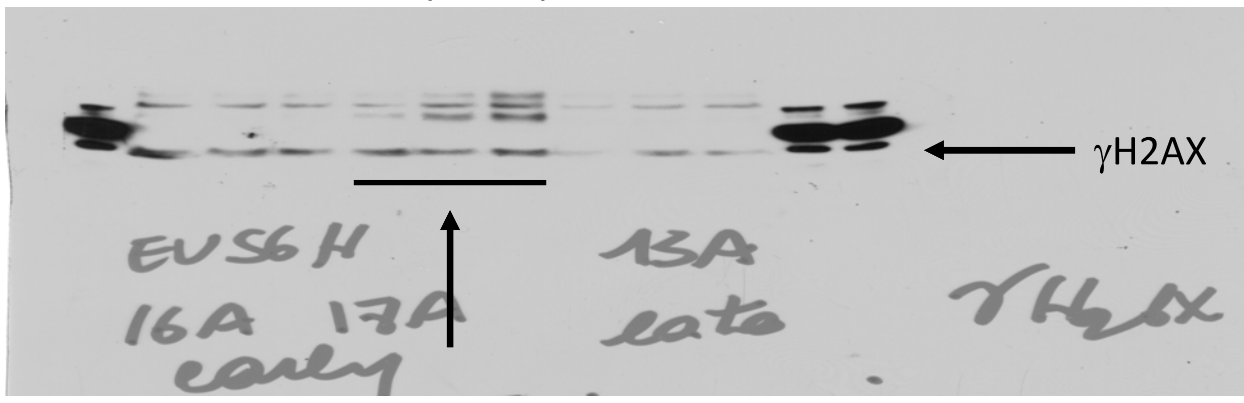

EV/S6/S6H

N=2 for 'early' and N=1 for 'late' all on one gel

2x early | late

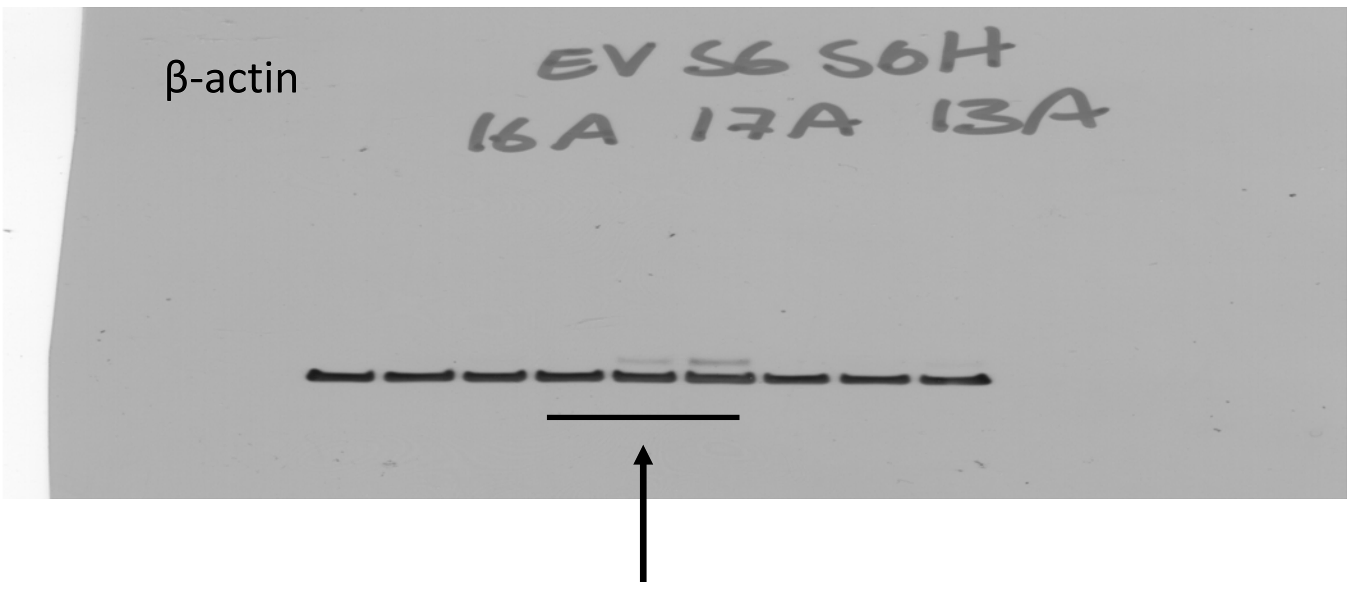

Full unedited gel for Suppl Fig. VI D

N=2 for ‘early’ and N=1 for ‘late’ all on one gel

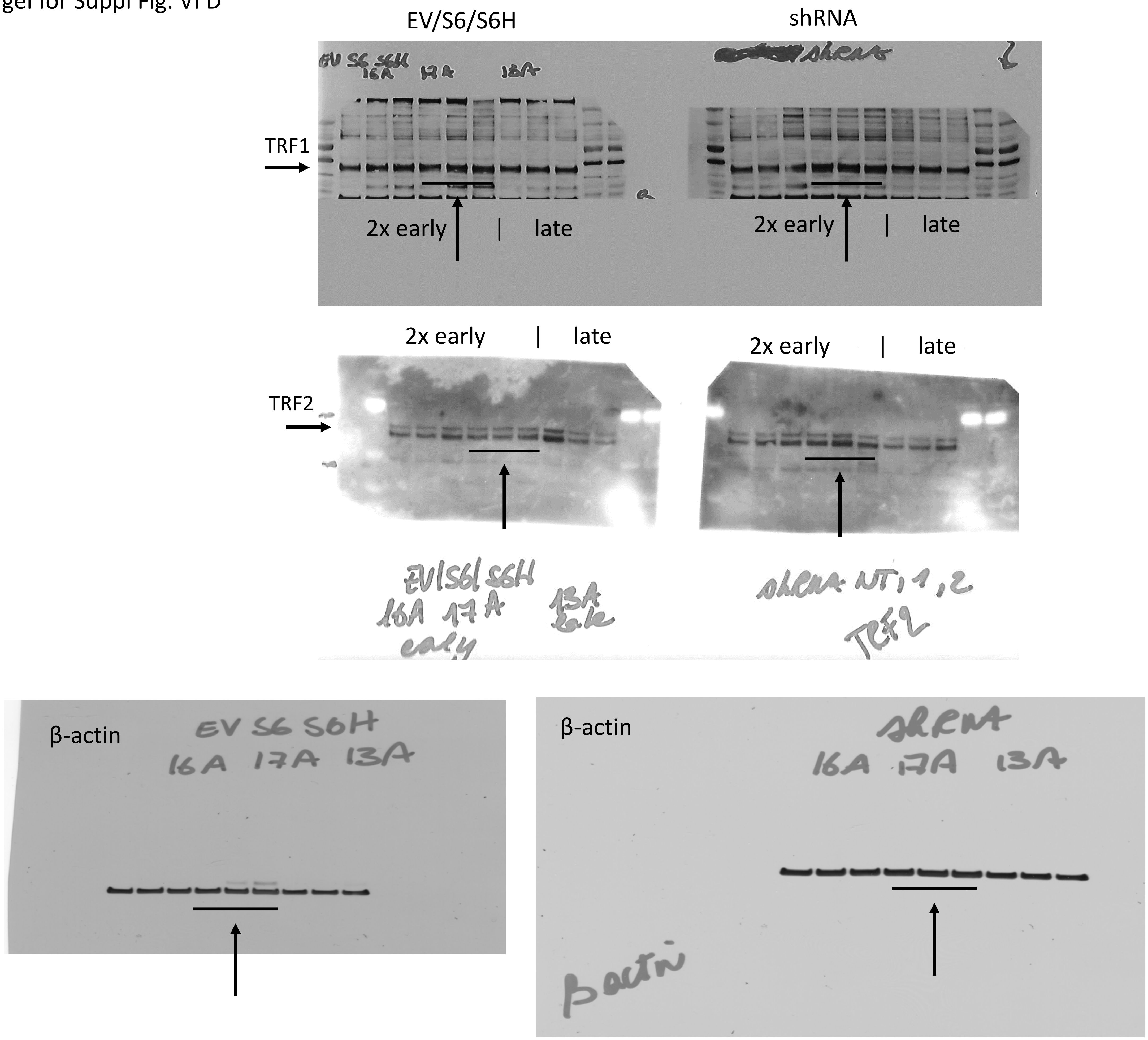

Supplement: Supplementary file 2 [file res-128-474-s002.pdf]
