# Supplementary material for: SIRT6 Protects Smooth Muscle Cells From Senescence and Reduces Atherosclerosis
Source: Circ Res. 2020 Dec 23;128(4):474–91. doi: 10.1161/CIRCRESAHA.120.318353 (PMC7899748; doi:10.1161/CIRCRESAHA.120.318353)
Supplement: Supplementary file 3 [file res-128-474-s003.pdf]

SUPPLEMENTAL MATERIAL

METHODS

Cell culture

Human VSMCs (hVSMCs) were cultured in hVSMC-specific medium (Promocell, SMC-GM2, C22062) supplemented with 100U/ml penicillin, 100μg/ml streptomycin and 5% FCS according to the manufacturer's protocol. Human VSMCs were cultured from aortas from patients undergoing cardiac transplant or aortic valve replacement and plaque VSMCs were isolated from carotid endarterectomies. After removing the endothelial layer and adventitia from the aortic tissue, tissues were cut into 2-3mm² pieces, placed into 6-well plates containing 1ml media and grown for 1-2 weeks to allow cells to emerge. Human VSMCs were studied at passages 2–10 or until senescence was reached. VSMCs derived from individual patients were not pooled.

Mouse aortic VSMCs (mVSMCs) were prepared by enzymatic digestion using Type II collagenase (1mg/ml, Gibco) and elastase (0.74U/ml, Worthington Biochemical). After carefully removing the adventitia, the aorta was further digested for 1h at 37°C. VSMCs were washed and then plated in a 24-well plate in DMEM/F12 medium (11320-074, Gibco) supplemented with 100U/ml penicillin, 100μg/ml streptomycin. After 3 weeks, mVSMCs were used for experiments until they reached passage 8. For each isolation, 2 aortas were pooled.

For some experiments, hVSMCs were treated with MG132 (BML-PI102-0005, Enzo), cycloheximide (01810, Sigma), JNK inhibitor SP600125 (1496, Tocris), p38 inhibitor SB203580 (BML-EI286-0001, Enzo), doxorubicin (hydrochloride) (15007, Cayman Chemicals) and/or palmitate. Palmitate was freshly prepared for each experiment by a conjugation reaction with BSA. Briefly, sodium palmitate (P9767, Sigma) was dissolved in sterile PBS and heated at 70°C for 10min. Next, palmitate was added to fatty acid-free BSA (A8806, Sigma) whilst heating at 50°C for 5min, to obtain a final 30mM palmitate-10% BSA solution. 10% BSA solution in PBS was used as a control vehicle.

Annexin V/PI flow cytometry

hVSMCs were treated with H_2O_2 (100μM) for 2h and analyzed by Annexin V/PI labeling using the Dead Cell Apoptosis kit (V13242, Invitrogen). Briefly, 5×10^4 cells were collected, washed with PBS and resuspended in 200μl Annexin-binding buffer containing 5μl FITC-conjugated Annexin V (10μg/ml) and 1μl PI (1μg/ml). The cell suspension was incubated in the dark for 15min at RT, topped up with 100μl of Annexin-binding buffer and analyzed within 1h using the Accuri C6 flow cytometer (BD Biosciences). A total of 10,000 events were recorded for each sample.

EdU incorporation assay

To asses proliferation, cells were analyzed using the Click-iT EdU kit (C10340, Thermo Fisher Scientific). Briefly, 3×10^4 cells were grown on coverslips and incubated with EdU (10μM) for 24h. Next day, cells were fixed, permeabilized and incubated with the Click-iT reaction cocktail (containing $CuSO_4$, Alexa fluor 647 azide, reaction buffer and additive) for 30min protected from light. Next, cells were washed and stained with DAPI for 10min, and then mounted on glass slides using Prolong Glass Antifade Mountant (P36980, Invitrogen). The following day, cells were imaged using a Leica TCS SP5 confocal laser scanning microscope and the number of EdU-positive cells quantified using ImageJ software.

Senescence-associated beta galactosidase (SAβG) staining

Senescence was determined using the Senescence Cells Histochemical Staining Kit (CS0030, Sigma-Aldrich). Only 3×10^4 cells were plated in a 12-well plate to avoid a high degree of confluence. The following day, cells were fixed with 1x Fixation Buffer for 7min at RT, washed and incubated with staining mixture (containing X-gal) at 37°C for 5h. Images were taken using a Nikon TMS-F microscope equipped with GXCAM LITE live camera. The percentage of SAβG-positive cells was quantified using ImageJ software.

Cloning

A pLX302 lentiviral vector that expresses V5-tagged SIRT6 cDNA (gift from Prof. Jorge Erusalimsky, Cardiff Metropolitan University, UK) was used to stably overexpress hSIRT6 in hVSMCs. The pLX302-SIRT6^{H133Y} mutant was created by site-directed mutagenesis (200523, QuickChange II SDM kit, Agilent Technologies, Ca, USA) using the following primers: 5' AAACTGGCAGAGCTCTACGGGAACATGTTTGTGG-3' (forward) and 5'-CCACAAACATGTTCCCGTAGAGCTCTGCCAGTTT-3' (reverse). To stably overexpress CHIP in hVSMCs, Myc-tagged CHIP cDNA was cloned into pLX302 using BsrG1 (restriction site introduced by PCR cloning) and AgeI restriction enzymes. The pCDNA3.1 CHIP plasmid was a kind gift from Prof. Jonathan C. Schisler (University of North Carolina, USA).

Transfections and lentiviral infections

Lentiviruses were produced to create stable overexpression of wild-type SIRT6 or deacetylase-incompetent SIRT6^{H133Y} in human VSMCs. plx302 vectors encoding V5-tagged SIRT6 or SIRT6^{H133Y} (see cloning section in main manuscript) and third generation lentivirus plasmids (pRRE, pRSV-Rev and pMD2.G) were used to transfect HEK293FT cells using Trans-iT-LT1 transfection reagent (Mirus MIR2300). As a control, plx302 vector encoding V5-luciferase was used, further referred to as 'empty vector' (EV). To create hVSMCs with stable knockdown of SIRT6, GIPZ lentiviral shRNA vectors targeting the 3'UTR region of SIRT6 were used (further referred to as sh#1 and sh#2) (Dharmacon, RHS4531-EG51548). A non-silencing shRNA vector (Dharmacon, RHS4346) was used as control. Lentivirus-containing medium was collected and pooled 24h and 48h after transfection and concentrated using Lenti-X concentrator (631232, Takara). Lentivirus titers were determined by flow cytometry for either V5 or GFP (expressed in shRNA vectors), 48h after transduction. For final experiments, hVSMCs were infected with lentivirus using MOI of 5 in addition of Polybrene (4μg/ml) (H9268, Sigma). 48h after transduction, hVSMCs were selected for puromycin resistance for 2 weeks by gradually reducing the dose of puromycin (P8833, Sigma) from 1mg/ml to 0.1mg/ml. After puro-selection, cells were validated by real time QPCR and Western blot analysis. A similar protocol was used to produce lentivirus to overexpress CHIP (see cloning section). For the production of a lentiviral vector expressing TRF2 mutant TRF2^{T188A}, a pLenti-TRF2^{T188A}-Myc-DDK vector was generated from pLenti-TRF2-Myc-DDK (purchased from OriGene) by site-directed mutagenesis.

To transiently silence CHIP or CD36, hVSMCs were transfected with 100nM human CHIP-specific siRNA (ON-TARGET*plus*® SMART Pool, Dharmacon, L-007201-00-0005) or CD36-specific siRNA (ON-TARGET*plus*® SMART Pool, Dharmacon, L-010206-00-0005) respectively or siRNA control (ON-TARGET*plus*® Control Pool, nontargeting pool, Dharmacon, D-001810-10-05) using Lipofectamine RNAiMAX transfection reagent (13778030, Invitrogen). Silencing efficiency was assessed by real time QPCR and Western blotting.

Real-time quantitative polymerase chain (QPCR) reaction

RNA extraction from human and mouse VSMCs was performed using the NucleoSpin kit (740955.5, Machery-Nagel). For RNA extraction from mouse tissues, a RNeasy Mini kit (74104, Qiagen) and Qiazol extraction method was used. cDNA synthesis was performed using QuantiTect Reverse Transcriptase (205311, Qiagen). Quantitative real-time PCR was performed using Rotor-Gene SYBR Green PCR Kit (20474, Qiagen) on a Rotor-Gene 6000 QPCR thermocycler (Corbett Research). All primer sequences are listed in **Table I**. In some experiments, Taqman gene expression probes were used **(Table I)**.

Western blotting and immunoprecipitation (IP)

Whole cell protein lysates were prepared in fresh RIPA buffer, supplemented with proteinase inhibitors (Millipore), phosphatase inhibitors (Millipore) and deacetylase inhibitor Trichostatin A (500nM) (T8552, Sigma) when applicable. For the analysis of ubiquitination, samples were collected in buffer supplemented with EDTA and N-Ethylmaleimide to prevent de-ubiquitination. For IP experiments, Dynabeads protein G (10004D, Invitrogen) and freshly-made lysis buffer containing 0.5% NP-40, 50mM Tris pH7.5, 150mM NaCl and 0.5mM EDTA were used, supplemented with proteinase inhibitors. Mouse IgG1κ control (ab18443, Abcam) was used as isotype control. Protein concentration

was determined using BCA method (23227, Pierce BCA protein assay kit, Thermo Fisher). Immunoblotting was performed according to standard conditions, using 4-12% polyacrylamide gels, methanol-based wet transfer and chemiluminescence detection (Amersham ECL detection reagent, GE Healthcare). Primary antibodies were detected using HRP-labelled secondary antibodies: goat anti-rabbit (7074S, CST) and horse anti-mouse (7076, CST). β-actin or total histone 3 were used as loading controls. Antibody details are listed in **Table II**.

Telo-ChIP
Chromatin immunoprecipitation (ChIP) was performed on 2×10^6 cells using the ChIP-IT express kit (53008, Active Motif) according to manufacturer's protocol. Cellular proteins and DNA were cross-linked for 10min with formaldehyde (1% final concentration). Cells were then collected and lysed to release the nuclei. Next, chromatin was sheared (2x 5 pulses of 30sec with a 30sec rest interval) using a Bioruptor UCD-200 ultrasound sonicator (Diagenode), resulting in DNA fragments of 200– 1500bp in size. The sheared chromatin was immunoprecipitated with 3μg rabbit anti-SIRT6 (ab62739, Abcam), anti-acetyl-histone 3 (Lys9) (9649, CST), or anti-53BP1 (NB100-304, Novus) antibody, or negative control rabbit IgG (ab37415, Abcam). Protein-G coated magnetic beads were used to capture the antibody-bound protein/DNA complexes. The DNA was eluted, reversed cross-linked, digested with proteinase K and purified using QIAquick PCR purification kit (28104, Qiagen). The purified DNA was then used as a template for quantitative PCR using primers specific for telomere sequences are listed in **Table I**.

Seahorse
Oxygen consumption rate (OCR) and extracellular acidification rate (ECAR) were measured using the Seahorse XF96e Flux Analyzer (Agilent) in basal conditions and upon addition of the following substrates/inhibitors. Freshly made palmitate complexed with 10% BSA and etomoxir (E1905, Sigma) were used to assess fatty acid oxidation. To asses glycolysis, D-(+)-glucose (G8270, Sigma), oligomycin A (75351, Sigma) and 2-deoxyglucose (D6134, Sigma) were used. The glycolysis assay was performed in glucose-free medium. For data analysis, the OCR and ECAR from different independent experiments were normalized to protein and adjusted to baseline (=100%). The maximal respiration and glycolytic capacity were calculated according to the method described by the manufacturer: Maximal respiration = maximal respiration after palmitate - minimal respiration after etomoxir (or non-mitochondrial oxygen consumption). Glycolytic capacity = last measurement before addition of glucose (or non-glycolytic acidification) - maximal measurement after addition of oligomycin.

Generation of transgenic mice
To generate transgenic mice carrying a VSMC-specific overexpression of human SIRT6 or its catalytic inactive mutant (hSIRT6^{H133Y}), V5-tagged hSIRT6 cDNA from plX302-SIRT6 vector was subcloned between the minimal SM22α promoter and polyA sequence in pBluescript II ks+ vector, using BamH1 and EcoR1 restriction sites added by PCR cloning. The hSIRT6^{H133Y} mutant was created by site-directed mutagenesis (200523, QuickChange II SDM kit, Agilent) using the following primers: 5'-AAACTGGCAGAGCTCTACGGGAACATGTTTGTGG-3' (forward) and 5'-CCACAAACATGTTCCCGTAGAGCTCTGCCAGTTT-3' (reverse). The SM22α-hSIRT6 and SM22α-hSIRT6^{H133Y} transgenes were digested with FspI and XhoI, and the transgene-containing fragment purified from an agarose gel using Gel extraction kit (28704, Qiagen), according to the manufacturer's protocol. Transgenic mice were generated by pro-nuclear injection of SM22α-hSIRT6 and SM22α-hSIRT6^{H133Y} fragments directly into C57/BL6J embryos, and positive progeny used to generate 2 founders for each transgenic line. Next, mice were crossed with ApoE$^{-/-}$ mice for atherosclerosis studies. In all experiments, mice heterozygous for hSIRT6 or hSIRT6^{H133Y} were used.

Genotyping
Ear biopsies were incubated with Chelex/Proteinase K for 2h at 56˚C, followed by heat inactivation at 95˚C. DNA samples were prepared in KAPA2G Fast Genotyping Mix (KK5121, Roche). The following PCR genotyping protocol was used for ApoE: 94˚C for 3 min, followed by 32 cycles of 94˚C for 30s, 68˚C for 40s and 72˚C for 1min, with final extension at 72˚C for 5min. hSIRT6 transgene expression

was analyzed using primers that amplify a 550bp region of hSIRT6 fused to the C-terminal V5-tag using the following cycling conditions: 94˚C for 3min, followed by 30 cycles of 94˚C for 30s, 65˚C for 30s and 72˚C for 30sec, with final extension at 72˚C for 3min. PCR products were analyzed on a 2% agarose gel containing GelRed. Primer sequences used for genotyping are listed in **Table I.**

Atherosclerosis studies
For atherosclerosis studies, priori power calculations were made to determine the group sizes using the following equation: $n = 2\,\frac{S^2\,(Z_{1-\alpha/2} + Z_\beta)^2}{\Delta^2}$. Bases on previous experiments we expect atherosclerotic plaques to be approx. 500 000 μm^2 after 16 weeks of HFD, whereby we would need at least a 20% reduction in plaque size (= 100 000μm^2 = Δ) with an standard deviation of 90 000μm^2 (= S) to determine a significant difference between 2 groups. For a 5% significance level ($Z_{1\text{-}a/2}$ = 1.96) and 80% power (Z_b = 0.84), a group size of 13 mice is required. To account for potential loss of mice due to illness or sudden death, group sizes of 14-16 mice were chosen. Animal randomization was achieved by the following measures: 1) littermate controls from both SM22α-hSIRT6 and SM22α-hSIRT6^{H133Y} mouse colonies were used; 2) SM22α-hSIRT6 mice and control littermates were housed in the same cages during colony expansion and atherosclerosis experiment; same for SM22α-hSIRT6^{H133Y} mice and controls. 3) A group of SM22α-hSIRT6 (or SM22α-hSIRT6^{H133Y}) and littermate control mice were always sacrificed on the same day; 4) Both SM22α-hSIRT6 and SM22α-hSIRT6^{H133Y} groups underwent the atherosclerosis experiment within the same time frame. No mice died during the 16 week atherosclerosis study. At the end of the experiment, 4 mice were excluded from the study due to illness or technical reasons: two WT and one SM22α-hSIRT6^{H133Y} mice were excluded due to sickness (splenomegaly (spleen weight ≥ 450mg), heart weight ≥ 250mg, body weight loss) and one WT was excluded due to ambiguous genotype. Outliers, defined as values higher than mean + (STD * 1.5) or lower than mean - (STD * 1.5), were excluded from analysis.

Blood pressure, serum cytokines and lipid analysis
Blood pressure was determined by tail cuff measurements using the BP-2000 Blood Pressure analysis system (Visitech Systems) according to the manufacturer's instructions. Importantly, mice were trained for 2 days prior to the test day and always analyzed at approximately the same time of day (e.g. 7-9am). On the test day, pre-measurements were taken before the actual readings were recorded. 15 measurements for diastolic and systolic blood pressure were recorded for each mouse. In the event of >5 unrecorded readings per mouse, mice were re-assessed. A V-PLEX pro-inflammatory panel 1 mouse kit (K15048D-1) was used to determine cytokine concentrations in mouse serum. Siemens Dimensions EXL and RXL analyzers were used to determine total cholesterol and triglycerides, and HDL, respectively in mouse serum. The c-LDL was calculated using the Friedewald formula. Samples were analyzed according to the manufacturer's protocol.

Oil Red O staining of the thoracic aorta
Oil Red O (0.2 g, Sigma) was dissolved in 100ml of isopropanol. The stock solution was further diluted with distilled water (6:4) and filtered twice through Whatman No. 1 filter paper. Adventitial and adipose tissue were carefully removed from the aorta, the aorta opened along the longitudinal axis, rinsed in distilled water, rinsed in 60% isopropanol for 30sec, stained with Oil Red O for 15min, rinsed again in 60% isopropanol for 30sec and returned to distilled water. The vessel was mounted on a glass slide and imaged *en face* using an Olympus BX51 microscope equipped with an Olympus Lumenera Infinity 3 digital camera and analyzed using ImageJ software. The percentage of Oil Red O-positive plaque area was determined as a measure for plaque burden.

SAβG staining of brachiocephalic artery plaques
Brachiocephalic arteries were harvested, embedded in OCT and stored at -80C. Frozen 12μm sections were cut and stained for SAβG according to the manufacturer's protocol. In brief, sections were fixed for 7min using the fixative provided in the kit, washed 2x with PBS and incubated with the SAβG staining mixture (containing X-gal, provided in the kit) in a humidified chamber overnight at 37°C. The

next day, sections were washed 2x with PBS and mounted with glycerin-gelatin. Images were taken using an Olympus BX51 microscope equipped with an Olympus Lumenera Infinity 3 digital camera.

Histological Analysis

Atherosclerotic tissues were fixed overnight in 4% formalin and embedded in paraffin. 5μm sections were cut, deparaffinated and hydrated through graded methanol solutions to water. Mouse aortic root atherosclerotic plaque extent and composition were measured and analyzed blindly. Aortic root sections were stained with Masson's Trichrome (HT15 kit, Sigma Aldrich) to analyze total plaque, fibrous cap and necrotic core size. Briefly, slides were preheated in Bouin's solution at 56°C for 15min, cooled and washed in water, and then stained in working Weigert's Iron Haematoxylin solution for 5min. Next, slides were washed in water for 5min, rinsed in distilled water and stained in Biebrich Scarlet Acid Fucshin for 5min. After rinsing in distilled water, slides were stained in working Phosphotungstic/Phosphomolybdic Acid solution for 5min. Then, slides were placed in Aniline Blue solution for 5min, followed by 1% Acetic Acid for 1min, rinsed, dehydrated through alcohol, cleared in Histoclear and mounted in Histomount. The nuclei will be stained black, connective tissue blue and muscle fibers red. Fibrous caps were defined as the area rich in VSMCs (red) and connective tissue (blue) while necrotic cores were distinguished by their cholesterol-rich, matrix-poor and acellular content. Collagen content was measured by a Sirius Red staining (ab245887). Briefly, slides were incubated in Phosphomolybdic Acid Solution (0.2%) for 1-5min, and then quickly dipped in distilled water. Next, slides were incubated in Picro-Sirius Red Solution for 60mins and then quickly rinsed in two changes of Acetic Acid Solution (0.5%). Finally, slides were rinsed in absolute alcohol, dehydrated in 2 changes of absolute alcohol, cleared, and mounted in synthetic resin.

For immunohistochemistry, antigen retrieval was achieved by boiling in citrate buffer (pH 6.0) and endogenous peroxidase activity quenched with hydrogen peroxidase. Sections were blocked with Thermo Scientific Superblock 37580. Aortic root sections were immunostained for mouse α-SMC-actin (1:300 dilution) (M0851, Dako) or rat Mac-3 (1:400 dilution) (553322, BD). Mouse IgG (Vector I2000) or Rat IgG (Vector: BA4001) were used as isotype controls. In some cases, aortic root plaques were double-stained for either rabbit SIRT6 (1:50 dilution) (13572-1-AP, Proteintech) or rabbit CHIP (1:8000 dilution) (NBP2-75440, Novus) and mouse α-SMC-actin (1:300 dilution) (M0851, Dako). After incubation with primary antibodies overnight, sections were washed twice in PBS, incubated for 30min in horse anti-mouse biotin-conjugated antibody, washed in PBS and incubated in Avidin-BiotinComplex, for 30min washed in PBS and incubated with diaminobenzidine (DAB Vector SK4105). For visualization of α-SMC-actin stainings however, a Vector Mouse on Mouse Kit (BMK2202, Vector Laboratories) was used. To validate antibody specificity, rabbit isotype (Vector I1000) controls were used. Mouse thymus and mouse lung carcinoma/human colonic carcinoma sections were used as positive controls for SIRT6 and CHIP, respectively. Sections (5μm) of human carotid plaques and healthy aortas were double-stained for either rabbit SIRT6 (1:500 dilution) (13572-1-AP, Proteintech) or rabbit CHIP (1:10000) (NBP2-75440, Novus) and mouse α-SMC-actin (1:400 dilution) (M0851, Dako) and visualized with Mouse- and Rabbit-Specific HRP/DAB (ABC) Detection IHC kit (ab64264, Abcam). Rabbit IgG (Vector I1000) and mouse IgG (Vector I2000) were used as isotype controls, and mouse thymus and mouse lung carcinoma/human colonic carcinoma were used as positive controls. All images were taken using an Olympus BX51 microscope equipped with an Olympus Lumenera Infinity 3 digital camera and analyzed using ImageJ software.

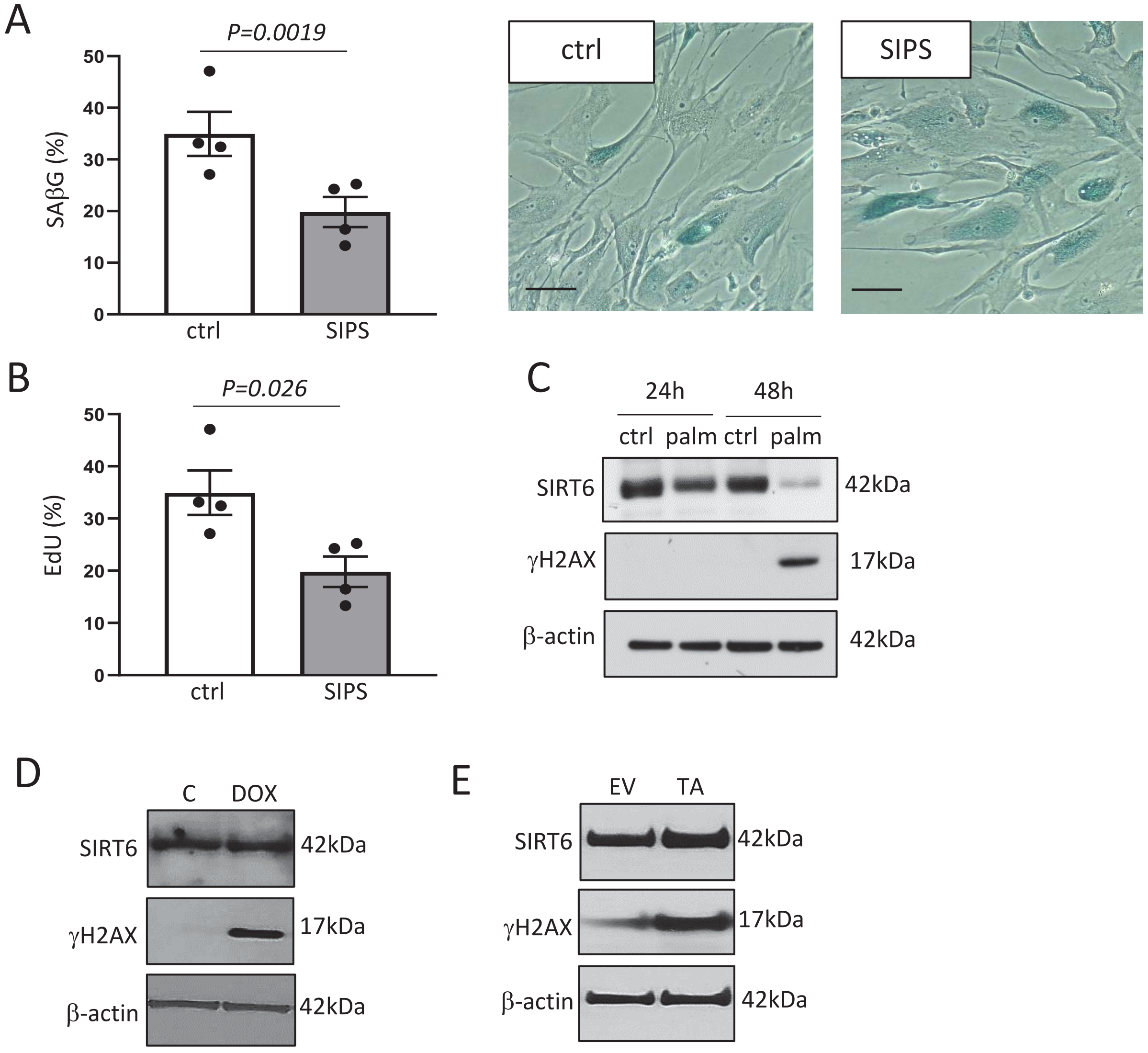

Supplemental Figure I: Palmitate reduces SIRT6, and induces DNA damage and senescence in human VSMCs but DNA damage does not downregulate SIRT6

(A,B) hVSMCs were treated with palmitate (200μM) or 10% BSA control (ctrl) for 24h followed by 3 weeks recovery to induce stress-induced premature senescence (SIPS), determined by **(A)** expression of the senescence marker SAβG (Unpaired t-test, n=4) with representative images of SAβG-positive hVSMCs (scale bar=50μm) and **(B)** proliferation by EdU incorporation assay (Unpaired t-test, n=4). **(C)** hVSMCs were treated with palmitate (palm) or 10% BSA (ctrl) for 24h and 48h and analyzed for SIRT6 and γH2AX expression by western blotting (n=3). **(D)** hVSMCs were treated with doxorubicin (DOX) (500nM) or DMSO as control (C) for 24h and analyzed for SIRT6 and γH2AX expression by western blotting. **(E)** VSMCs infected with an empty lentivirus vector (EV) or lentivirus expressing TRF2^{T188A} (TA), a point mutant of the TRF2 protein leading to telomere dysfunction, were analyzed for SIRT6 and γH2AX expression by western blotting. β-actin was used as loading control. Data are shown as mean±SEM with P-value <0.05.

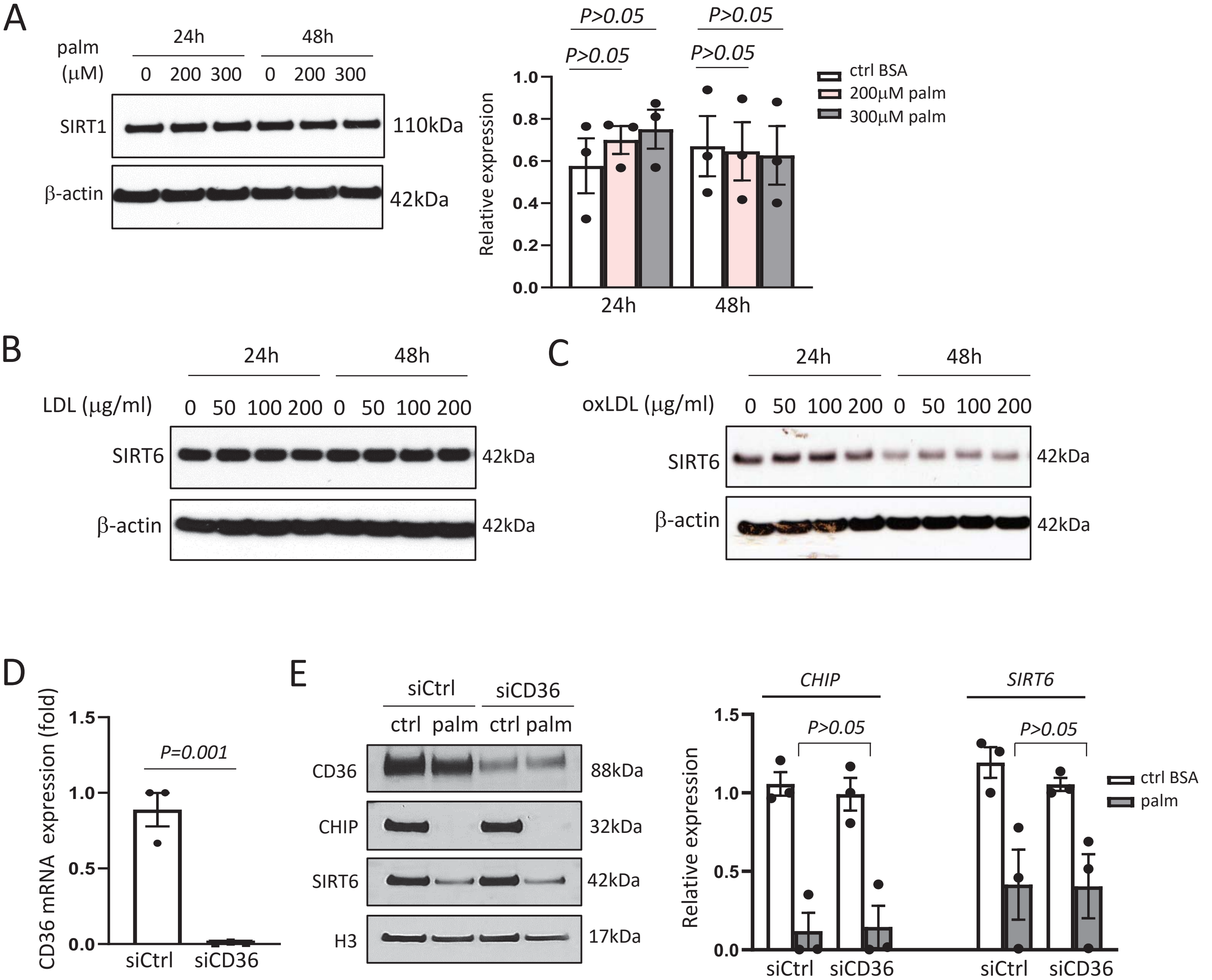

Supplemental Figure II: Palmitate, but not (ox)LDL, reduces SIRT6 protein expression in hVSMCs, independent of CD36

(A) Western blot and quantification for SIRT1 of hVSMCs treated with palmitate (palm, 200μM or 300μM) or 10% BSA as control, for 24h or 48h (2-Way ANOVA, Tukey's post hoc, n=3). **(B,C)** Western blot for SIRT6 of hVSMCs treated with native LDL **(B)** or oxidized LDL (oxLDL) **(C)** at different dosages (50, 100 or 200 μg/ml) for 24h or 48h. β-actin was used as loading control. **(D)** QPCR mRNA analysis for CD36 72h after transient silencing of CD36 (siCD36) vs. control siRNA (siCtrl) (Unpaired t-test, n=3) **(E)** Western blot analysis for CD36, CHIP and SIRT6, and quantification in siCD36 vs. siCtrl hVSMCs after palmitate treatment (300μM) or 10% BSA as control (ctrl) for 48h (2-Way ANOVA, Tukey's post hoc, n=3) . Histone 3 was used as loading control. Data are shown as mean±SEM with nominal P-value <0.05 or multiplicity adjusted P-value <0.05.

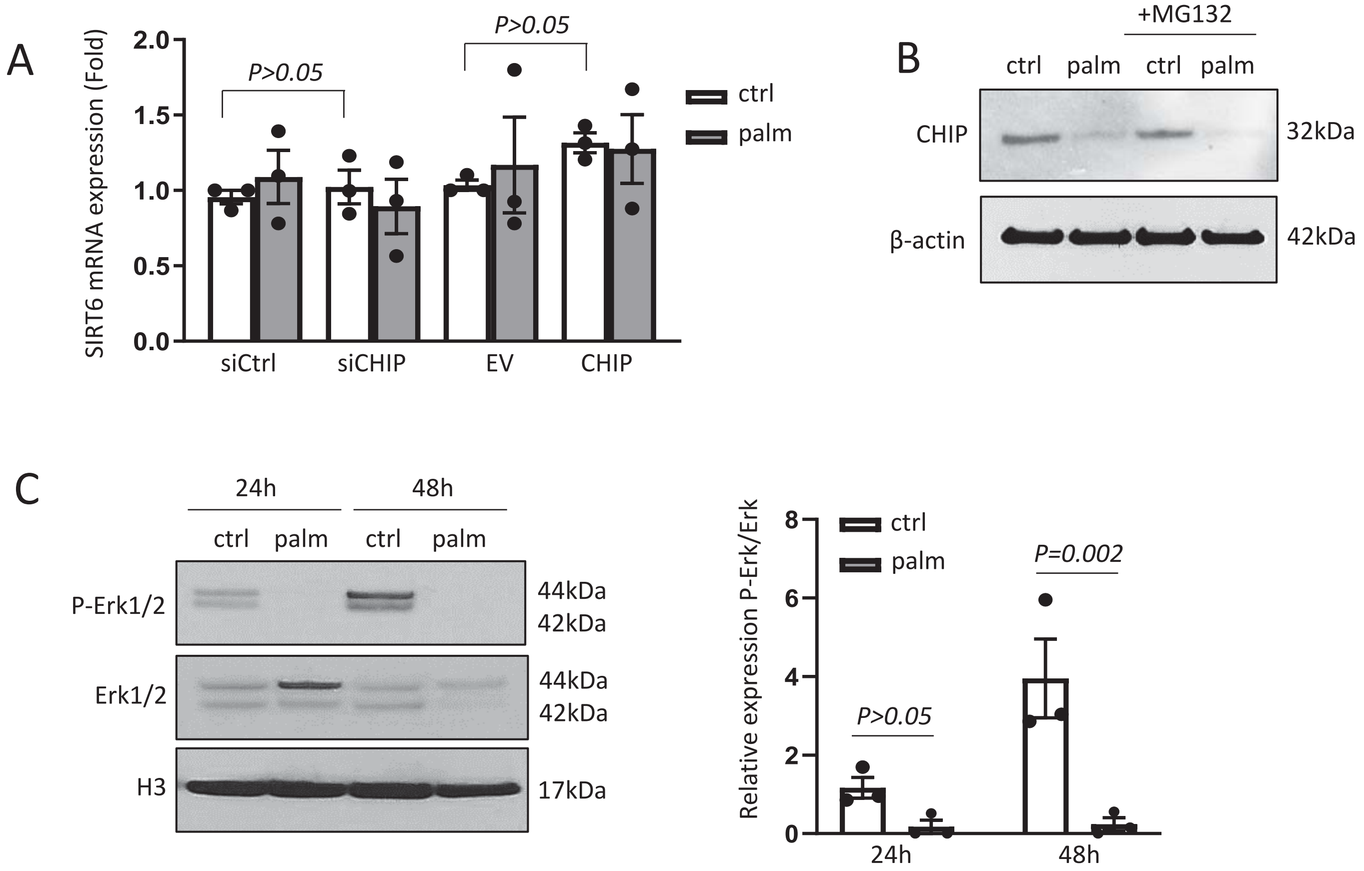

Supplemental Figure III: The ubiquitin ligase CHIP promotes SIRT6 protein stability

(A) QPCR for SIRT6 mRNA expression in hVSMCs transfected with control siRNA (siCtrl) or siRNA to CHIP (siCHIP), or VSMCs infected with an empty lentivirus vector (EV) or lentivirus expressing CHIP, treated with 300μM palmitate or control BSA for 48h (2-way ANOVA, Tukey's post hoc, n=3). **(B)** Western blot for CHIP in hVSMCs treated with 300μM palmitate or control BSA for 48h, with or without the proteasome inhibitor MG132 (1μM). **(C)** Western blot for phospho Erk1/2 and total Erk1/2 in hVSMCs treated with 300μM palmitate (palm) or control BSA (ctrl) for 24h and 48h, with quantification (2-Way ANOVA, Bonferroni post hoc, n=3). Data are shown as mean±SEM with multiplicity adjusted P-value <0.05.

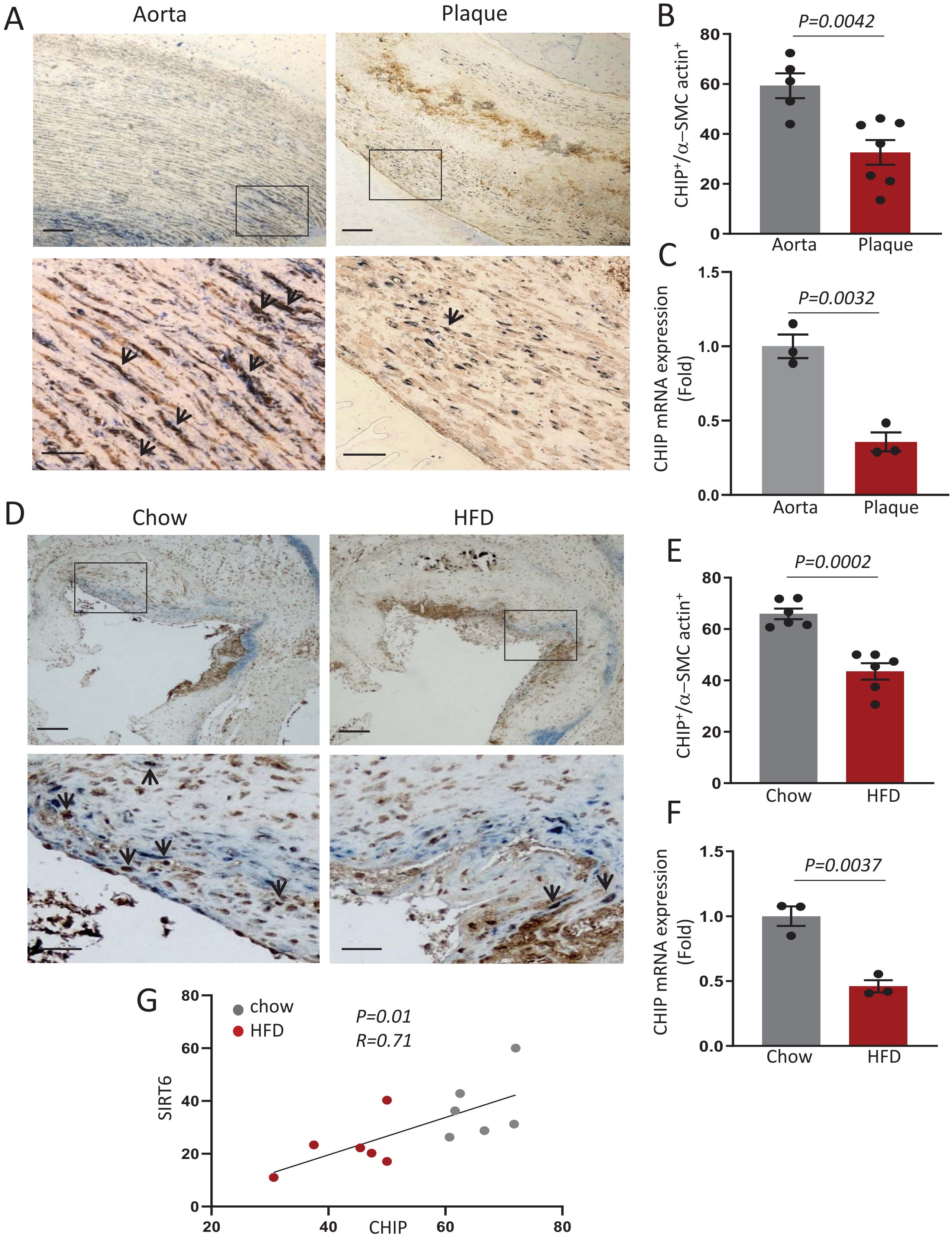

Supplemental figure IV: CHIP expression is reduced in VSMCs in human and mouse atherosclerosis

(A) Immunohistochemistry and **(B)** quantification for α-SMC-actin (blue) and CHIP (brown) of VSMCs from human histologically normal aorta (n=5) and human carotid artery plaques (n=7) (Unpaired t-test). High power images of outlined areas indicate CHIP/α-SMC-actin-positive cells (arrows); scale bar=150μm in high-power views and 75μm in low-power views. **(C)** QPCR for CHIP mRNA of human healthy aortic VSMCs and plaque VSMCs (Unpaired t-test, n=3). **(D)** Immunohistochemistry and **(E)** quantification of cells double positive for α-SMC-actin (blue) and CHIP (brown) in plaques of ApoE$^{-/-}$ mice fed a chow or high-fat diet for 16 weeks (Unpaired t-test, n=6). High-power images of outlined areas indicate CHIP/α-SMC-actin-positive cells (arrows); scale bar=150μm in high-power views and 10μm in low-power views. **(F)** QPCR for CHIP mRNA of mouse aortas of chow or HFD-fed ApoE$^{-/-}$ mice (Unpaired t-test, n=3). **(G)** Correlation between SIRT6/α-SMC-actin and CHIP/α-SMC-actin-positive cells in plaques of ApoE$^{-/-}$ mice on chow (grey dots) or HFD (red dots) (n=6, Pearson's correlation). Data are shown as mean±SEM with P-value <0.05.

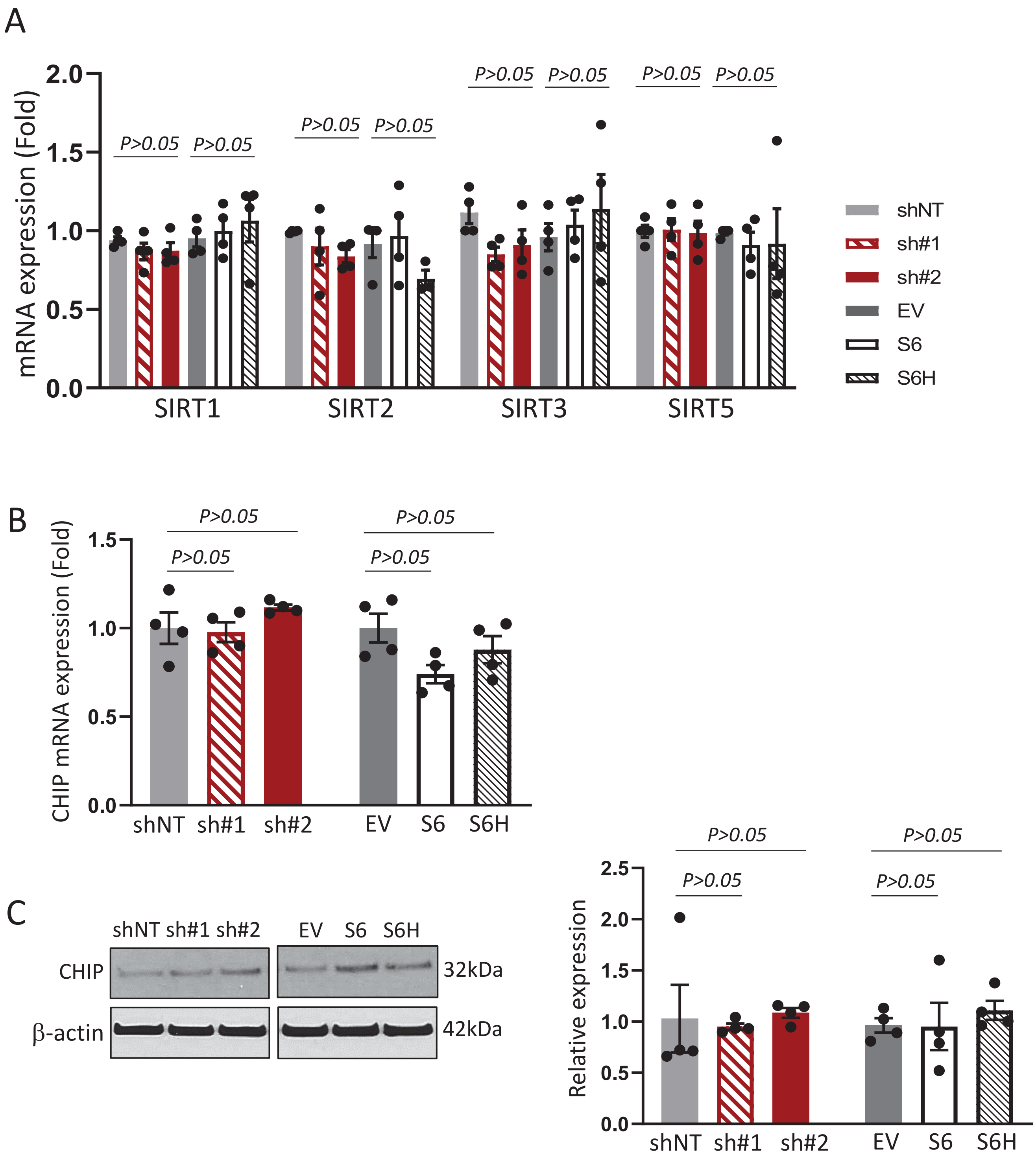

Supplemental Figure V: Chronic silencing or overexpression of human SIRT6 (or SIRT6^{H1333Y}) does not affect expression of other sirtuins or CHIP ligase

(A) QPCR for mRNA expression of Sirtuin 1, Sirtuin 2, Sirtuin 3 and Sirtuin 5 in hVSMCs transfected with control shRNA (shNT) or to SIRT6 (sh#1, sh#2), or VSMCs infected with an empty lentivirus vector (EV) or lentivirus expressing SIRT6 (S6) or SIRT6^{H133Y} (S6H) (1-way ANOVA, nominal P<0.05 values shown, n=4). **(B)** Analysis of CHIP mRNA expression in hVSMCs transfected with control shRNA (shNT) or to SIRT6 (sh#1, sh#2) or VSMCs infected with an empty lentivirus vector (EV) or lentivirus expressing SIRT6 (S6) or SIRT6^{H133Y} (S6H) (1-way ANOVA, Dunnett post hoc, n=4). **(C)** Western blot analysis with quantification for CHIP in hVSMCs transfected with control shRNA (shNT) or to SIRT6 (sh#1, sh#2) (Kruskal-Wallis, Dunn's post hoc, n=4) or VSMCs infected with an empty lentivirus vector (EV) or lentivirus expressing SIRT6 (S6) or SIRT6^{H133Y} (S6H) (1-way ANOVA, Dunnett post hoc, n=4). Data are shown as mean±SEM with nominal P-value <0.05 or multiplicity adjusted P-value <0.05.

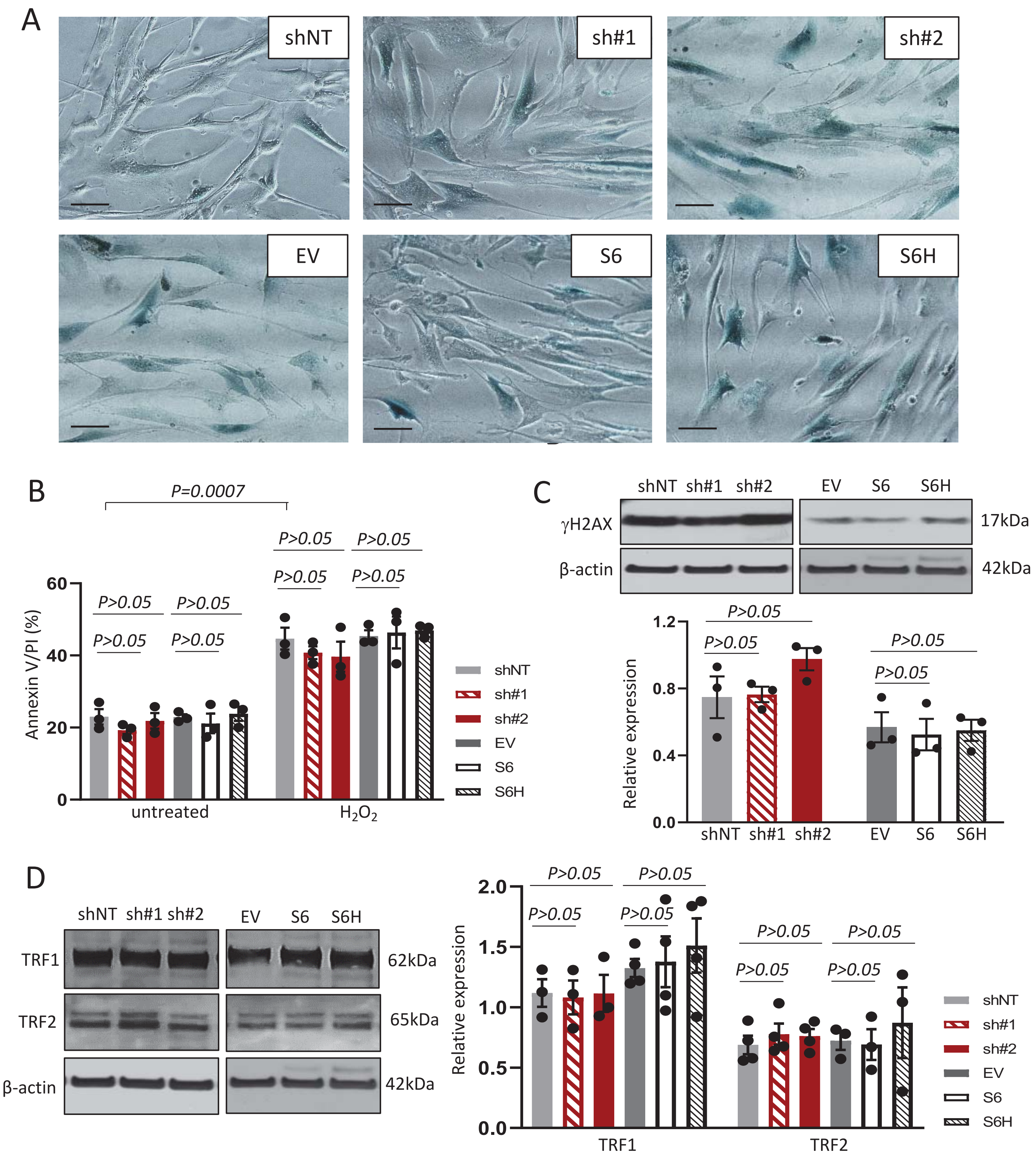

Supplemental Figure VI: SIRT6 attenuates hVSMC senescence without affecting apoptosis, basal global DNA damage and telomere shelterin complex protein expression

(A) Representative images of SAβG-positive hVSMCs infected with control shRNA (shNT) or to SIRT6 (sh#1, sh#2), or VSMCs infected with an empty lentivirus vector (EV) or lentivirus expressing SIRT6 (S6) or SIRT6^{H133Y} (S6H) at late passage (p10-p12) (scale bar=75μm). **(B)** Annexin/PI labeling and flow cytometric analysis for apoptosis in early passage (p4-p6) hVSMCs transfected with control shRNA (shNT) or to SIRT6 (sh#1, sh#2), or VSMCs infected with an empty lentivirus vector (EV) or lentivirus expressing SIRT6 (S6) or SIRT6^{H133Y} (S6H), after exposure to H_2O_2 (100μM, 2h) or in untreated conditions (2-way ANOVA, Tukey's post hoc, n=4) **(C)** Western blot for γH2AX to assess global DNA damage in untreated early passage (p4-p6) experimental hVSMC lines and quantification (1-way ANOVA, Dunnett post hoc, n=3). **(D)** Western blot for TRF1 and TRF2 in experimental hVSMC lines at early passage (p4-p6) and quantification (1-way ANOVA, Dunnett post hoc, n=3). β-actin was used as loading control. Data are shown as mean±SEM with multiplicity adjusted P-value <0.05. SAβG=Senescence-associated beta galactosidase.

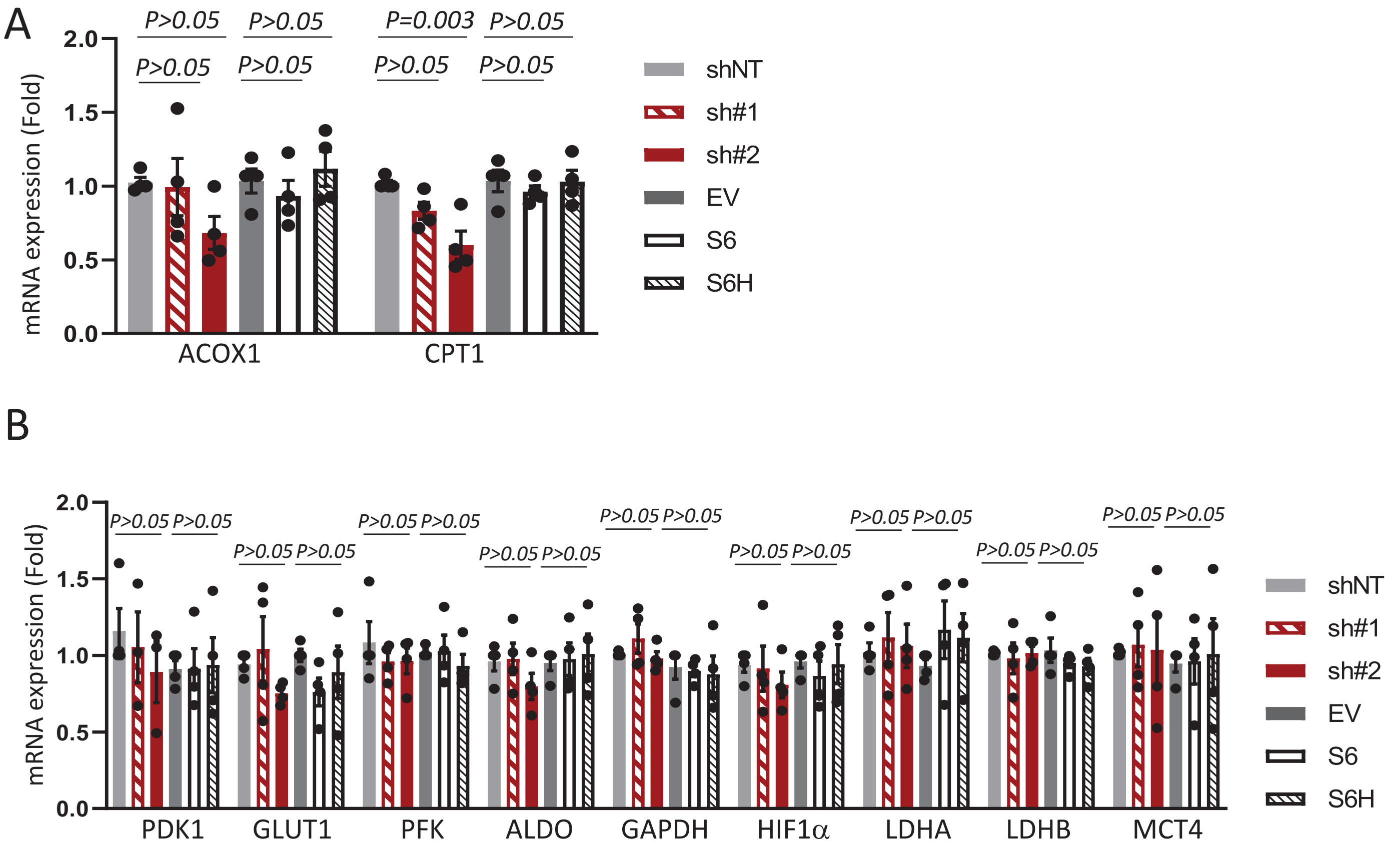

Supplemental Figure VII: SIRT6 does not transcriptionally regulate fatty acid oxidation and glycolysis genes in hVSMCs

(A,B) QPCR for expression of a range of genes involved in **(A)** fatty acid oxidation (1-way ANOVA, Dunnett post hoc, adjusted P<0.05 values shown, n=4) or **(B)** glycolysis (1-way ANOVA, nominal P<0.05 values shown, n=4) in early passage (p4-p6) hVSMCs transfected with control shRNA (shNT) or to SIRT6 (sh#1, sh#2), or VSMCs infected with an empty lentivirus vector (EV) or lentivirus expressing SIRT6 (S6) or SIRT6^{H133Y} (S6H). Data are shown as mean±SEM with nominal P-value <0.05 or multiplicity adjusted P-value <0.05. ACOX1=acyl-CoA oxidase 1. CPT1=carnitine palmitoyltransferase 1; PDK1=pyruvate dehydrogenase kinase 1; GLUT1=glucose transporter 1; PFK=pyruvate fructokinase; ALDO=aldolase; GAPDH=glyceraldehyde 3-phosphate dehydrogenase; HIF1α=hypoxia inducible factor 1 alpha; LDH=lactate dehydrogenase; MCT4=monocarboxylate transporter 4

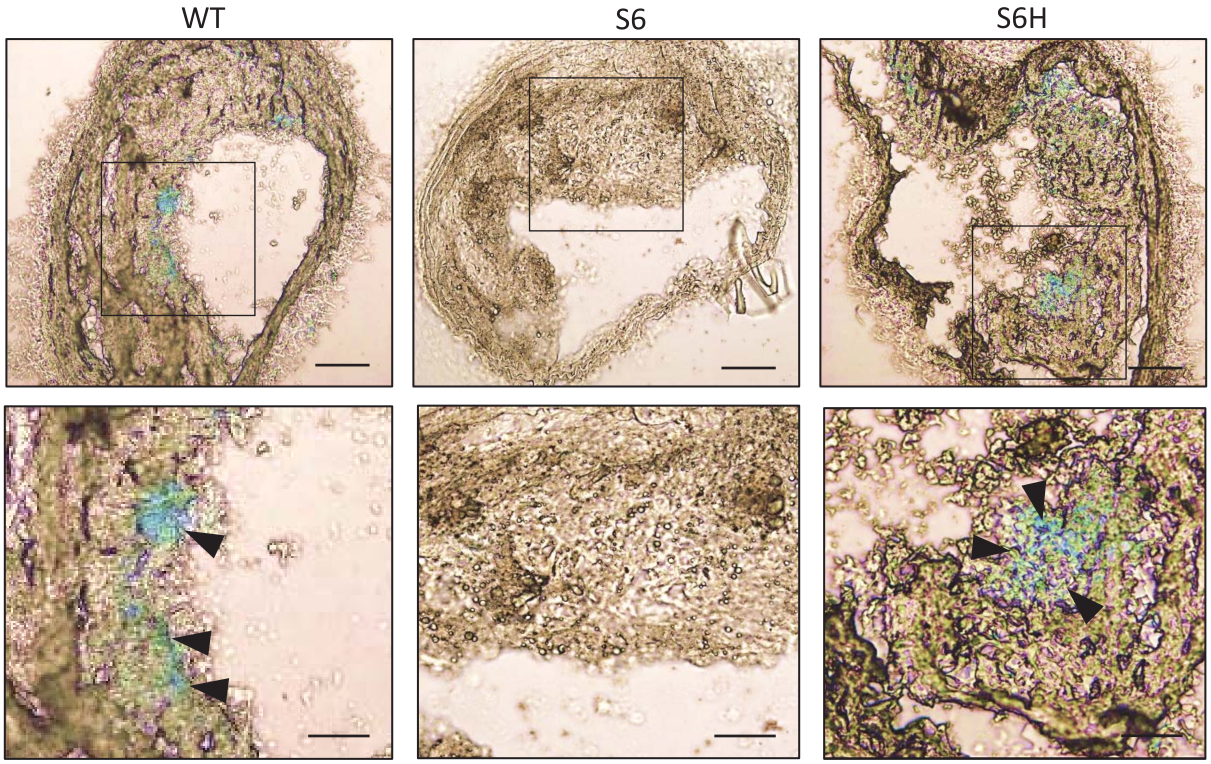

Supplemental Figure VIII: VSMC-specific overexpression of SIRT6 reduces senescence-associated beta galactosidase activity in atherosclerotic plaques

Senescence-associated beta galactosidase (SAβG) activity staining in brachiocephalic artery plaques of wild-type littermate $ApoE^{-/-}$ (WT, n=6), SM22α-SIRT6/$ApoE^{-/-}$ (S6, n=6), and SM22α-hSIRT6^{H133Y}/$ApoE^{-/-}$ (S6H, n=9) mice fed a high fat diet for 16 weeks (scale bar=200μm). Senescent cells are indicated by arrowheads in high power images (scale bar=50μm).

TABLES

Table I. Oligonucleotide sequences and Taqman probes used for (Q)PCR.

**Human DNA primers**	
SIRT6 FW	5'-GCCTGGTCATCGTCAACCTG-3'
SIRT6 REV	5'-TCATGACCTCGTCAACGTAGC-3'
SIRT1 FW	5'-TGACCTCCTCATTGTTATTGGGT-3'
SIRT1 REV	5'-ATCTGAGGCACTTCATGGGG-3'
SIRT2 FW	5'-TCCTGCGGAACTTATTCTCCC-3'
SIRT2 REV	5'-AGCTCCCACCAAACAGATGA-3'
SIRT3 FW	5'-TCACTACTTTCTCCGGCTGC-3'
SIRT3 REV	5'-GGAAGGGTCTTTGGCAGACT-3'
SIRT5 FW	5'-GTCTAGTGGTGGGCACTTCC-3'
SIRT5 REV	5'-GGGGTGGTCTCCGTGTTAAA-3'
CHIP FW	5'-GCTGGAGATGGAGAGCTATGATG-3'
CHIP REV	5'-ATGTCGTCCCCGAAGTTCAGC-3'
PDK1 FW	5'-TCCTGGACTTCGGATCAGTG-3'
PDK1 REV	5'-CCAGTCTGACAGGCAACTCTT-3'
GLUT1 FW	5'-AGCAACTGTGTGGTCCCTAC-3'
GLUT1 REV	5'-CGAAGGTCCGGCCTTTAGTC-3'
PFK FW	5'-CACCATCAGCAACAACGTCC-3'
PFK REV	5'-GCAGACTGTTTGATGCGGTC-3'
ALDO FW	5'-GAGCTGTCTGACATCGCTCA-3'
ALDO REV	5'-CGGTTCTCCTCGGTGTTCTC-3'
GAPDH FW	5'-TGAGAACGGGAAGCTTGTCA-3'
GAPDH REV	5'-GCATCGCCCCACTTGATTTT-3'
HIF1α FW	5'-TTTCCTCAGTCGACACAGCC-3'
HIF1α REV	5'-GAGCGGCCTAAAAGTTCTTCTG-3'
LDH A FW	5'-ACTGCAAACTCCAAGCTGGT-3'
LDH A REV	5'-ACCGCTTCCAATAACACGGT-3'
LDH B FW	5'-TCTGCAGCATGGGAGCTTATT-3'
LDH B REV	5'-CGGACTCCTGCAGTTACCAC-3'
MCT4 FW	5'-GGAGTTTGGGATCGGCTACA-3'
MCT4 REV	5'-GACCCCAGTGGTGAGGTAGA-3'
ACOX1 FW	5'-CCGCCGAGAGATCGAGAAC-3'
ACOX1 REV	5'-GCGAGTGAGGAAGTTCAAGTC-3'
CPT1 FW	5'-AGCTGTGGCCTTTCAGTTCA-3'
CPT1 REV	5'-GGGTACACGCCAGTGATGAT-3'
CD36 FW	5'- TCTAATGCCAGTTGGAGACCTG-3'
CD36 REV	5'- TGTACCTTCTTCGAGGACAACT-3'
IL1α FW	5'- ACTGCCCAAGATGAAGACCA-3'
IL1α REV	5'-TGGTCTCACTACCTGTGATGG-3'
IL6 FW	5'-TAGTGAGGAACAAGCCAGAGC-3'
IL6 REV	5'-CAATAACCACCCCTGACCCA-3'
IL8 FW	5'- CACACTGCGCCAACACAGAAA-3'
IL8 REV	5'- TTTCCTTGGGGTCCAGACAGA-3'
MCP1 FW	5'- AGAAGAATCACCAGCAGCAAGT-3'
MCP1 REV	5'- AGATCTCCTTGGCCACAATGG-3'
RPL13A FW	5-'CGAGGTTGGCTGGAAGTACC-3'
RPL13A REV	5'-CCGTAGCCTCATGAGCTGTT-3'

TELO FW	5'-CGGTTTGTTTGGGTTTGGGTTTGCCTTTGCCTTTGGGTT-3'
TELO REV	5'-GGCTTGCCTTACCCTTACCCTTACCCTTACCCTTACCCT-3'
**Mouse DNA primers**	
SIRT6 FW	5'-TTGTCAACCTGCAACCCACA-3'
SIRT6 REV	5'-ATCACCTCATCCACGTAGCC-3'
SIRT1 FW	5'-AGTAAGCGGCTTGAGGGTAA-3'
SIRT1 REV	5'-CCTCAGCACCGTGGAATATGT-3'
SIRT2 FW	5'-TGACCTCCTCGGATGGAAGAA-3'
SIRT2 REV	5'-CCCTGACTGGGCATCTATGT-3'
SIRT3 FW	5'-GGCATCCCGGACTTCAGAT-3'
SIRT3 REV	5'-GGGTACGGGATGTCATACTGC-3'
SIRT5 FW	5'-ACCATGGCTCGTCCAAGTTC-3'
SIRT5 REV	5'-ATGGCTATGTGCTTGGCGTT-3'
CHIP FW	5'- TGGAGAGTTATGATGAGGCCAT-3'
CHIP REV	5'- AGTTGAGTCGCTGCTCCTTG-3'
ACOX1 FW	5'-TGGTAGTCCGGAGAACACCC-3'
ACOX1 REV	5'-GTCCTCATGTTGGAAGTCTGGA-3'
CPT1 FW	5'-CTTCCAAGTTCTCTAGCCCTGA-3'
CPT1 REV	5'-TCCATCATGGCTTGTCTCAAGT-3'
PDK1 FW	5'-GTTCCTGGACTTCGGGTCAG-3'
PDK1 REV	5'-AACAGGCAACTCTTGTCGCA-3'
GLUT1 FW	5'-AACACTGGTGTCATCAACGCC-3'
GLUT1 REV	5'-TAGCGGTGGTTCCATGTTTGA-3'
p16 FW	5'-TACCCCGATTCAGGTGATGATG-3'
p16 REV	5'-TAGCTCTGCTCTTGGGATTGG-3'
IL1α FW	5'-TTGGTTAAATGACCTGCAACA-3'
IL1α REV	5'-GAGCGCTCACGAACAGTTG-3'
IL1β FW	5'-GCCACCTTTTGACAGTGATGAG-3'
IL1β REV	5'-GACAGCCCAGGTCAAAGGTT-3'
IL6 FW	5'-TCAATTCCAGAAACCGCTATG-3'
IL6 REV	5'-GTCTCCTCTCCGGACTTGTG-3'
HMBS FW	5'-GACTATGCAGGCCACCATCC-3'
HMBS REV	5'-TCCAACCAGTTGTGGGTCAT-3'
**Primers for genotyping**	
hSIRT6-V5 FW	5'-GGAGCCCAAGGAGGAATCT-3'
hSIRT6-V5 REV	5'-AGACCGAGGAGAGGGTTAGG-3'
ApoE FW	5'-GCCTAGCCGAGGGAGAGCCG-3'
ApoE REV WT	5'-TGTGACTTGGGAGCTCTGCAGC-3'
ApoE REV mutant	5'-GCCGCC CCGACTGCATCT-3'
**Human Taqman probes**	
SIRT6	Hs00966002_m1
CHIP/STUB1	Hs01071598_g1
18S	Hs03003631_g1

Table II. Antibodies used for Western blotting and (chromatin) immunoprecipitation.

**Antibody**	**Catalogue no**	**Company**	**Species**	**Dilution**	**Application**
SIRT6 (human)	2590	CST	Rabbit	1/1000	WB
SIRT6	12486	CST	Rabbit	1/1000	WB
SIRT6	ab62739	Abcam	Rabbit	3μg/1.5μl	ChIP
SIRT6	MA5-24768	Thermo Scientific	Mouse	1/100	IP
SIRT1	2493	CST	Rabbit	1/1000	WB
Histon 3	3638	CST	Mouse	1/1000	WB
Histon 3	9715	CST	Rabbit	1/1000	WB
H3K9ac	9649	CST	Rabbit	1/1000<br>1/50	WB<br>ChIP
H3K27ac	4353	CST	Rabbit	1/1000	WB
V5-tag	13202	CST	Rabbit	1/1000	WB
Myc-tag	2272	CST	Rabbit	1/1000	WB
Ubiquitin	ab7780	Abcam	Rabbit	1/500	WB
CHIP	2080	CST	Rabbit	1/1000	WB
p16	10883-1-AP	ProteinTech	Rabbit	1/1000	WB
P-c-Jun (Ser73)	3270	CST	Rabbit	1/1000	WB
c-Jun	9165	CST	Rabbit	1/1000	WB
P-p38 (Thr180/Tyr182)	9211	CST	Rabbit	1/1000	WB
p38	ab31828	abcam	Mouse	1/1000	WB
P-Erk1/2 (Thr202/Tyr204)	4370	CST	Rabbit	1/1000	WB
Erk1/2	4695	CST	Rabbit	1/1000	WB
CD36	18836-1-AP	Proteintech	Rabbit	1/1000	WB
P-NFκB p65 (Ser536)	3033	CST	Rabbit	1/500	WB
Total NFκB p65	8242	CST	Rabbit	1/1000	WB
γ-H2AX (Ser139)	2577	CST	Rabbit	1/500	WB
53BP1	NB100-304	Novus	Rabbit	3μg/3μl	ChIP
TRF1	ab66223	Abcam	Mouse	1/2000	WB
TRF2	14824	CST	Rabbit	1/500	WB
β-actin	A5-441<br>Clone AC-15	Sigma	Mouse	1/10 000	WB

Table III. Organ weights, lipids, serum cytokines and peripheral blood counts in experimental mice.

	WT	S6	S6H
**Heart/body weight**	4.76 ± 0.27	4.95 ± 0.20	5.18 ± 0.29
**Liver weight (g)**	1402 ± 78	1536 ± 76	1337 ± 55
**Spleen weight (g)**	200 ± 18	224 ± 21	193 ± 23
**Total chol (mmol/L)**	14.56 ± 3.19	13.94 ± 0.96	14.03 ± 1.16
**c-LDL (mmol/l)**	13.69 ± 0.88	13.94 ± 0.94	14.03 ± 1.16
**HDL (mmol/l)**	0.31 ± 0.04	0.29 ± 0.03	0.29 ± 0.03
**Triglycerides (mmol/l)**	1.29 ± 0.53	1.28 ± 0.30	0.93 ± 0.16
**Systolic BP (mmHg) pre-HFD**	131.1 ± 4.2	120.8 ± 4.2	126.0 ± 2.9
**Diastolic BP (mmHg) pre-HFD**	65.3 ± 4.4	61.8 ± 5.4	60.4 ± 2.0
**Systolic BP (mmHg) post-HFD**	111.6 ± 3.4	113.7 ± 2.6	108.6 ± 3.2
**Diastolic BP (mmHg) post-HFD**	56.6 ±2.9	58.4 ± 6.5	57.1 ± 2.9
**IL-5 (pg/ml)**	3.32 ± 0.29	3.32 ± 0.64	4.23 ± 0.69
**IL-6 (pg/ml)**	126.8 ± 28.2	144.3 ± 28.5	89.60 ± 14.90
**IL-10 (pg/ml)**	51.17 ± 9.13	39.25 ± 3.03	35.75 ± 8.46
**TNFα (pg/ml)**	28.58 ± 3.42	29.75 ± 4.03	24.00 ± 1.50
**KCGRO (pg/ml)**	110.7 ± 13.9	114.8 ± 21.7	79.2 ± 10.1
**Platelets total**	1099 ± 110	914 ± 78	1204 ± 142
**RBC total**	10.43 ± 0.61	10.36 ± 0.39	9.30 ± 0.56
**WBC total**	6.31 ± 0.64	7.83 ± 0.76	7.15 ± 0.74
**Lymphocytes**	4.01 ± 0.45	4.90 ± 0.47	4.19 ± 0.42
**Monocytes**	0.355 ± 0.064	0.482 ± 0.088	0.390 ± 0.067
**Granulocytes**	1.944 ± 0.259	2.445 ± 0.329	2.570 ± 0.325

Parameters of SM22α-SIRT6/ApoE$^{-/-}$ mice (n=16), SM22α-hSIRT6^{H133Y}/ApoE$^{-/-}$ mice (n=13) and WT (ApoE$^{-/-}$) littermate controls (n=13) fed a HFD for 16 weeks. From top to bottom: Heart/body weight, liver weight and spleen weight. Total cholesterol, calculated low density lipoproteins (c-LDL), high density lipoproteins (HDL) and triglycerides in serum after 16 weeks of HFD (n=12). Systolic and diastolic blood pressure measurements by tail-cuff method at start and end of HFD (n=8). Serum cytokines (IL-5, IL-6, IL-10, TNFα, KCGRO) at end of HFD (n=12). IL-2, IL-4, IL12p70 and IFNγ were below the limit of detection. Platelets, red blood cells (RBC), white blood cells (WBC), total lymphocytes, monocytes and granulocytes counts (n=10). 1-way ANOVA, P>0.05. Data are mean ± SEM.
